# Checklist of British and Irish Hymenoptera - Cynipoidea

**DOI:** 10.3897/BDJ.5.e8049

**Published:** 2017-03-09

**Authors:** Mattias Forshage, Jeremy Bowdrey, Gavin R. Broad, Brian M. Spooner, Frank van Veen

**Affiliations:** 1 Swedish Museum of Natural History, Stockholm, Sweden; 2 Colchester and Ipswich Museums, Colchester, United Kingdom; 3 The Natural History Museum, London, United Kingdom; 4 Royal Botanic Gardens, Kew, Richmond, United Kingdom; 5 University of Exeter, Penryn, United Kingdom

## Abstract

**Background:**

The British and Irish checklist of Cynipoidea is revised, considerably updating the last complete checklist published in 1978. Disregarding uncertain identifications, 220 species are now known from Britain and Ireland, comprising 91 Cynipidae (including two established non-natives), 127 Figitidae and two Ibaliidae.

**New information:**

One **replacement name** is proposed, ***Kleidotoma thomsoni* Forshage**, for the secondary homonym *Kleidotoma tetratoma* Thomson, 1861 (nec *K. tetratoma* (Hartig, 1841)).

## Introduction

This paper continues the series of updated British and Irish Hymenoptera checklists that started with Broad and Livermore (2014a), Broad and Livermore (2014b), Liston et al. (2014) and with an introduction by Broad (2014). This represents the first complete update of the British list since 1978 (Fitton et al. 1978).

The Cynipoidea is a rather diverse superfamily of three British families (the Austrocynipidae and Liopteridae are entirely extralimital). The Figitidae and Ibaliidae comprise koinobiont endoparasitoids of other insects whereas the Cynipidae are gall-formers, or inquilines of gall-formers. A summary of cynipoid biology can be found in Gauld and Bolton (1988). Briefly, many Figitidae are parasitoids of Diptera larvae although the Charipinae are hyperparasitoids of primary parasitoids in aphids and some other Homoptera and the Anacharitinae are parasitoids of Hemerobiidae (Neuroptera); the small family Ibaliidae are specialised parasitoids of Siricidae larvae; and the Cynipidae are entirely phytophagous, most conspicuously as gall-formers of oaks (*Quercus*) but with numerous species galling other Rosaceae (and a few other plants) and the tribe Synergini are inquilines of other cynipid galls. A few representative Cynipoidea are illustrated in Figs 1, 2, 3.

The British Cynipidae checklist was updated comparatively recently (Spooner and Bowdrey 2000) and Irish checklists were published by O'Connor and Nash (1998), O'Connor et al. (2003), O'Connor (2004), O'Connor et al. (2009) and O'Connor et al. (2009). Our knowledge of the figitid fauna is still far from perfect but recent work by M. Forshage on the European Eucoilinae and a catalogue of Charipinae (Ferrer-Suay et al. 2012b plus updated synonymy in several other papers) have been timely. MF has sorted and identified the BMNH collections of British Eucoilinae, adding many new species and distribution records. In contrast to the rather neglected Figitidae and Ibaliidae, gall wasps (Cynipidae) have received a certain amount of attention over the years and are studied and recorded under the auspices of the British Plant Gall Society. The numbers of valid, certainly identified Cynipoidea are listed by family and country in Table 1. The total fauna has increased by 9% since the last checklist (Quinlan 1978b). This rather small increase can be explained by the substantial increase in taxonomic work in recent years, resulting in synonymy and corrected identifications nearly keeping pace with new discoveries.

## Materials and methods

We reference all additions to and deletions from the 1978 British list (Quinlan 1978a) and record country-level distribution within the British Isles, i.e. England, Scotland, Wales, Ireland (as one unit) and the Isle of Man. A more complete introduction to the methods and rationale behind this checklist series can be found in Broad (2014).

Because the agamic and sexual generations of cynipids are sometimes referred to by different names, these are differentiated in the checklist. The following conventions and abbreviations are used:

[***species***] taxon deleted from the British and Irish list and nomina dubia

BMNH Natural History Museum, London

# known introductions occurring only under artificial conditions or established in the wild

? status (including uncertain synonymy) or identification in the British Isles uncertain

misident. has been misidentified as this name

nom. dub. *nomen dubium*, a name of doubtful status

nom. ob. *nomen oblitum*, ‘forgotten name’, does not have priority over a younger name

nom. nov. *nomen novum*, a replacement name

nom. nud. *nomen nudum*, an unavailable name, with no type specimen

preocc. name preoccupied (junior homonym)

stat. rev. *status revocatus*, revived status (e.g. raised from synonymy)

unavailable name unavailable under provisions of the zoological code

var. variety, only available as a valid name under certain provisions of the zoological code

f. form, only available as a valid name under certain provisions of the zoological code

-a- name based on agamic (asexual) generation (used in Cynipidae)

-s- name based on sexual generation (used in Cynipidae)

Distribution data for Cynipidae are mainly derived by JPB from published sources, but thanks are due to the following for supplying additional data: Janet Boyd, Records Data Manager, British Plant Gall Society; Adrian Fowles, Countryside Council for Wales; Kate Hawkins, Manx Natural Heritage; David Notton, BMNH; Mark Pavett, National Museum of Wales (all pers. comm.). Distribution data for Figitidae and Ibaliidae are mainly from BMNH and cited published sources.

Word document and Excel spreadsheet versions of the checklist are available as supplementary files: Suppl. materials 1, 2.

## Checklists

### Cynipidae

#### Cynipidae

Latreille, 1802

##### Notes

Liljeblad and Ronquist (1998), Ronquist (1999) and Ronquist et al. (2015) employed a series of monophyletic tribes, in the absence of strong evidence for relationships above this level. Synonymy for Cynipidae includes all names that have appeared in the British literature but does not necessarily include all Palaearctic names proposed as varieties or forms. For complete synonymy please see Melika (2006a) and Melika (2006b), also Nieves-Aldrey (2001). It should be borne in mind that future molecular studies may change our understanding of some species concepts and their alternating generations.

#### Aulacideini

Nieves-Aldrey, 1994

##### Notes

The availability of the name Aulacideini has been questioned and is currently being looked into. This will possibly only be solved by the ICZN.

#### Aulacidea

Ashmead, 1897

PSEUDAULAX Ashmead,1903

##### Notes

Species of *Aulacidea* removed from the British and Irish list:

[*andrei* (Kieffer, 1900, *Aulax*)] Added by Bagnall (1931) from the gall. Discussed by Bowdrey (1999), who concluded that this record was in error.

#### Aulacidea follioti

Barbotin, 1972

##### Distribution

England

##### Notes

added by Bowdrey (1994).

#### Aulacidea hieracii

(Linnaeus, 1758)

Cynips hieracii Linnaeus, 1758
sabaudi (Hartig, 1840, *Aylax*)
graminis (Cameron, 1875, *Aulax*)
artemisiae (Thomson, 1877, *Aylax*)
crassinervis (Thomson, 1877, *Aylax*)
foveigera (Thomson, 1877, *Aylax*)

##### Distribution

England, Scotland, Wales

#### Aulacidea nibletti

Quinlan & Askew, 1969

##### Distribution

Scotland

#### Aulacidea pilosellae

(Kieffer, 1901)

Aulax pilosellae Kieffer, 1901

##### Distribution

England

#### Aulacidea subterminalis

Niblett, 1946

##### Distribution

England

#### Aulacidea tragopogonis

(Thomson, 1877)

Aulax tragopogonis Thomson, 1877
pigeoti (Kieffer, 1898, *Aulax*)

##### Distribution

England

##### Notes

*A. pigeoti*, added by Bagnall and Harrison (1930) on the basis of galls only (root collar of *Tragopogon porrifolius*), was synonymised by Eady and Quinlan (1963) with *tragopogonis* but erroneously so with *hieracii* in Fauna Europaea (Noyes et al. 2004) (Nieves-Aldrey, pers. comm.).

#### Isocolus

Förster, 1869

EUBOTHRUS Förster, 1869

#### Isocolus fitchi

(Kieffer, 1898)

Aulax fitchi Kieffer, 1898

##### Distribution

England

#### Isocolus jaceae

(Schenck, 1863)

Aulax jaceae Schenck, 1863
affinis (Schenck, 1863, *Aylax*)

##### Distribution

England, Scotland

#### Isocolus scabiosae

(Giraud, 1859)

Diastrophus scabiosae Giraud, 1859
areolatus (Giraud, 1859, *Diastrophus*)
centaureae (Thomson, 1877, *Aulax*)
rogenhoferi Wachtl, 1880

##### Distribution

England

##### Notes

*rogenhoferi*: galls bracts of *Centaurea scabiosa*, but not now considered to be a distinct species (Nieves-Aldrey 1994).

#### Liposthenes

Förster, 1869

LIPOSTHENUS misspelling

#### Liposthenes glechomae

(Linnaeus, 1758)

Cynips glechomae Linnaeus, 1758
latreillei (Kieffer, 1898, *Aulax*)

##### Distribution

England, Scotland, Wales, Isle of Man

#### Aylacini

Ashmead, 1903

#### Aylax

Hartig, 1840

AULAX Hartig, 1843

#### Aylax minor

Hartig, 1840

##### Distribution

England, Ireland

#### Aylax papaveris

(Perris, 1840)

Diplolepis papaveris Perris, 1840
rhoeadis (Bouché, 1834, *Cynips*)

##### Distribution

England, Scotland, Wales

#### Ceroptresini

Nieves-Aldrey, Nylander & Ronquist, 2015

#### Ceroptres

Hartig, 1840

#### Ceroptres cerri

Mayr, 1873

Ceroptres cerriphilus Giraud, 1911
vitripennis Giraud, 1911

##### Distribution

England

##### Notes

Added by Jennings Jennings (2016).

#### Ceroptres clavicornis

Hartig, 1840

socialis Hartig, 1840
arator Hartig, 1841
melanonerus Hartig, 1841

##### Distribution

England, Scotland, Wales, Ireland

#### Cynipini

Latreille, 1802[4]

##### Notes

Generic classification of oak-associated Cynipini follows Melika and Abrahamson (2002). As sexual and agamic generations have often been described under different names, those based on agamic generation are denoted by the suffix '-a-', those based on the sexual generation by '-s-'.

#### Andricus

Hartig, 1840

APHILOTHRIX Förster, 1869
LIODORA Förster, 1869
MANDERSTJERNIA Radoszkowski, 1866
TRICHOTERAS Ashmead, 1897
PARANDRICUS Kieffer, 1906
ADLERIA Rohwer & Fagan, 1917
EUSCHMITZIA Dettmer, 1925
ONCASPIS Dettmer, 1925
DROS Kinsey,1937
DRUON Kinsey, 1937
FERON Kinsey, 1937
CONOBIOS Kinsey, 1938

##### Notes

Species of *Andricus* removed from the British and Irish list:

[*clementinus* (Giraud, 1859, *Cynips*) -a-] Added by Cameron (1893) from the gall only. An agamic female in the Morley collection at Ipswich Museum is *quercusradicis*. No recent records.

[*gallaetinctoriae* (Olivier, 1791, *Diplolepis*) -a-, syn. *tinctoria* (Hartig, 1843, *Cynips*) -a-] Niblett et al. (1932) (following Houard) state that this species was accidentally introduced in the 19th Century on *Quercus aegilops*, and never established.

[*gallaeurniformis* (Boyer de Fonscolombe, 1832, *Diplolepis*) -a-, syn. *sufflator* Mayr, 1882 -s-] First recorded from old galls by Bagnall and Harrison (1918). Sexual generation also included by Eady and Quinlan (1963), doubtfully British, no confirmed records.

[*quercustozae* (Bosc, 1792, *Cynips*) -a-] Added by Eady and Quinlan (1967) on the basis of a single gall found on a road at Kew. No further records.

[*trotteri* Kieffer, 1898 -a-] Added by Bagnall and Harrison (1918) from the gall only. No further records and rejected as British by Eady and Quinlan (1963).

#### Andricus amenti

Giraud, 1859 -s-

callidoma (Giraud, 1859, *Cynips*) preocc. -a-
giraudianus Dalla Torre & Kieffer, 1910 -a-

##### Distribution

England, Scotland

##### Notes

Sexual generation catkin galls first recorded by Traill (1873). The asexual generation, described as *giraudianus* Dalla Torre & Kieffer, 1910, has not been recorded in Britain.

#### Andricus aries

(Giraud, 1859) -a-

Cynips aries Giraud, 1859

##### Distribution

England, Wales

##### Notes

Added by Leach (1999). First recorded from the agamic generation, the sexual generation was reported by Walker (2001b).

#### Andricus callidoma

(Hartig, 1841) -a-

Cynips callidoma Hartig, 1841
cirratus Adler, 1881 -s-
giraudi Wachtl, 1882 -a-

##### Distribution

England, Scotland, Wales, Ireland

#### Andricus corruptrix

(Schlechtendal, 1870) -a-

Cynips corruptrix Schlechtendal, 1870
ambigua (Trotter, 1899, *Cynips*) -a-
corruptrix f. elianae Melika, Csóka & Pujade-Villar, 2000 nom. nud. -s-

##### Distribution

England, Scotland, Ireland

##### Notes

*Cynips ambigua* was placed by Bellido et al. (2005) in synonymy with *corruptrix* but previously regarded as a distinct species (Melika et al. 2000). Agamic generation reported by Harrison (1930) from Co. Durham and Forfarshire. The sexual generation was described as forma *elianae*. Form *larshemi* Leeuwen and Dekhuijzen-Maasland (1958), was described as the sexual generation of *corruptrix* but is now believed to be *improprius* Bellido & Pujade-Villar, 2004, fide Folliot et al. (2004), who also produced the true sexual generation of *corruptrix* experimentally.

#### Andricus cryptobius

Wachtl, 1880 -s-

##### Distribution

England

##### Notes

Added by Bowdrey (2015); an adult female was identified as this species by G. Melika with the proviso that it might be the sexual generation of a morphologically undescribed species known from DNA sequencing.

#### Andricus curvator

Hartig, 1840 -s-

axillaris (Hartig, 1840, *Cynips*) -s-
collaris (Hartig, 1840, *Cynips*) -a-
roeselii (Dahlbom, 1842, *Cynips*) (nom. nud.) -s-
dimidiatus (Schenck, 1863, *Spathegaster*) -s-
fasciatus Schenck, 1863 -a-
perfoliatus Schenck, 1863 -s-
sulcata (Förster, 1869, *Liodora*) -s-
fasciata (Schlechtendal, 1870, *Cynips*) -a-
tegmentorum (Schlechtendal, 1870, *Cynips*) -a-

##### Distribution

England, Scotland, Wales, Ireland, Isle of Man

#### Andricus foecundatrix

(Hartig, 1840) -a-

Cynips foecundatrix Hartig, 1840
fecundator misspelling
quercusgemmae (Linnaeus, 1758, *Cynips*)
gemmarum Lacaze-Duthiers, 1853 -a-
gemmae (Schenck, 1863, *Cynips*) -a
pilosus Adler, 1881 -s-

##### Distribution

England, Scotland, Wales, Ireland, Isle of Man

#### Andricus gemmeus

(Giraud, 1859) -a-

Cynips gemmea Giraud, 1859
kirschbergi (Wachtl, 1876, *Aphilothrix*) -a-
gemmae Dalla Torre & Kieffer, 1910 -a-

##### Distribution

England

##### Notes

Added by Bowdrey (2009). Previously reported by Ormerod (see Cameron 1893), but reportedly as a leaf gall, so the record is suspect.

#### Andricus gemmicola

Kieffer, 1901

##### Distribution

England

##### Notes

Gall recorded by Bagnall and Harrison (1919) and adult male possibly of this species by Eady and Quinlan (1963). Only the sexual generation is known, inducing bud galls of Quercus robur, collected in France (Dalla Torre and Kieffer 1910). Bagnall and Harrison (1918) mentioned this species from Great Britain but this record requires confirmation (Eady and Quinlan 1963). A species of uncertain status, type probably lost.

#### Andricus glandulae

(Hartig, 1840) -a-

Cynips glandulae Hartig, 1840
xanthopsis Schlechtendal, 1884 -s-

##### Distribution

England, Scotland, Ireland

#### Andricus grossulariae

Giraud, 1859 -s-

mayri (Wachtl, 1879, *Aphilothrix*) -a-
panteli Kieffer, 1896 -a-
gemellus Belizin & Maisuradze, 1961 -s-

##### Distribution

England

##### Notes

Added by Walker (2001a). Rearing experiments by Walker (2002) confirmed *grossulariae* to represent the sexual generation of a lifecycle also involving the asexual generation galls known as *mayri/ panteli*, a pairing also indicated by DNA sequence data (Stone et al. 2008).

#### Andricus inflator

Hartig, 1840 -s-

globuli (Hartig, 1841, *Cynips*) -s-
inflatioides Belizin & Maisuradze, 1962 -?-

##### Distribution

England, Scotland, Wales, Ireland

#### Andricus kollari

(Hartig, 1843) -a-

Cynips kollari Hartig, 1843
lignicola auctt. misident.
quercusgemmae (Christ, 1791, *Cynips*) preocc. -a-
circulans Mayr, 1870 -s-

##### Distribution

England, Scotland, Wales, Ireland, Isle of Man

##### Notes

Nineteenth Century introduction (Adler and Stratton 1894; Niblett et al. 1932).

#### Andricus legitimus

Wiebes-Rijks, 1980 -a-

##### Distribution

England

#### Andricus lignicolus

(Hartig, 1840) -a-

Cynips lignicola Hartig, 1840
var. vanheurni van Leeuwen & Dekhuizen-Maasland, 1958 -s-

##### Distribution

England, Scotland, Wales, Ireland, Isle of Man

#### Andricus lucidus

(Hartig, 1843) -a-

Cynips lucida Hartig, 1843
aestivalis Giraud, 1859 -s-
erinaceus (Kieffer, 1900, *Adleria*) -a-

##### Distribution

England

##### Notes

Added by Mosley (1892) from Yorkshire and by (Cameron 1893) from Loch Lomond (sexual form). Not recorded subsequently until reinstated as a British species, from London specimens, by Stone and Sunnocks (1992).

#### Andricus malpighii

(Adler, 1881) -a-

Aphilothrix malpighii Adler, 1881
nudus Adler, 1881 -s-

##### Distribution

England, Scotland, Wales, Ireland

##### Notes

Nomenclature follows Pujade-Villar and Melika (2000).

#### Andricus paradoxus

(Radoszkowski, 1866) -a-

Manderstjernia paradoxa Radoszkowski, 1866
albipunctata (Kaltenbach, 1867, *Cynips*) -a-
majalis (Girauld, 1868, *Cynips*) preocc. -a-
albopunctata (Schlechtendal, 1870, *Cynips*) -a-
lambertoni Kieffer, 1897 -a-
albopunctatus f. barbotini Folliot, 1964 -s-

##### Distribution

England, Scotland, Wales, Ireland

##### Notes

Taxonomy follows Melika et al. (2000).

#### Andricus quadrilineatus

Hartig, 1840 -a-

ambiguus Schenck, 1863 -a-
glabriusculus Schenck, 1863 -a-
pedunculi Schenck, 1863 -a-
verrucosus Schenck, 1863 -a-
marginalis (Schlechtendal, 1870, *Cynips*) -a-
4-lineata (Thomson, 1877, *Cynips*) -a-
kiefferi Pigeot, 1900 -s-
quadrilineatus f. *kiefferi* Folliot, 1964 -s-

##### Distribution

England, Scotland, Wales, Ireland

##### Notes

Synonymy of *marginalis* and *quadrilineatus* established by Folliot (1964).

#### Andricus quercuscalicis

(Burgsdorff, 1783) -a-

Cynips quercuscalicis Burgsdorff, 1783
cerri Beyerinck, 1896 -s-
beyerincki Trotter, 1899 -s-

##### Distribution

England, Scotland, Wales, Ireland

#### Andricus quercuscorticis

(Linnaeus, 1761) -a-

Cynips quercuscorticis Linnaeus, 1761
corticis (Hartig, 1840, *Cynips*) unjustified emendation -a-
brevicornis (Hartig, 1841, *Cynips*) -s-
gemmatus Adler, 1881 -s-
krajnovici Tavares, 1901 -a-

##### Distribution

England, Scotland, Wales, Ireland, Isle of Man

#### Andricus quercusradicis

(Fabricius, 1798) -a-

Cynips quercusradicis Fabricius, 1798
noduli Hartig, 1840 -s-
trilineatus Hartig, 1840 -s-
parasiticus (Hartig, 1841, *Neuroterus*) -s-
radicis (Hartig, 1841, *Cynips*) unjustified emendation -a-
rugiscuta Thomson, 1877 -s-

##### Distribution

England, Scotland, Wales, Ireland

#### Andricus quercusramuli

(Linnaeus, 1761) -s-

Cynips quercusramuli Linnaeus, 1761
autumnalis (Hartig, 1840, *Cynips*) -a-
amentorum (Hartig, 1843, *Teras*)
ramuli Schenck, 1863 unjustified emendation -s-

##### Distribution

England, Scotland, Wales, Ireland

#### Andricus rhyzomae

(Hartig, 1843) -a-

Cynips rhyzomae Hartig, 1843
ionescui Kierych, 1965 -a-

##### Distribution

England, Wales

##### Notes

Gall recorded by Bagnall and Harrison (1918) and Burkill (1933). Modern UK status uncertain. Galls of the sexual generation unconfirmed in Britain. See also under *testaceipes*.

#### Andricus seminationis

(Giraud, 1859) -a-

Cynips seminationis Giraud, 1859
inflorescentiae (Schlechtendal, 1870, *Cynips*) -?-

##### Distribution

England, Scotland, Wales

##### Notes

No sexual generation known.

#### Andricus sieboldi

(Hartig, 1843) -a-

Cynips sieboldi Hartig, 1843
corticalis (Hartig, 1840, *Cynips*) -a-
quercuscorticis (Bechstein & Scharfenberg, 1805, *Cynips*) preocc. -a-
occidentalis Folliot, 1964 -a-
sieboldi f. poissoni Folliot, 1964 -s-

##### Distribution

England, Wales

##### Notes

sieboldi f. poissoni Folliot, 1964: sexual generation, produced experimentally.

#### Andricus singularis

Mayr, 1870 -s-

singulus Mayr, 1881 -s-

##### Distribution

England

##### Notes

added by Jennings (2014)

#### Andricus solitarius

(Boyer de Fonscolombe, 1832) -a-

Diplolepis solitarius Boyer de Fonscolombe, 1832
ferruginea (Hartig, 1840, *Cynips*) -a-
occultus Tschek, 1871 -s-
gallaepyriformis (Olivier, 1791, *Diplolepis*) -a-
filigranata (Dettmer, 1925, *Oncaspis*) -s-
villarrubiae Tavares, 1930 -a-

##### Distribution

England, Scotland, Wales, Ireland

#### Andricus testaceipes

Hartig, 1840 -s-

var. nodifex Kieffer, 1900 -s-

##### Distribution

England?

##### Notes

Maintained with a query; possibly the unconfirmed sexual generation of *rhyzomae* (q.v.). If specifically distinct, British records need confirmation. See Melika (2006a).

#### Aphelonyx

Mayr, 1881

#### Aphelonyx cerricola

(Giraud, 1859) -a-

Cynips cerricola Giraud, 1859

##### Distribution

England

##### Notes

added by Crawley (1997)

#### Biorhiza

Westwood, 1840

APOPHYLLUS Hartig, 1840
TERAS Hartig, 1840 preocc.
PHILONIPS Walsh, 1864
HETEROBIUS Guérin-Méneville, 1865
DRYOTERAS Förster, 1869
HARTIGIA Rondani, 1871 preocc.

#### Biorhiza pallida

(Olivier, 1791) -s-

Diplolepis pallidus Olivier, 1791
aptera (Bosc, 1791, *Cynips*) preocc. -a-
gallaealvaeriformis (D'Anthoine, 1794, *Diplolepis*) -s-
gallaecerebriformis (D'Anthoine, 1794, *Diplolepis*) -s-
quercusterminalis (Fabricius, 1798, *Cynips*) -s-
terminalis (Hartig, 1840, *Teras*) unjustified emendation -s-
sieboldi (Stadelman, 1892, *Andricus*) preocc. -a-

##### Distribution

England, Scotland, Wales, Ireland, Isle of Man

#### Callirhytis

Förster, 1869

EUSYMPHAGUS Dettmer, 1930

##### Notes

Species of *Callirhytis* removed from the British and Irish list:

[*glandium* (Giraud, 1859, *Andricus*) -a-] Recorded by Rolfe (1881) and Askew (1959) but in error fide Eady and Quinlan (1963).

#### Callirhytis bella

(Dettmer, 1930) -s-

Eusymphagus belllus Dettmer, 1930

##### Distribution

England, Wales, Ireland

#### Callirhytis erythrocephala

(Giraud, 1859) -a-

Andricus erythrocephala Giraud, 1859
? erythrosoma Dettmer, 1933 -a-
erythrostoma Dettmer, 1933 -a-
hartigi Förster, 1869 -s- synonymy by Pujade-Villar et al. (2007)

##### Distribution

England, Wales

##### Notes

*Callirhytis hartigi* Förster, 1869 established as the sexual generation by Pujade-Villar et al. (2007), who produce evidence that *erythrosoma* may be a separate species.

#### Cynips

Linnaeus, 1758

DIPLOLEPIS misapplied
DRYOPHANTA Förster, 1869

##### Notes

Species of *Cynips* excluded from the British and Irish list:

[*quercus* (Fourcroy, 1785, *Diplolepis*) -a-; syn. *pubescentis* (Mayr, 1881, *Dryophanta*) -s-] Both generations reported by Bagnall and Harrison (1918), Bagnall and Harrison (1919) from several localities in Northumberland and Tyne & Wear, perhaps in error due to confusion with the similar *quercusfolii*. There are no subsequent records and the species requires confirmation as British.

#### Cynips agama

Hartig, 1840 -a-

f. mailleti Folliot, 1964 -s-

##### Distribution

England, Scotland, Wales, Ireland

#### Cynips disticha

Hartig, 1840 -a-

f. indistincta Niblett, 1948 -s-

##### Distribution

England, Scotland, Wales, Ireland

#### Cynips divisa

Hartig, 1840 -a-

verrucosus (Schlechtendal, 1870, *Spathegaster*) -s-

##### Distribution

England, Scotland, Wales, Ireland

#### Cynips longiventris

Hartig, 1840 -a-

similis (Adler, 1881, *Spathegaster*) -s-
f. substituta Kinsey, 1930 -s-

##### Distribution

England, Scotland, Wales, Ireland

#### Cynips quercusfolii

Linnaeus, 1758 -a-

floriiquercus Gleditsch, 1774 -a-
scutellaris (Olivier, 1791, *Diplolepis*) -a-
gallaecerasiformis D'Anthoine, 1794 -a-
gallaeunedoniformis (D'Anthoine, 1794, *Diplolepis*) -a-
flosculi (Giraud, 1868, *Spathegaster*) -s-
giraudi (Tschek, 1869, *Spathegaster*) -s-
taschenbergi (Schlechtendal, 1870, *Spathegaster*) -s-

##### Distribution

England, Scotland, Wales, Ireland

##### Notes

Galls of the sexual generation were recorded as *flosculi* by Bagnall and Harrison (1918); there appear to be no further records.

#### Dryocosmus

Giraud, 1859

##### Notes

Species of *Dryocosmus* removed from the British and Irish list:

[*cerriphilus* Giraud, 1859 -a-] Galls recorded once by Fitch (1874); no further UK records.

#### Dryocosmus kuriphilus

Yasumatsu, 1951

##### Distribution

England

##### Notes

Added by Malumphy (2015), EPPO (2015). This highly invasive, East Asian species has spread across Europe and has recently been reported from Kent and Hertfordshire. As the UK has Protected Zone Status for this global pest, apparently successful attempts were made to eradicate *D. kuriphilus* at the two outbreak sites and it should not be formally added to the British list at this stage.

#### Neuroterus

Hartig, 1840

SPATHEGASTER Hartig, 1840
AMERISTUS Förster, 1869
DOLICHOSTROPHUS Ashmead, 1887
NEOSPATHEGASTER Kinsey, 1923
DIPLOBIUS Kinsey, 1923
NEONEUROTERUS Monzen, 1954
REPENTINIA Belizin & Maisuradze, 1961

##### Notes

Species of *Neuroterus* removed from the British and Irish list:

[*punctatus* (Bignell, 1892, *Spathegaster*)] Described by Bignell (1892), no subsequent records.

#### Neuroterus albipes

(Schenck, 1863) -s-

Spathegaster albipes Schenck, 1863
laeviusculus Schenck, 1863 -a-
pezizaeformis Schlechtendal, 1870
codinae Tavares, 1928 -s-

##### Distribution

England, Scotland, Wales, Ireland, Isle of Man

##### Notes

Form ‘*borealis*’ described by Entwistle and Hails (1997).

#### Neuroterus anthracinus

(Curtis, 1838) -a-

Cynips anthracina Curtis, 1838
flavipes (Boyer de Fonscolombe, 1832, *Diplolepis*) -a- nom. ob.
ostrea (Hartig, 1840, *Cynips*) -a-
furunculus Beyerinck, 1882 -s-

##### Distribution

England, Scotland, Wales, Ireland

##### Notes

Transferred from *Andricus* by Pujade-Villar et al. (1998).

#### Neuroterus numismalis

(Geoffroy, 1785) -a-

Cynips numismalis Geoffroy, 1785
numismatis (Olivier, 1790, *Cynips*) -a-
defectus Hartig, 1840 -a-
reaumuri Hartig -a-
quercustiarae (Curtis, 1843, *Cynips*)
nigricornis Schenck, 1863 -a-
vesicatrix (Schlechtendal, 1870, *Cynips*) -s-
vesicator Hieronymus, 1890 -s-
brunneus Dettmer, 1925 -s-

##### Distribution

England, Scotland, Wales, Ireland

#### Neuroterus politus

Hartig, 1840 -s-

petioliventris (Hartig, 1840, *Spathegaster*) -s-
bipunctatus Hartig, 1841 -s-
nitens Hartig, 1841 -a-
rubeculus Hartig, 1841 -a-
aprilinus (Giraud, 1859, *Spathegaster*) -s-
schlechtendali Mayr, 1870 -a-
burgundus (Schlechtendal, 1870, *Andricus*) preocc. -a-
schlechtendali Mayr, 1870 -a-

##### Distribution

England, Scotland, Ireland

#### Neuroterus quercusbaccarum

(Linnaeus, 1758) -s-

Cynips quercusbaccarum Linnaeus, 1758
quercuspedunculi (Linnaeus, 1758, *Cynips*) -s-
baccarumquercus (Fourcroy, 1785, *Cynips*) -s-
pedunculiquercus (Fourcroy, 1785, *Cynips*) -s-
lenticularis (Olivier, 1791, *Diplolepis*) -a-
longipennis (Fabricius, 1793, *Cynips*) -a-
gallaelenticulae (D'Anthoine, 1794, *Diplolepis*) -a-
gallaepisiformis (D'Anthoine, 1794, *Diplolepis*) -s-
malpighii Hartig, 1840 -a-
interruptrix (Hartig, 1840, *Cynips*) -s-
interruptor (Hartig, 1841, *Spathegaster*) -s-
baccarum (Blanchard, 1849, *Cynips*) -s-
pedunculi (Duméril, 1860, *Diplolepis*) -s-
attenuatus Schenck, 1863 -a-
striatus Schenck, 1863 -a-
pseudodisticha (Küstenmacher, 1894, *Dryophanta*) -s-

##### Distribution

England, Scotland, Wales, Ireland, Isle of Man

#### Neuroterus tricolor

(Hartig, 1841) -s-

Spathegaster tricolor Hartig, 1841
fumipennis Hartig, 1841 -a-
varius (Schenck, 1863, *Spathegaster*) -a-

##### Distribution

England, Scotland, Wales, Ireland

#### Plagiotrochus

Mayr, 1881

FIORIA Kieffer, 1903
FIORIELLA Kiefer, 1903

#### Plagiotrochus australis

(Mayr, 1882) -a-

Dryocosmus australis Mayr, 1882

##### Distribution

England, Wales

##### Notes

Added by Robbins (2007) based on old agamic gall only; subsequently found at Imperial Wharf and Royal Botanic Gardens, Kew, both London (D.G. Notton, pers. comm.). Galls introduced *Quercus ilex* and *Q. coccifera*.

#### Plagiotrochus coriaceus

(Mayr, 1882) -a-

Andricus coriaceus Mayr, 1882
pseudococcus (Kieffer, 1902, *Andricus*) -a-

##### Distribution

Wales

##### Notes

Added by Robbins (2007), based on old agamic gall only.

#### Plagiotrochus quercusilicis

(Fabricius, 1798) -s-

Cynips quercusilicis Fabricius, 1798
cocciferae (Lichtenstein, 1877, *Andricus*) -s-
ilicis (Lichtenstein, 1877, *Andricus*) -s-
emeryi Mayr, 1882 -s-
fusifex Mayr, 1882 -s-

##### Distribution

England

##### Notes

Added by Hancy and Hancy (2004). Added from sexual generation gall, adults reared. Recent records from Devon, Cornwall and Hampshire (Isle of Wight). Catkin galls reported by Biggs (2011) from Isle of Wight as *forma fusifex*, which has no taxonomic status.

#### Pseudoneuroterus

Kinsey, 1923

#### Pseudoneuroterus saliens

(Kollar, 1857) -a-

Cynips saliens Kollar, 1857
saltans (Giraud, 1859, *Neuroterus*) -a-
glandiformis (Giraud, 1859, *Spathegaster*) -s-

##### Distribution

England

##### Notes

added by Redfern (2006)

#### Trigonaspis

Hartig, 1840

XANTHOTERAS Ashmead, 1897
BELIZINELLA Kovatev, 1965
USSURASPIS Kovalev, 1965

##### Notes

Species of *Trigonaspis* removed from the British and Irish list:

[*synaspis* (Hartig, 1841, *Apophyllus*) -a-; syn. *megapteropsis* Wriese, 1900 -s-] Recorded by Bagnall and Harrison (1918) from the agamic gall; no further records.

#### Trigonaspis megaptera

(Panzer, 1801) -s-

crustalis Hartig, 1840 -s-
renum (Hartig, 1840, *Cynips*) -a-

##### Distribution

England, Scotland, Wales, Ireland

#### Diastrophini

Nieves-Aldrey, Nylander & Ronquist, 2015

#### Diastrophus

Hartig, 1840

GONASPIS Ashmead, 1897

##### Notes

Species of *Diastrophus* removed from the British and Irish list:

[*mayri* Reinhard, 1876] The status of *mayri* as a British species appears to rest solely on three published references to the species: Cameron (1893), subsequently quoted in Connold (1909) and repeated by Swanton (1912). Nowhere does Cameron claim to have evidence of this species’ occurrence in Britain, merely comparing its gall to other British species of *Diastrophus* and *Xestophanes*. As there appear to be no genuine UK records it should therefore be excluded.

#### Diastrophus rubi

(Bouché, 1834)

Cynips rubi Bouché, 1834
aphidivorus Cameron, 1889
hartigi (Marshall, 1867, *Andricus*)

##### Distribution

England, Wales, Ireland

#### Periclistus

Förster, 1869

#### Periclistus brandtii

(Ratzeburg, 1831)

Cynips brandtii Ratzeburg, 1831

##### Distribution

England, Scotland, Wales

#### Periclistus caninae

(Hartig, 1840)

Aylax caninae Hartig, 1840
germanus (Schenck, 1863, *Aulax*)
rosarum Dettmer, 1925

##### Distribution

England, Scotland, Ireland

#### Periclistus spinosissimae

Dettmer, 1924

##### Distribution

England, Scotland, Wales, Ireland

##### Notes

Close to *caninae*, taxonomic status needs revision (note in Fauna Europaea).

#### Xestophanes

Förster, 1869

#### Xestophanes brevitarsis

(Thomson, 1877)

Aulax brevitarsis Thomson, 1877
tormentillae Schlechtendal, 1880

##### Distribution

England, Scotland, Wales, Ireland

#### Xestophanes potentillae

(Retzius, 1783)

Cynips potentillae Retzius, 1783
brevicornis (Curtis, 1838, *Cynips*) preocc.
splendens (Hartig, 1840, *Aylax*)
laevigata Schenck, 1863
abbreviatus (Thomson, 1877, *Aulax*)
foveicollis (Thomson, 1877, *Aulax*)

##### Distribution

England, Wales, Ireland, Isle of Man

#### Phanacidini

Nieves-Aldrey, Nylander & Ronquist, 2015

#### Phanacis

Förster, 1860

GILLETTEA Ashmead, 1897

#### Phanacis caulicola

(Hedicke, 1939)

Aylax caulicola Hedicke, 1939

##### Distribution

England, Wales

#### Phanacis centaureae

(Förster, 1860)

Cynips centaureae Förster, 1860
punctipleuris (Thomson, 1877, *Aulax*)
karadagica Diakontschuk, 1980
parvulus Diakontschuk, 1980

##### Distribution

England, Scotland

#### Phanacis hypochoeridis

(Kieffer, 1887)

Aulax hypochoeridis Kieffer, 1887
seriolae Stefani, 1903

##### Distribution

England, Wales, Ireland, Isle of Man

#### Phanacis taraxaci

(Ashmead, 1897)

taraxaci Ashmead, 1897

##### Distribution

England, Scotland

##### Notes

Galls recorded from Co. Durham (Bagnall 1917), Derbyshire (Fordham 1917), Northumberland (Bagnall 1917) and Scotland (Bagnall 1932). No recent records.

#### Timaspis

Mayr, 1881

##### Notes

Synonymised with *Phanacis* (Eady and Quinlan 1963); re-established by Nieves-Aldrey (1994) but Melika (2006a) again treated *Timaspis* as a synonym of *Phanacis*.

Species of *Timaspis* removed from the British and Irish list:

[*sonchi* (De Stefani, 1900, *Aulax*)] Galls recorded from Norfolk and Surrey by Bagnall and Burkill (1935) but no recent records. Possibly confused in the literature with *Aulacidea follioti* which also galls *Sonchus asper* (Nieves-Aldrey 2001).

#### Timaspis lampsanae

(Perris, 1873)

##### Distribution

England

##### Notes

Galls recorded in Norfolk and Derbyshire by Bagnall and Harrison (1934).

#### Timaspsis lusitanica

(Tavares, 1904)

Timaspis lusitanica Tavares, 1904

##### Distribution

England

##### Notes

added by Jennings (2005)

#### Diplolepidini

Latreille, 1802

RHODITINI Hartig, 1840

#### Diplolepis

Geoffroy, 1762

RHODITES Hartig, 1840
HOLOLEXIS Förster, 1869
TRIBALIA Walsh, 1864
LYTORHODITES Kieffer, 1902
NIPPORHODITES Sakugami, 1949

#### Diplolepis eglanteriae

(Hartig, 1840)

Rhodites eglanteriae Hartig, 1840
rufipes (Förster, 1869, *Hololexis*)

##### Distribution

England

##### Notes

Galls not distinguishable from those of the smooth form of *nervosa*; records from Scotland, Wales and the Isle of Man are based on galls only.

#### Diplolepis mayri

(Schlechtendal, 1877)

Rhodites mayri Schlechtendal, 1877
orthospinae (Beijerinck, 1883, *Rhodites*)

##### Distribution

England

#### Diplolepis nervosa

(Curtis, 1838)

Cynips nervosa Curtis, 1838
centifoliae (Hartig, 1840, *Rhodites*) synonymy by Pujade-Villar and Plantard (2002)
rosarum (Giraud, 1859, *Rhodites*)
andrei (Kieffer, 1904, *Rhodites*)
kiefferi (Loiselle, 1912, *Rhodites*)
dispar (Niblett, 1943, *Rhodites*)

##### Distribution

England, Wales

#### Diplolepis rosae

(Linnaeus, 1758)

Cynips rosae Linnaeus, 1758
bedeguaris Fourcroy, 1785

##### Distribution

England, Scotland, Wales, Ireland, Isle of Man

#### Diplolepis spinosissimae

(Giraud, 1859)

Rhodites spinosissimae Giraud, 1859
rosae-spinosissimae (Inchbald, 1861, *Cynips*)

##### Distribution

England, Scotland, Wales, Ireland, Isle of Man

#### Synergini

Ashmead, 1896

##### Notes

Taxonomy follows Pujade-Villar et al. (2003).

#### Saphonecrus

Dalla Torre & Kieffer, 1910

#### Saphonecrus connatus

(Hartig, 1840)

Synergus connatus Hartig, 1840
erythroneurus (Hartig, 1840, *Synergus*)

##### Distribution

England, Scotland, Ireland

#### Synergus

Hartig, 1840

SAPHOLYTUS Förster, 1869

##### Notes

Species of *Synergus* removed from the British and Irish list:

[*hayneanus* (Ratzeberg, 1833, *Cynips*)] Added by Morley (1931); specimen in his collection at Ipswich Museum redetermined as *umbraculus* (Eady 1952).

#### Synergus apicalis

Hartig, 1841

##### Distribution

England, Scotland

#### Synergus clandestinus

Eady, 1952

##### Distribution

England, Wales, Ireland

#### Synergus consobrinus

Giraud, 1911

##### Distribution

England

##### Notes

Added by Jennings (2017); identified by G. Melika.

#### Synergus crassicornis

(Curtis, 1838)

Cynips crassicornis Curtis, 1838
evanescens Mayr, 1872
fidelis Tavares, 1920
carinulatus Dettmer, 1924

##### Distribution

England, Wales, Ireland

#### Synergus facialis

Hartig, 1840

gallaepomiformis misident.

##### Distribution

England, Scotland, Wales, Ireland

##### Notes

This species has hitherto been known as *S. gallaepomiformis* in Britain but it was shown in Pujade-Villar (2005) that this name refers to another species (of *Saphonecrus*) and the widespread species should be called *facialis*. Melika (2006a) proposed that *gallaepomiformis* (Boyer de Fonscolombe, 1832, *Diplolepis*) should be retained as the valid name but did not make an application to ICZN to overturn the type designation of *gallaepomiformis*.

#### Synergus incrassatus

Hartig, 1840

bipunctatus Hartig, 1841
crassicornis Hartig, 1843 preocc.

##### Distribution

England, Scotland, Wales, Ireland

#### Synergus pallicornis

Hartig, 1841

pallidicornis Dalla Torre, 1893

##### Distribution

England, Wales, Ireland

#### Synergus pallidipennis

Mayr, 1872

##### Distribution

England, Wales

#### Synergus pallipes

Hartig, 1840

flavicornis Hartig, 1840
nervosus Hartig, 1840
nigripes Hartig, 1840
albipes Hartig, 1841
erythrocerus Hartig, 1841
variolosus Hartig, 1841
varius Hartig, 1841
xanthocerus Hartig, 1841
tscheki Mayr, 1872
tristis Mayr, 1873
hartigi Giraud, 1911
fulvipes Dettmer, 1924
mutabilis Dettmer, 1924

##### Distribution

England, Wales, Ireland

#### Synergus radiatus

Mayr, 1872

##### Distribution

England

#### Synergus reinhardi

Mayr, 1872

##### Distribution

England, Scotland, Wales, Ireland

#### Synergus ruficornis

Hartig, 1840

##### Distribution

England, Ireland

#### Synergus thaumacerus

(Dalman, 1823)

Cynips thaumacera Dalman, 1823
klugii Hartig, 1840
carinatus Hartig, 1841
testaceus (Hartig, 1841, *Xystus*)
thaumatocerus Dalla Torre 1893 unjustified emendation
inflatus Giraud, 1911
vesiculosus Giraud, 1911
inflatus Dettmer, 1924 preocc.

##### Distribution

England

#### Synergus tibialis

Hartig, 1840

erythrostomus Hartig, 1841
immarginatus Hartig, 1841
rotundiventris Mayr, 1872

#### Synergus umbraculus

(Olivier, 1791)

Diplolepis umbraculus Olivier, 1791
gallaeumbraculatae (D'Anthoine, 1794, *Diplolepis*)
rufipes (Boyer de Fonscolombe, 1832, *Diplolepis*)
orientalis Hartig, 1841
melanopus Hartig, 1843
socialis Kollar, 1843
punctatus Dettmer, 1924 preocc.

##### Distribution

England, Scotland, Wales, Ireland

#### Synergus variabilis

Mayr, 1872

cerridis Giraud, 1911
conformis Giraud, 1911
cerricolus Vassileva-Samnalieva, 1986

##### Distribution

England

##### Notes

Added by Chinery & Williams in Melika (2006a).

### Figitidae

#### Figitidae

Hartig, 1840

##### Notes

Some Irish records from O'Connor et al. (2003).

#### Anacharitinae

Thomson, 1862

MEGAPELMINAE Förster, 1869
ACANTHAEGILIPINAE Kovalev, 1979 synonymy by Ronquist (1999)
PROANACHARITINAE Kovalev, 1979 synonymy by Ronquist (1999)

##### Notes

Synonymy follows Fergusson (1986) except where otherwise noted.

#### Aegilips

Haliday in Walker, 1835

#### Aegilips atricornis

Fergusson, 1985

##### Distribution

England, Ireland

##### Notes

added by Fergusson (1985)

#### Aegilips nitidula

(Dalman, 1823)

Cynips nitidula Dalman, 1823
fumipennis (Westwood, 1833, *Anacharis*)
rufipes (Westwood, 1833, *Anacharis*)
dalmani Reinhard, 1860
rugicollis Reinhard, 1860
ruficornis Cameron, 1883
striolata Cameron, 1883
bicolorata Cameron, 1887

##### Distribution

England, Ireland, Isle of Man

#### Aegilips romseyensis

Fergusson, 1985

##### Distribution

England

##### Notes

added by Fergusson (1985)

#### Aegilips vena

Fergusson, 1985

##### Distribution

England, Scotland

##### Notes

added by Fergusson (1985)

#### Anacharis

Dalman, 1823

MEGAPELMUS Hartig, 1840
SYNAPSIS Förster, 1869 preocc.
PROSYNAPSIS Dalle Torre & Kieffer, 1910

#### Anacharis eucharoides

(Dalman, 1818)

tinctus Walker, 1835
typica Walker, 1835
petiolata (Zetterstedt, 1838, *Cynips*)
spheciformis (Hartig, 1840, *Megapelmus*)
eucharioides misspelling

##### Distribution

England, Ireland

#### Anacharis immunis

Walker, 1835

ensifer Walker, 1835
rufiventris (Hartig, 1841, *Megapelmus*)
staegeri Dahlbom, 1842
aquisgranensis (Förster, 1869, *Synapsis*)

##### Distribution

England, Ireland

#### Xyalaspis

Hartig, 1843

CONASPICERA Hedicke, 1914

##### Notes

Western Palaearctic species were revised by Mata-Casanova et al. (2015).

#### Xyalaspis armata

(Giraud, 1860)

Aegilips armatus Giraud, 1860
abietina (Giraud, 1860, *Aegilips*)
scotica (Cameron, 1883, *Aegilips*)

##### Distribution

England, Scotland, Ireland

#### Xyalaspis petiolata

Kieffer, 1901

subulifera misident.

##### Distribution

England, Scotland, Ireland

#### Aspicerinae

Dalla Torre & Kieffer, 1910

ONYCHIINAE Thomson, 1862

#### Aspicera

Dahlbom, 1842

ONYCHIA Haliday in Curtis, 1829 preocc.
BELLONA Giraud, 1860

#### Aspicera scutellata

(Villers, 1789)

Tenthredo scutellata Villers, 1789
ediogaster (Rossi, 1790, *Evania*)
bicolor (Boyer de Fonscolombe, 1832, *Figites*)
aculeator (Boyer de Fonscolombe, 1832, *Figites*)
ruficollis Kieffer, 1907 (*Aspicera scutellata* var.)

##### Distribution

Ireland

##### Notes

After the taxonomic changes in the revision by Ros-Farré and Pujade-Villar (2013) it is not entirely certain which nominal species is present in the British material. The authors cite *scutellata* as British but with a reference to Fergusson (1986), thus not based on reexamination of specimens.

#### Callaspidia

Dahlbom, 1842

##### Notes

Nomenclature follows Ros-Farré and Pujade-Villar (2009).

#### Callaspidia defonscolombei

Dahlbom, 1842

westwoodi Dahlbom, 1842
nigripes (Cameron, 1879, *Onychia*)
dufouri Giraud, 1860
fonscolombei Dahlbom, 1856 unjustified emendation
provancheri Ashmead, 1887
striolata (Cameron, 1888, *Onychia*)
areolata (Kieffer, 1901, *Onychia*)
rubricus Dettmer, 1924
vitripennis (Kieffer, 1901, *Onychia dufouri* var.)
minima (Kieffer, 1901, *Onychia fonscolombei* var.)

##### Distribution

England, Ireland

#### Melanips

Haliday in Walker, 1835

SCYTODES Hartig, 1840 preocc.
AMBLYNOTUS Hartig, 1843
ANOLYTUS Förster, 1869

##### Notes

Transferred from Figitinae to Aspicerinae by Buffington et al. (2007).

Synonymy follows Fergusson (1986).

#### Melanips alienus

Giraud, 1860

opacus misident.
dalmanni (Dahlbom, 1842, *Figites*) nom. nud.
longitarsus (Reinhard, 1860, *Amblynotus*)

##### Distribution

England, Ireland

#### Melanips microcerus

(Kieffer, 1903)

Amblynotus microcerus Kieffer, 1903

##### Distribution

England, Ireland

#### Melanips opacus

(Hartig, 1840)

Scytodes opacus Hartig, 1840
femoralis Cameron, 1883

##### Distribution

England, Ireland

#### Melanips sylvanus

Giraud, 1860

rufipes Förster, 1869
biusta (Cameron, 1879, *Omalaspis*)

##### Distribution

England, Scotland, Wales, Ireland

#### Omalaspis

Giraud, 1860

TAVARESIA Kieffer, 1901
LAMBERTONIA Kieffer, 1901

#### Omalaspis carinata

(Kieffer, 1901)

Tavaresia carinata Kieffer, 1901

##### Distribution

Wales

#### Charipinae

Dalla Torre & Kieffer, 1910

ALLOTRIINAE Thomson, 1862 unavailable
ALLOXYSTINAE Hellén, 1931
DILYTINI Kierych, 1979
LYTOXYSTINAE Kovalev, 1994

##### Notes

Except where noted, nomenclature follows Menke and Evenhuis (1991) and Ferrer-Suay et al. (2012d). Further taxonomic changes have been made in a series of papers by Ferrer-Suay and colleagues (Ferrer-Suay et al. 2012a, Ferrer-Suay et al. 2012b, Ferrer-Suay et al. 2012c, Ferrer-Suay et al. 2013a, Ferrer-Suay et al. 2013b, Ferrer-Suay et al. 2013c, Ferrer-Suay et al. 2014a, Ferrer-Suay et al. 2015, Ferrer-Suay et al. 2013d, Ferrer-Suay et al. 2014b). Tribal subdivisions have been abandoned following Paretas-Martínez et al. (2007), who found that Alloxystini was paraphyletic with respect to Charipini. Some Welsh occurrence records from Baker (2013).

#### Alloxysta

Förster, 1869

ALLOTRIA Westwood, 1833 preocc.
XYSTUS Hartig, 1840 preocc.
PEZOPHYCTA Förster, 1869
NEPHYCTA Förster, 1869
ADELIXYSTA Kierych, 1988
CARVERCHARIPS Kovalev, 1994

##### Notes

Quinlan (1978a) listed 36 *Alloxysta* species for Britain and Ireland, which Fergusson (1986) reduced to 11, mainly through synonymy. However, Fergusson did not work with much reared material and Evenhuis (1985) and Müller et al. (1999) have adopted much narrower species limits than Fergusson, an approach borne out by FVV’s work on the biology and taxonomy of the genus (see Van Veen et al. 2003).

species of *Alloxysta* excluded from the British and Irish list:

[*flavicornis* (Hartig, 1841, *Xystus*) incertae sedis]

[*ullrichi* (Giraud, 1860, *Allotria*) incertae sedis; *ullerichi* misspelling]

These species were listed by Quinlan (1978a) but not recognised as British or Irish by Fergusson (1986) and not found by FVV.

*Alloxysta* species of uncertain status:

[*ignorata* (Kieffer, 1900, *Dilyta*) nom. dub.] Listed by Fergusson (1986) as a synonym of *macrophadnus*, the type material of *ignorata* has not been located and it has not been possible to interpret the name (Ferrer-Suay et al. 2012b).

#### Alloxysta abdera

Fergusson, 1986

##### Distribution

England

##### Notes

added by Fergusson (1986)

#### Alloxysta apteroidea

Hellén, 1963

##### Distribution

England

##### Notes

Det. Van Veen, added here. The species identified as *brachyptera* by Müller et al. (1999) is actually *apteroidea*, although the true *brachyptera* has also been found in Silwood Park.

#### Alloxysta arcuata

(Kieffer, 1902)

Allotria arcuata Kieffer, 1902
minuta misident.
ligustri Evenhuis, 1976

##### Distribution

Scotland, Wales

##### Notes

Raised from synonymy with *brevis* by Ferrer-Suay et al. (2012a).

#### Alloxysta basimacula

(Cameron, 1886)

Allotria basimacula Cameron, 1886
caledonica (Cameron, 1886, *Allotria*)
perplexa (Cameron, 1889, *Allotria*)

##### Distribution

Scotland

##### Notes

Raised from synonymy with *macrophadnus* by Ferrer-Suay et al. (2013).

#### Alloxysta brachyptera

(Hartig, 1840)

Xystus brachypterus Hartig, 1840

##### Distribution

England, Scotland, Wales, Ireland

#### Alloxysta brevis

(Thomson, 1862)

Allotria brevis Thomson, 1862
minuta misident.

##### Distribution

England

#### Alloxysta castanea

(Hartig, 1841)

Xystus castaneus Hartig, 1841
melanogaster misident.
maculicollis (Cameron, 1886, *Allotria*)
ruficollis (Cameron, 1883, *Allotria*)
ruficeps (Cameron, 1883, *Allotria*) preocc.
megaptera (Cameron, 1889, *Allotria*)
dubia Kieffer, 1902 (*Alloxysta erythrothorax* var.)
rubriceps (Kieffer, 1902, *Allotria*)
semiclausa Kieffer, 1904
pruni (Hedicke, 1928, *Charips*)
defecta (Hartig, 1841, *Xystus*)
nigriventris (Thomson, 1862, *Allotria*)

##### Distribution

England, Wales

##### Notes

Synonymised with *fulviceps* by Fergusson (1986) but treated as a valid species by Ferrer-Suay et al. (2012b) following the advice of FVV. *Allotria ruficollis* is listed as a synonym of *fulviceps* in Fauna Europaea (Noyes et al. 2004), following alternate synonymisations under *erythrothorax* (Quinlan 1974) and *castanea* (Evenhuis 1982). In Müller et al. (1999) this was recorded as ‘*Alloxysta* f1’.

#### Alloxysta circumscripta

(Hartig, 1841)

Xystus circumscriptus Hartig, 1841

##### Distribution

England

##### Notes

Synonymised under *victrix* by Fergusson (1986) but this has not been supported by other workers (Menke and Evenhuis 1991, Van Veen et al. 2003).

#### Alloxysta citripes

(Thomson, 1862)

Allotria citripes Thomson, 1862
britannica Kieffer, 1902 (*Alloxysta citripes* var.)
albipes (Kieffer, 1904, *Allotria*)
brevicella Belizin, 1966 synonymy by Ferrer-Suay et al. (2012b)

##### Distribution

England, Wales

#### Alloxysta consobrina

(Zetterstedt, 1838)

Cynips consobrina Zetterstedt, 1838
fuscicornis (Hartig, 1841, *Xystus*) synonymy by Ferrer-Suay et al. (2013b)
ancylocera (Cameron, 1886, *Allotria*)
brassicae (Ashmead, 1887, *Allotria*)
infuscata (Kieffer, 1902, *Allotria*)
aphidae (Froggatt, 1904, *Hypodiranchis*)
grioti (De Santis, 1937, *Charips*)

##### Distribution

England

##### Notes

Synonymised under *victrix* by Fergusson (1986) but raised from synonymy by Menke and Evenhuis (1991), a result supported by Van Veen et al. (2003).

#### Alloxysta crassa

(Cameron, 1889)

Allotria crassa Cameron, 1889

##### Distribution

Scotland

##### Notes

Raised from synonymy with *macrophadnus* by Ferrer-Suay et al. (2013).

#### Alloxysta cursor

(Hartig, 1840)

Xystus cursor Hartig, 1840
castanea (Kieffer, 1904, *Pezophycta*) preocc.

##### Distribution

England

##### Notes

Considered a nomen dubium in Ferrer-Suay et al. (2014) but Evenhuis (1982) studied the type and subsequently identified British specimens as belonging to this taxon, so we see no problem with considering it a valid British species. It was listed as *castanea* in Quinlan (1978a) but not mentioned by Fergusson (1986) and then recorded again by Müller et al. (1999).

#### Alloxysta erythrothorax

(Hartig, 1840)

Xystus erythrothorax Hartig, 1840
trapezoidea misident.
defecta (Hartig, 1841, *Xystus*)
nigriventris (Thomson, 1862, *Allotria*)

##### Distribution

England, Scotland

##### Notes

Removed from synonymy with *fulviceps* by Pujade-Villar et al. (2011).

#### Alloxysta halterata

(Thomson, 1862)

Allotria halterata Thomson, 1862

##### Distribution

England

##### Notes

Synonymised under *pedestris* by Fergusson (1986) but considered here to be a valid species (Ferrer-Suay et al. 2012b).

#### Alloxysta leunisii

(Hartig, 1841)

Xystus leunisii Hartig, 1841

##### Distribution

England, Wales

##### Notes

added by Van Veen et al. (2003)

#### Alloxysta macrophadnus

(Hartig, 1841)

Xystus macrophadnus Hartig, 1841
testacea misident.
aptera misident.
brachyptera misident.
fuscipes misident.
nigriventris misident.
macrophadna misspelling
filicornis (Cameron, 1889, *Allotria*)
scutellata Kieffer, 1902
rubromaculata Kieffer, 1902 (*Alloxysta nigriventris* var.)

##### Distribution

England, Scotland, Wales

##### Notes

*Alloxysta testeacea* is recorded as a misidentification of *macrophadna* by Fergusson (1986) but erroneously listed as a valid species, occurring in Britain, in Fauna Europaea (Noyes et al. 2004), but with the comment that the species might be synonymous with *pleuralis*.

#### Alloxysta marshalliana

(Kieffer, 1900)

Nephycta marshalliana Kieffer, 1900

##### Distribution

Scotland

##### Notes

Synonymised under *macrophadna* by Fergusson (1986) but considered here to be a valid species (Ferrer-Suay et al. 2012b, following pers. comm. by FVV).

#### Alloxysta mullensis

(Cameron, 1883)

Allotria mullensis Cameron, 1883

##### Distribution

Scotland

##### Notes

Raised from synonymy with *brevis* by Ferrer-Suay et al. (2012a).

#### Alloxysta nigrita

(Thomson, 1862)

Allotria nigrita Thomson, 1862

##### Distribution

Wales

##### Notes

added by Baker (2013)

#### Alloxysta obscurata

(Hartig, 1840)

Xystus obscuratus Hartig, 1840
homotoma Kieffer, 1904 (*Alloxysta ullrichi* var.)

##### Distribution

England

##### Notes

det. Van Veen, added here.

#### Alloxysta pallidicornis

(Curtis, 1838)

Cynips pallidicornis Curtis, 1838
minuta (Zetterstedt, 1838, *Cynips*)
forticornis (Giraud, 1860, *Allotria*)
basalis (Thomson, 1862, *Allotria*)
anthracina Andrews, 1978

#### Alloxysta pedestris

(Curtis, 1838)

Cynips pedestris Curtis, 1838

##### Distribution

England, Wales, Ireland

#### Alloxysta piceomaculata

(Cameron, 1883)

Allotria piceomaculata Cameron, 1883

##### Distribution

Scotland, Wales

##### Notes

Raised from synonymy with *macrophadnus* by Ferrer-Suay et al. (2013).

#### Alloxysta pilipennis

(Hartig, 1840)

Xystus pilipennis Hartig, 1840
flavicornis (Hartig, 1841, *Xystus*) synonymy by Ferrer-Suay et al. (2014)

##### Distribution

England

##### Notes

J. Pujade-Villar (pers. comm.) has seen English material.

#### Alloxysta pleuralis

(Cameron, 1879)

Allotria pleuralis Cameron, 1879
unicolor (Kieffer, 1902, *Alloxysta pusilla* var.)
gautieri Kieffer, 1922

##### Distribution

England, Scotland, Ireland

#### Alloxysta pusilla

(Kieffer, 1902)

Allotria pusilla Kieffer, 1902

##### Distribution

England

##### Notes

added by Sanders and Van Veen (2010)

#### Alloxysta ramulifera

(Thomson, 1862)

Allotria ramulifera Thomson, 1862
minuta (Hartig, 1840, *Xystus*)
discreta (Förster, 1869, *Nephycta*)
parvicellula (Kieffer, 1904, *Allotria*)

##### Distribution

England

##### Notes

added by Müller et al. (1999)

#### Alloxysta semiaperta

Fergusson, 1986

##### Distribution

England, Ireland

##### Notes

added by Fergusson (1986)

#### Alloxysta tscheki

(Giraud, 1860)

Allotria tscheki Giraud, 1860

##### Distribution

England

##### Notes

Excluded from the British and Irish list by Fergusson (1986) but reinstated by Van Veen et al. (2003).

#### Alloxysta victrix

(Westwood, 1833)

Allotria victrix Westwood, 1833
fulviceps (Curtis, 1838, *Cynips*)
ruficeps (Zetterstedt, 1838, *Cynips*)
erythrocephalus (Hartig, 1840, *Xystus*)
tritici (Fitch, 1861, *Allotria*)
macrocera (Thomson, 1877, *Allotria*)
amygdali (Buckton, 1879, *Cynips*) nom. nud.
atriceps (Buckton, 1879, *Cynips*)
curvicornis (Cameron, 1883, *Allotria*)
lateralis (Kieffer, 1902, *Allotria luteicornis* var.)
luteiceps (Kieffer, 1902, *Allotria victrix* var.)
luteicornis (Kieffer, 1902, *Allotria*)
areolata (Kieffer, 1909, *Charips*)
grandicornis (Kieffer, 1904, *Allotria*)
io (Girault, 1932 *Sarorthrus*)

##### Distribution

England, Scotland, Wales

#### Apocharips

Fergusson, 1986

#### Apocharips trapezoidea

(Hartig, 1841)

Xystus trapezoideus Hartig, 1841
xanthocephala (Thomson, 1862, *Allotria*)

##### Distribution

England, Wales

#### Dilyta

Förster, 1869

CHARIPS Haliday in Marshall, 1870
GLYPTOXYSTA Thomson, 1877
DYLITA misspelling

#### Dilyta subclavata

Förster, 1869

microcera (Haliday, 1870, *Charips*)
heterocera (Thomson, 1877, *Glyptoxysta*)
talitzkii (Belizin, 1966, *Glyptoxysta*) synonymy by Paretas-Martínez et al. (2011)

##### Distribution

England, Scotland, Wales

#### Phaenoglyphis

Förster, 1869

HEMICRISIS Förster, 1869
AULOXYSTA Thomson, 1877
BOTHRIOXYSTA Kieffer, 1902
CHARIPSELLA Bréthes, 1913

##### Notes

Fergusson (1986) established *Hemicrisis* (including only *ruficornis*) as a synonym of *Phaenoglyphis*, it was then raised from synonymy by Ronquist (1999) only to be synonymised again with *Phaenoglyphis* by Pujade-Villar and Paretas-Martínez (2006).

#### Phaenoglyphis dolichocera

(Cameron, 1889)

Allotria dolichocera Cameron, 1889

##### Distribution

England, Ireland

#### Phaenoglyphis longicornis

(Hartig, 1840)

Xystus longicornis Hartig, 1840

##### Distribution

Wales

##### Notes

Listed by Quinlan (1978a) as a species of *Alloxysta*, not mentioned by Fergusson (1986), then recorded as British by Baker (2013) based on specimens identified by M. Ferrer-Suay.

#### Phaenoglyphis ruficornis

(Förster, 1869)

Hemicrisis ruficornis Förster, 1869

##### Distribution

England, Wales

##### Notes

*Phaenoglyphis pubicollis* (Thomson, 1877, *Allotria*) was resurrected as a valid species by Pujade-Villar and Paretas-Martínez (2006). The authors did not examine material from a wide range but state that *pubicollis* ‘is only represented by type material’, implying that the real *ruficornis* is the widespread one.

#### Phaenoglyphis salicis

(Cameron, 1883)

Allotria salicis Cameron, 1883
forticornis Cameron, 1888

##### Distribution

England, Wales

#### Phaenoglyphis villosa

(Hartig, 1841)

Xystus villosus Hartig, 1841
piciceps (Thomson, 1862, *Allotria*)
collina (Cameron, 1889, *Allotria*)
ambrosiae (Ashmead, 1898, *Allotria*)
carpentieri (Kieffer, 1902, *Allotria*)
foveigera (Kieffer, 1902, *Allotria*)
curvata (Kieffer, 1902, *Allotria*)
recticornis (Kieffer, 1902, *Allotria*)
subaptera (Kieffer, 1904, *Alloxysta*)
campyla (Kieffer, 1904, *Alloxysta*)
necans (Kieffer, 1909, *Glyptoxysta*)
numidica (Kieffer, 1909, *Bothrioxysta*)
bifoveata (Girault, 1931, *Glyptoxysta*)
islandica (Hellén, 1931, *Alloxysta*)
flavipes (Ionescu, 1963 *Charips*)

##### Distribution

England, Wales

#### Phaenoglyphis xanthochroa

Förster, 1869

rufa (Thomson, 1877, *Allotria*)
obfuscata Kieffer, 1901

##### Distribution

England

#### Eucoilinae

Thomson, 1862

##### Notes

Eucoilinae has been a subfamily of Figitidae rather than a family of its own since Ronquist (1999). Tribal classification follows Forshage and Nordlander (2008). Synomymy and distribution data taken from Quinlan (1978b) and Nordlander (1978), Nordlander (1980), Nordlander (1981), Forshage and Nordlander (2008), supplemented by Fauna Europaea (Noyes et al. 2004) and MF’s identifications of material in BMNH. Some Irish records from O'Connor (2004).

#### Diglyphosematini

Belizin, 1961

#### Diglyphosema

Förster, 1869

#### Diglyphosema conjungens

Kieffer, 1904

##### Distribution

England, Wales, Ireland

#### Disorygma

Förster, 1869

ECTOLYTA Förster, 1869
ERISPHAGIA Förster, 1869

#### Disorygma curtum

(Giraud, 1860)

Eucoila curta Giraud, 1860

##### Distribution

England

#### Disorygma depile

(Giraud, 1860)

Eucoila depilis Giraud, 1860
incrassata (Thomson, 1862, *Cothonaspis*)
divulgata Förster, 1869

##### Distribution

England, Ireland

#### Microstilba

Förster, 1869

#### Microstilba striolata

Kieffer, 1901

heterogena (Giraud, 1860, *Eucoila*) *auctt., sensu* Cameron

##### Distribution

Scotland

##### Notes

Supposed English specimens in BMNH are all *Disorygma curtum* (det. MF).

#### Eucoilini

Thomson, 1862

#### Eucoila

Westwood, 1833

EUCOELA Agassiz, 1846
LYTOSEMA Kieffer, 1901
PSILODORA Förster, 1869

#### Eucoila crassinerva

Westwood, 1833

boyenii (Hartig, 1840, *Cothonaspis*)
intermedia (Kieffer, 1901, *Psilodora*)

##### Distribution

England, Scotland, Ireland

#### Eucoila maculata

(Hartig, 1840, Cothonaspis)

Cothonaspis maculatus Hartig, 1840
guerini Dahlbom, 1842
brevialata Belizin, 1973

##### Distribution

England, Scotland

#### Leptopilina

Förster, 1869

##### Notes

Synonymy from Nordlander (1980).

#### Leptopilina clavipes

(Hartig, 1841)

Cothonaspis clavipes Hartig, 1841

##### Notes

BMNH, det. Forshage, added here.

#### Leptopilina fimbriata

(Kieffer, 1901)

Eucoela fimbriata Kieffer, 1901
xanthoneura nec (Förster, 1869, *Episoda*) *sensu*Quinlan (1978b)
longipes (Cameron, 1883, *Erisphagia*) preocc.
xanthopum (Kieffer, 1904, *Psilosema*)
filicorne (Kieffer, 1904, *Psilosema*)
longicorne (Kieffer, 1907, *Psilosema*)
dolichocera (Hellén, 1960, *Episoda*)

##### Distribution

England

##### Notes

Treated as *Episoda xanthoneura* by Quinlan (1978b).

#### Leptopilina heterotoma

(Thomson, 1862)

Eucoila heterotoma Thomson, 1862
? musti (Rondani, 1875, *Xystus*)
monilicornis (Kieffer, 1904, *Ganaspis*)
subnuda (Kieffer, 1904, *Ganaspis*)
philippinensis (Kieffer, 1916, *Erisphagia*)
bochei (Weld, 1944, *Pseudeucoila*)

##### Distribution

England, Ireland

##### Notes

Treated as *Ganaspis subnuda* by Quinlan (1978b).

#### Leptopilina longipes

(Hartig, 1841)

Cothonaspis longipes Hartig, 1841

##### Distribution

England

##### Notes

BMNH, det. Forshage, added here.

#### Trybliographa

Förster, 1869

EPISODA Förster, 1869 synonymy by Nordlander (1980)
IDIOMORPHA Förster, 1869
HYPOLETHRIA Förster, 1869
PSICHACRA Förster, 1869
ADIERIS Förster, 1869
PIEZOBRIA Förster, 1869
PILINOTHRIX Förster, 1869
ANECTOCLIS Förster, 1869
Trybliographa *COTHONASPIS auctt*. *nec* Hartig, 1840
DIMICROSTROPHIS Ashmead, 1886
DUSMETIOLA Tavares, 1924
Trybliographa *EUCOILA auctt*. *nec* Westwood, 1835
PSEUDEUCOILA Ashmead, 1903

##### Notes

Synonymy mostly from Nordlander (1981). Several new species of *Trybliographa* are present in Britain and several new synonymies will result from MF’s unpublished revision. Some changes have been made in anticipation of this publication as they affect names described from British specimens.

species of *Trybliographa* excluded from the British and Irish list:

[*testaceipes* Cameron, 1883] This name will be placed in synonymy.

#### Trybliographa agaricola

(Thomson, 1862)

Eucoila agaricola Thomson, 1862

##### Distribution

England

##### Notes

BMNH, det. Forshage, added here.

#### Trybliographa albipennis

(Thomson, 1861)

Eucoila albipennis Thomson, 1861
spaniandra Kerrich & Quinlan, 1960

##### Distribution

England

#### Trybliographa atra

(Hartig, 1840)

Cothonaspis ater Hartig, 1840
nigricornis Cameron, 1883

##### Distribution

England, Scotland, Ireland

#### Trybliographa cubitalis

(Hartig, 1841)

Cothonaspis cubitalis Hartig, 1841

##### Distribution

England, Ireland

#### Trybliographa diaphana

(Hartig, 1841)

Cothonaspis diaphana Hartig, 1841

##### Distribution

England, Scotland, Wales, Ireland

#### Trybliographa erythrocera

(Thomson, 1877)

Eucoila erythrocera Thomson, 1877

##### Distribution

England

##### Notes

BMNH, det. Forshage, added here. Generic combination not yet formally published.

#### Trybliographa fovealis

(Thomson, 1862)

Eucoila fovealis Thomson, 1862

##### Distribution

England

##### Notes

BMNH, det. Forshage, added here.

#### Trybliographa glottiana

(Cameron, 1883)

Psichacra glottiana Cameron, 1883
proxima (Cameron, 1889, *Eucoila*)
agaricorum (Kieffer, 1902, *Eucoela*)

##### Distribution

England, Scotland

#### Trybliographa gracilicornis

(Cameron, 1888)

Eucoila gracilicornis Cameron, 1888

##### Distribution

England, Scotland, Wales, Ireland

#### Trybliographa longicornis

(Hartig, 1840)

Cothonaspis longicornis Hartig, 1840
gracilis (Dahlbom, 1846, *Eucoila*)
subspinosa (Kieffer, 1904, *Cothonaspis*)

##### Distribution

England, Ireland

#### Trybliographa mandibularis

(Zetterstedt, 1838)

Figites mandibularis Zetterstedt, 1838
similis (Cameron, 1883, *Psichacra*)

##### Distribution

England, Scotland

#### Trybliographa nigripes

(Giraud, 1860)

Eucoila nigripes Giraud, 1860

##### Distribution

England

##### Notes

BMNH, det. Forshage, added here.

#### Trybliographa rapae

(Westwood, 1835)

Eucoila rapae Westwood, 1835
coronata (Hartig, 1841, *Cothonaspis*)
insignis (Giraud, 1860, *Eucoila*)
octotoma (Thomson, 1862, *Eucoila*)
scutellaris Förster, 1869
scutellaris nec (Latreille, 1805, *Figites*) *sensu* Hartig, 1840
melanocera (Förster, 1869, *Idiomorpha*)
crassicornis Cameron, 1889
fortinervis (Cameron, 1889, *Eucoila*)
ventralis (Kieffer, 1901, *Eucoela*)
ruficornis (Kieffer, 1902, *Eucoela*)
erythrocera nec (Thomson, 1862, *Eucoela*) *sensu* Cameron, 1890
britannica (Kieffer, 1905, *Eucoela*)

##### Distribution

England, Scotland, Wales, Ireland

#### Trybliographa rufula

(Förster, 1855)

Eucoila rufula Förster, 1855
dalei (Cameron, 1879, *Psichacra*)

##### Distribution

England, Ireland

#### Trybliographa scotica

(Cameron, 1889)

Eucoila scotica Cameron, 1889

##### Distribution

England, Scotland

##### Notes

Recently removed from synonymy with *trichopsila* in Forshage et al. (2013).

#### Trybliographa strandi

(Hedicke, 1914)

Cothonaspis strandi Hedicke, 1914

##### Distribution

England

##### Notes

BMNH, det. Forshage, added here.

#### Trybliographa subnebulosa

(Giraud, 1860)

Eucoila subnebulosa Giraud, 1860

##### Distribution

England

##### Notes

BMNH, det. Forshage, added here.

#### Trybliographa trichopsila

(Hartig, 1841)

Cothonaspis trichopsilus Hartig, 1841
brachytricha (Kieffer, 1901, *Eucoela*)
claripennis (Thomson, 1862, *Eucoila*)

##### Distribution

England

#### Ganaspini

Belizin, 1961

#### Chrestosema

Förster, 1869

RECENTIA Belizin, 1961

##### Notes

species of *Chrestosema* excluded from the British and Irish list:

[*antennale* Kieffer, 1904; England, Ireland] This name will be placed in synonymy

#### Chrestosema erythropum

Förster, 1869

##### Distribution

England

##### Notes

BMNH, det. Forshage, added here.

#### Didyctium

Riley, 1879

HEPTAMEROCERA Ashmead, 1896

#### Didyctium nigriclava

(Kieffer, 1904)

Cothanaspis nigriclava Kieffer, 1904

##### Distribution

England, Wales

##### Notes

BMNH, det. Forshage, added here.

#### Ganaspis

Förster, 1869

#### Ganaspis mundata

Förster, 1869

tenuicornis Kieffer, 1904

##### Distribution

England

##### Notes

BMNH, det. Forshage, added here.

#### Ganaspis seticornis

(Hellén, 1960)

Episoda seticornis Hellén, 1960
ciliaria (Belizin, 1968, *Odonteucoila*)

##### Distribution

England, Scotland

##### Notes

BMNH, det. Forshage, added here.

#### Glauraspidia

Thomson, 1861

AGLAOTOMA Förster, 1869
APHISTOPHYZA Förster, 1869
DIRANCHIS Förster, 1869
CRYPTEUCOELA Kieffer, 1904
AGLAOTOMIDEA Rohwer & Fagan, 1917

#### Glauraspidia microptera

(Hartig, 1840)

Cothonaspis micropterus Hartig, 1840
codrina (Hartig, 1841, *Cothonaspis*)
subtilis (Dahlbom, 1842, *Eucoela*)
carpentieri Kieffer, 1901
giraudi (Kieffer, 1902, *Aglaotoma*)
foersteri (Kieffer, 1904, *Aglaotoma*)
giraudi (Kieffer, 1904, *Crypteucoela*) preocc.
elegans Ionescu, 1963

##### Distribution

England, Wales, Ireland

#### Hexacola

Förster, 1869

HEXAPLASTA Förster, 1869

#### Hexacola hexatoma

(Hartig, 1841)

Cothonaspis hexatoma Hartig, 1841
picicrus (Giraud, 1860, *Eucoila*)
fuscipes (Mayer, 1923, *Cothonaspis*)

##### Distribution

England

#### Mirandicola

Belizin, 1968

PSEUDOPSICHACRA Quinlan, 1975

#### Mirandicola sericea

(Thomson, 1877)

Glauraspidia sericea Thomson, 1877
bispinosa (Kieffer, 1901, *Eucoela*)
sauteri (Hedicke, 1913, *Psichacra*)

##### Distribution

England, Ireland

#### Kleidotomini

Hellén, 1960

#### Cothonaspis

Hartig, 1840

PSILOSEMA Kieffer, 1901

#### Cothonaspis gracilis

Hartig, 1841

giraudi (Dalla Torre & Kieffer, 1910, *Erisphagia*)

##### Distribution

England

#### Cothonaspis longula

Nordlander, 1976

##### Distribution

England

#### Cothonaspis pentatoma

Hartig, 1841

##### Distribution

England, Scotland

#### Eutrias

Förster, 1869

#### Eutrias tritoma

(Thomson, 1877)

Eucoila tritoma Thomson, 1877

##### Distribution

England

#### Kleidotoma

Westwood, 1833

APHYOPTERA Förster, 1869
APHILOPTERA Förster, 1869
AGROSCOPA Förster, 1869
HEPTAMERIS Förster, 1869
NEDINOPTERA Förster, 1869
RHYNCHACIS Förster, 1869
PENTACRITA Förster, 1869
TETRAHOPTRA Förster, 1869
TETRATOMA Cameron, 1890
ARHOPTRA Kieffer, 1901
PENTARHOPTRA Kieffer, 1901
SCHIZOSEMA Kieffer, 1901
KLEIDOTOMIDEA Rohwer & Fagan, 1917
PENTAKLEIDOTA Weld, 1951

##### Notes

Subgeneric classification was finally abandoned and all subgeneric names considered mere synonyms in Forshage and Nordlander (2008).

Species of *Kleidotoma* removed from the British and Irish list:

[*myrmecophila* Kieffer, 1908] Recorded from Britain in Fauna Europaea (Noyes et al. 2004) but in error.

#### Kleidotoma affinis

Cameron, 1889

##### Distribution

Scotland

#### Kleidotoma caledonica

Cameron, 1888

##### Distribution

England, Scotland, Ireland

#### Kleidotoma ciliaris

(Zetterstedt, 1838)

Figites ciliaris Zetterstedt, 1838
melanopoda Cameron, 1888

##### Distribution

England, Scotland

##### Notes

Usually regarded as a species of *Trybliographa* but published as a new combination in *Kleidotoma* by Jonsell et al. (1999) in an ecological paper and listed as such in Fauna Europaea (Noyes et al. 2004). The *ciliaris sensu auctt. nec* Zetterstedt, refers to various small *Trybliographa* spp.

#### Kleidotoma dolichocera

Thomson, 1877

carpentieri (Kieffer, 1904, *Cleidotoma*)

##### Distribution

England, Wales, Ireland

#### Kleidotoma elegans

Cameron, 1889

##### Distribution

England, Scotland

#### Kleidotoma filicornis

Cameron, 1889

##### Distribution

England

#### Kleidotoma gracilicornis

Cameron, 1889

##### Distribution

England

#### Kleidotoma gryphus

Thomson, 1861

#### Kleidotoma halophila

Thomson, 1861

##### Distribution

England

#### Kleidotoma hexatoma

Thomson, 1862

##### Distribution

England, Scotland

#### Kleidotoma longicornis

Cameron, 1889

##### Distribution

England, Scotland

#### Kleidotoma longipennis

Cameron, 1889

##### Distribution

England, Scotland, Ireland

#### Kleidotoma marshalli

Cameron, 1889

antecella Belizin, 1964

##### Distribution

England, Ireland

#### Kleidotoma nigra

(Hartig, 1840)

Cothonaspis niger Hartig, 1840
crassiclava Cameron, 1888
nigripes Cameron, 1888
brevicornis (Kieffer, 1904, *Rhynchasis*)
tetramora (Kieffer, 1904, *Rhynchasis*)

##### Distribution

England, Scotland, Ireland

#### Kleidotoma pentatoma

Thomson, 1861

albipennis Cameron, 1886 *nec* Thomson, 1861

##### Distribution

England, Scotland, Ireland

#### Kleidotoma picipes

Cameron, 1886

##### Distribution

Scotland, Ireland

#### Kleidotoma psiloides

Westwood, 1833

##### Distribution

England, Wales

#### Kleidotoma pygmea

(Dahlbom, 1842)

Eucoila pygmea Dahlbom, 1842

##### Distribution

England, Ireland

#### Kleidotoma striata

Cameron, 1886

##### Distribution

England, Scotland

#### Kleidotoma striaticollis

Cameron, 1880

##### Distribution

England, Scotland

#### Kleidotoma subaptera

(Walker, 1834)

Figites subaptera Walker, 1834
helgolandica (Förster, 1869, *Agroscopa*)

##### Distribution

England, Wales, Ireland

#### Kleidotoma thomsoni

Forshage, sp. nov., replacement name

tetratoma Thomson, 1861 preocc. Name preoccupied by *Kleidotoma tetratoma* (Hartig, 1841, *Cothonaspis*).

##### Distribution

England, Scotland, Ireland

#### Kleidotoma tomentosa

(Giraud, 1860)

Eucoila tomentosa Giraud, 1860
anisomera (Förster, 1869, *Aphiloptera*)
erythropa Thomson, 1877

##### Distribution

England

#### Kleidotoma truncata

Cameron, 1889

##### Distribution

England, Scotland

#### Trichoplastini

Kovalev, 1989

#### Rhoptromeris

Förster, 1869

MIOMOERA Förster, 1869
HEXAMEROCERA Kieffer, 1901
STRIATELLIA Belizin, 1966

##### Notes

Synonymy taken from Nordlander (1978) .

Species of *Rhoptromeris* removed from the British and Irish list:

[*nigriventris* Nordlander, 1978] *Rhoptromeris nigriventris* was listed by Quinlan (1978b) but this was most likely a mistake. Nordlander (1978) mentions that some Swedish paratypes were deposited in BMNH. There are no British or Irish specimens identified as *nigriventris* in the collection.

#### Rhoptromeris heptoma

(Hartig, 1840)

Cothonaspis heptomus Hartig, 1840
biscapus (Hartig, 1840, *Cothonaspis*)
eucera (Hartig, 1841, *Cothonaspis*)
nodosa (Giraud, 1860, *Eucoila*)
parvula (Thomson, 1862, *Eucoila*)
aberrans (Förster, 1869, *Miomoera*)
aequalis (Kieffer, 1901, *Eucoela*)
widhalmi Kurdjumov, 1912

##### Distribution

England, Scotland, Wales, Ireland

#### Rhoptromeris villosa

(Hartig, 1840)

Cothonaspis villosus Hartig, 1840

##### Distribution

England, Wales, Ireland

##### Notes

BMNH, det. Forshage, added here.

#### Trichoplasta

Benoit, 1956

#### Trichoplasta sp. indet.

##### Notes

BMNH, det. Forshage, added here. Only a single male specimen has been found amongst British material; it is not identifiable to species level with our current state of knowledge, and is listed here as a record of the genus from Britain.

#### Figitinae

Hartig, 1840

##### Notes

*Melanips* has been transferred to Aspicerinae by Buffington et al. (2007).

Synonymy follows Fergusson (1986) except where otherwise noted.

#### Amphithectus

Hartig, 1840

AMPHITECTUS misspelling

##### Notes

Removed from synonymy with *Sarothrus* by Ronquist (1999).

#### Amphithectus areolatus

(Hartig, 1840)

Sarothrus areolatus Hartig, 1840
dahlbomii Hartig, 1840
piceus (Dahlbom, 1842, *Figites*) nom. nud.
fumipennis (Giraud, 1860, *Melanips*)

##### Distribution

England

#### Figites

Latreille, 1802

PSILOGASTER Hartig, 1840
PYCNOTRICHIA Förster, 1869
OMALOSPOIDES Hedicke, 1913

##### Notes

Species of *Figites* excluded from the British and Irish list:

[*laevigatus* Dahlbom, 1842]

[*reinhardi* Kieffer, 1901]

[*urticarum* Dahlbom, 1842]

Included in Quinlan (1978a) but not by Fergusson (1986).

#### Figites anthomyiarum

Bouché, 1834

##### Distribution

England, Ireland

#### Figites consobrinus

Giraud, 1860

scutellaris misident.
dentiscuta Hellén, 1937

##### Distribution

England, Ireland

#### Figites ictus

Fergusson, 1986

##### Distribution

England, Ireland

##### Notes

added by Fergusson (1986)

#### Figites scutellaris

(Rossi, 1794)

Cynips scutellaris Rossi, 1794
ruficornis (Rossi, 1794, *Cynips*)
abbreviator (Herrich-Schäffer in Panzer, 1801, *Ophion*)
tibialis (Hartig, 1840, *Psilogaster*)
letzneri (Hedicke, 1913, *Omalaspoides*)

##### Distribution

England

#### Lonchidia

Thomson, 1862

#### Lonchidia clavicornis

Thomson, 1862

##### Distribution

England, Scotland, Ireland

#### Lonchidia maculipennis

(Dahlbom, 1842)

Figites maculipennis Dahlbom, 1842

##### Distribution

England, Ireland

#### Sarothrus

Hartig, 1840

#### Sarothrus tibialis

(Zetterstedt, 1838)

Cynips tibialis Zetterstedt, 1838
canaliculatus Hartig, 1840
silesiacus (Hedicke, 1913, *Omalaspoides*)

##### Distribution

England, Wales, Ireland

#### Xyalophora

Kieffer, 1901

#### Xyalophora clavata

(Giraud, 1860)

Figites clavatus Giraud, 1860

##### Distribution

Wales, Ireland

##### Notes

added by Fergusson (1986)

#### Zygosis

Förster, 1869

DICERAEA Förster, 1869
THYREOCERA Ashmead, 1887

#### Zygosis urticeti

(Dahlbom, 1842)

Figites urticeti Dahlbom, 1842
heteropterus (Hartig, 1843, *Psilogaster*)

##### Distribution

England, Ireland

### Ibaliidae

#### Ibaliidae

Thomson, 1862

#### Ibalia

Latreille, 1802

SAGARIS Panzer, 1806

#### Ibalia leucospoides

(Hockenwarth, 1785)

Ichneumon leucospoides Hockenwarth, 1785
cultellator (Fabricius, 1793, *Ichneumon*)
ensiger Norton, 1862
suprunenkoi Jacobson, 1899
gigantea Yoshimoto, 1970

##### Distribution

England

#### Ibalia rufipes

Cresson, 1879

drewseni Borries, 1891
shirmeri Dalla Torre & Kieffer, 1910

##### Distribution

England, Scotland

## Supplementary Material

Supplementary material 1 Checklist of the British and Irish Cynipoidea Data type: formatted text
Brief description: Word document version of the checklist
File: oo_124446.docx Forshage, M., Bowdrey, J.P., Spooner, B.M., Van Veen, F. & Broad, G.R.

Supplementary material 2 Checklist of British and Irish Cynipoidea Data type: spreadsheet
Brief description: Excel spreadsheet version of the checklist
File: oo_124447.xlsx Forshage, M., Bowdrey, J.P., Spooner, B.M., Van Veen, F. & Broad, G.R.

## Figures and Tables

**Figure 1a.**
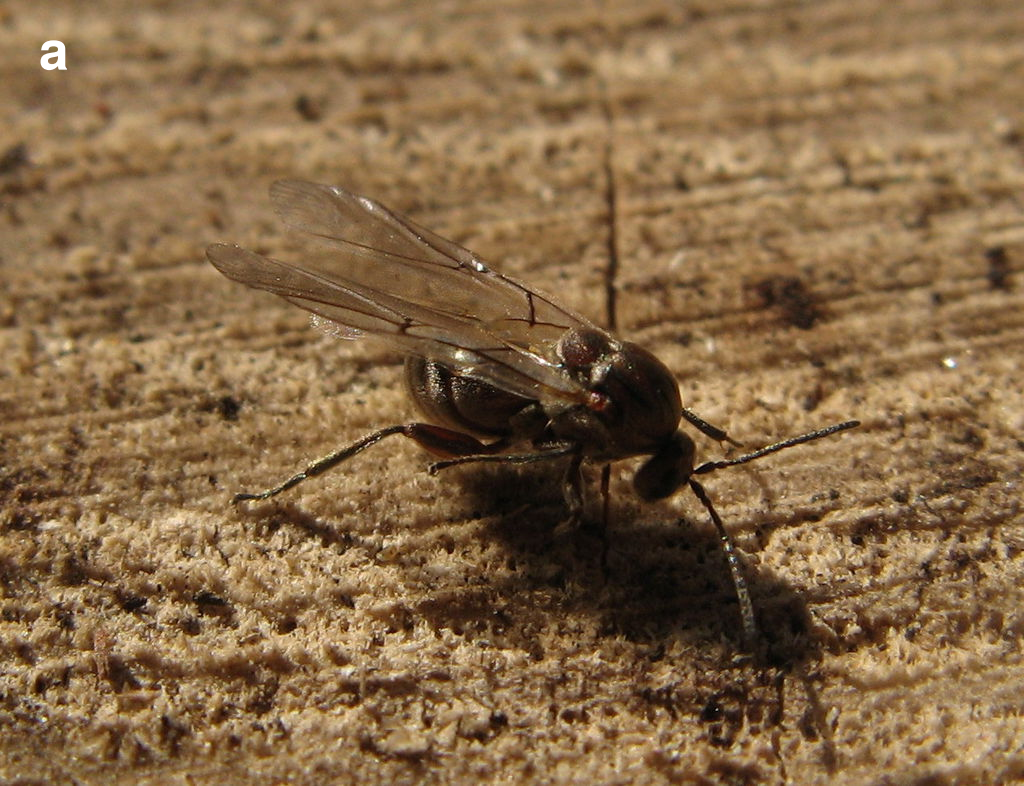
*Andricus quercuscorticis* (Linnaeus), agamic female (J.P. Bowdrey)

**Figure 1b.**
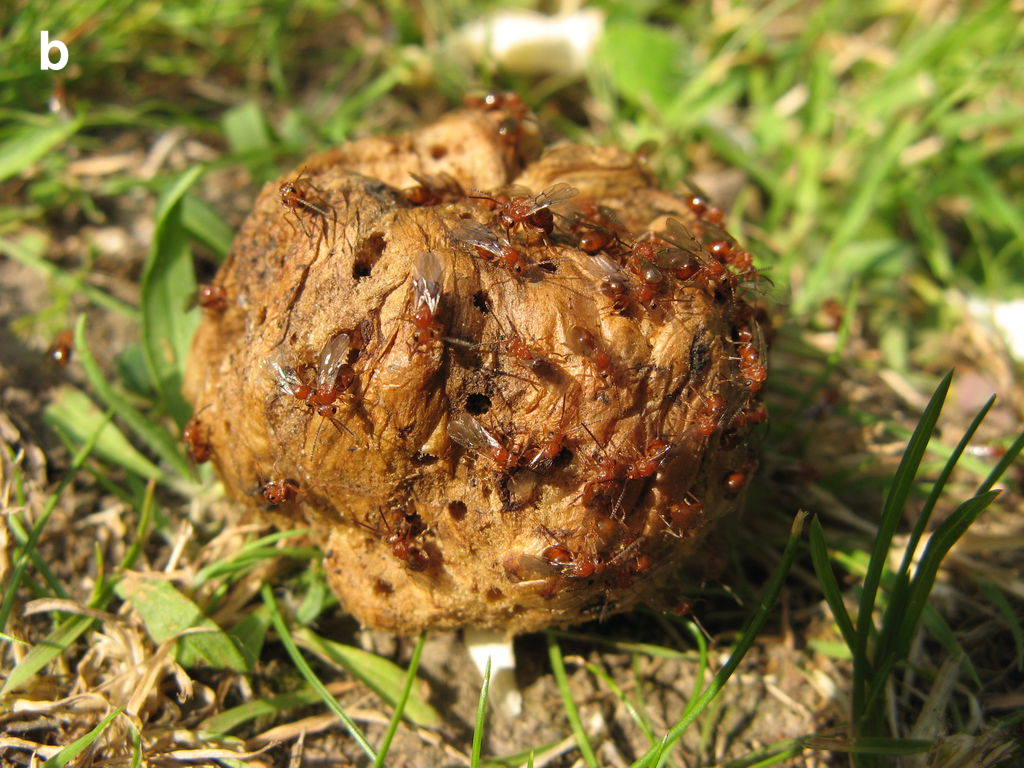
*Biorhiza pallida* (Olivier), sexual females on their gall (J.P. Bowdrey)

**Figure 1c.**
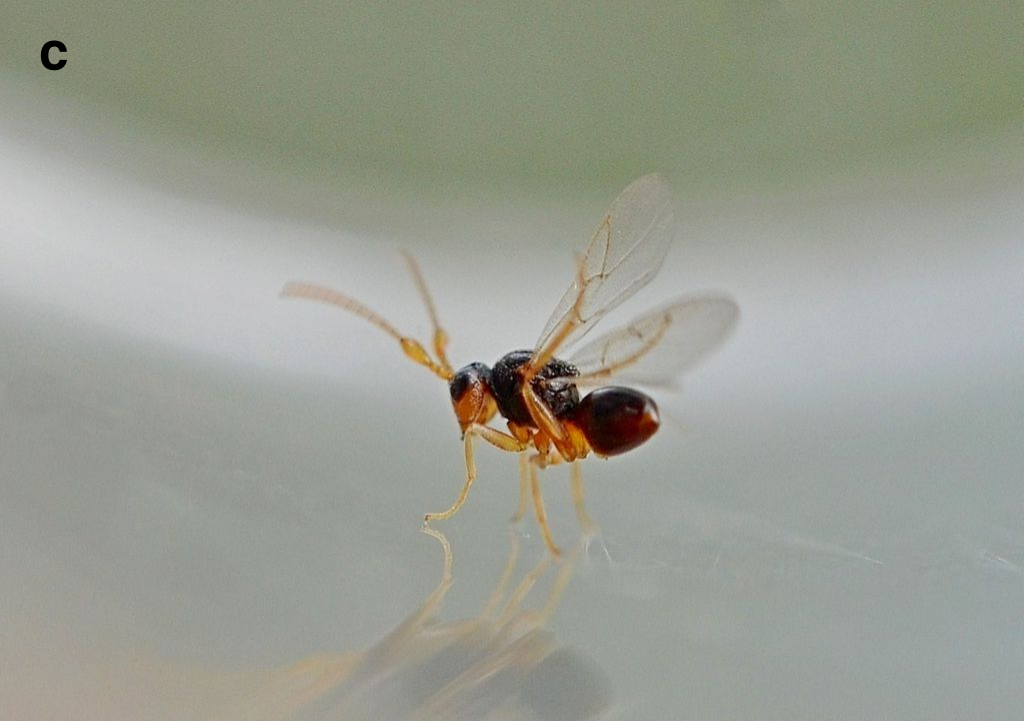
*Synergus thaumacerus* (Dalman) male (courtesy of E. Klimsa)

**Figure 2a.**
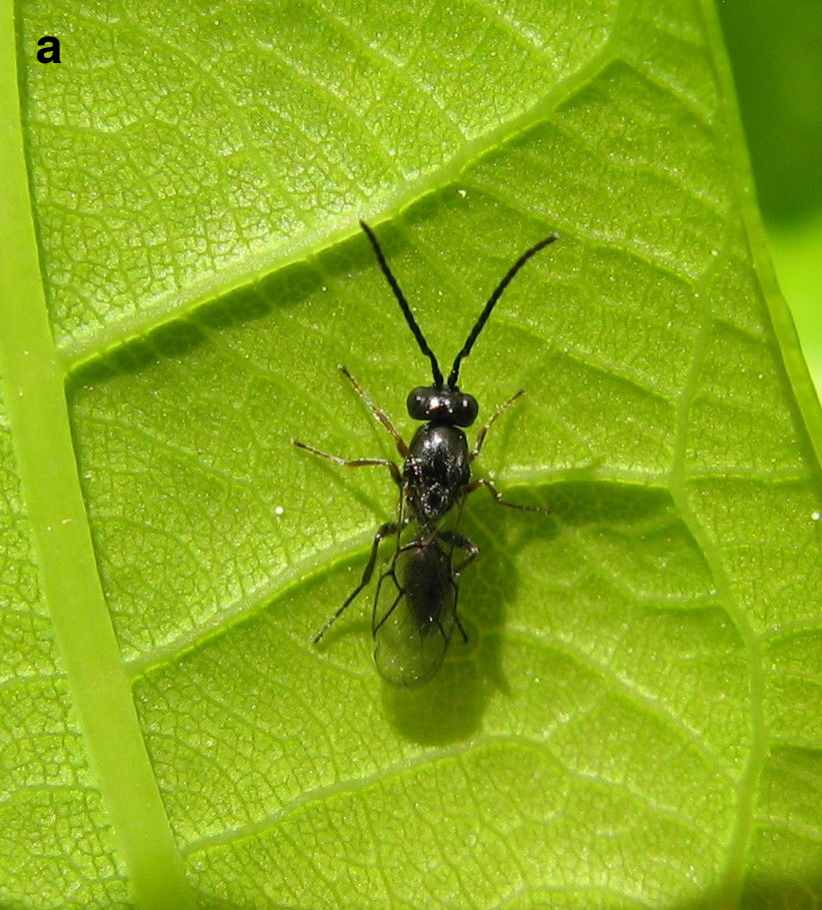
Male *Synergus* sp. (J. Bowdrey).

**Figure 2b.**
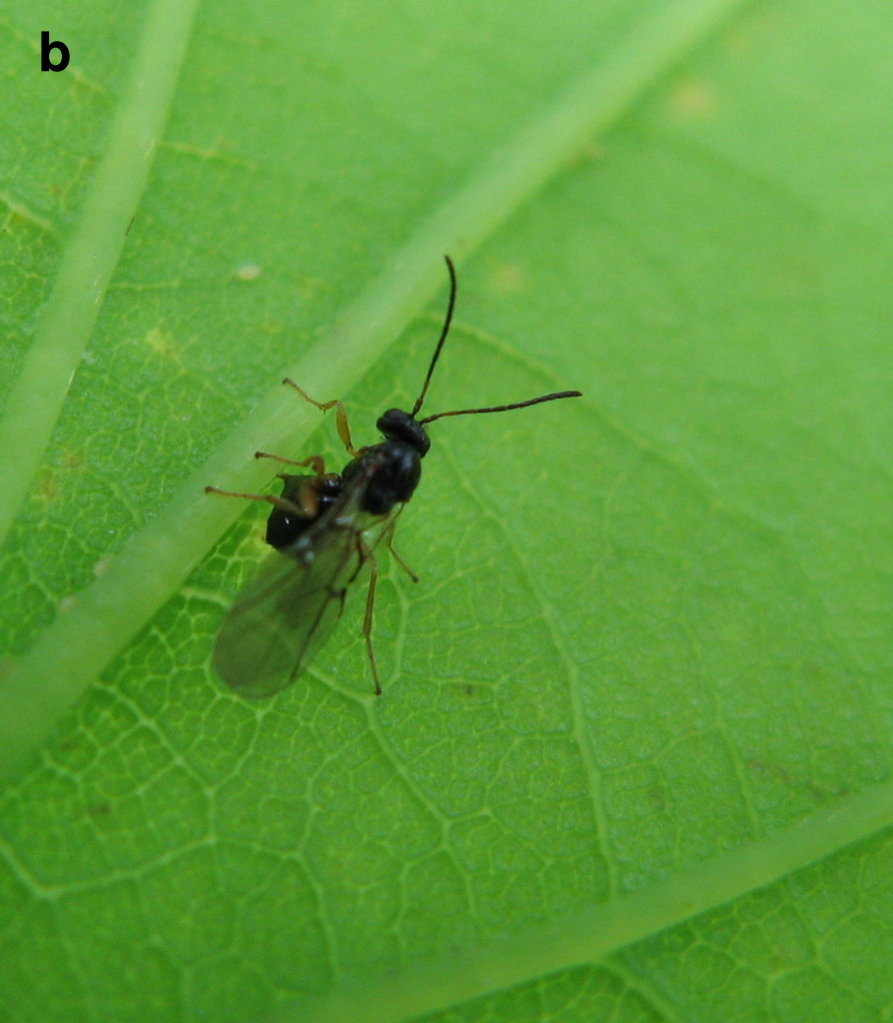
Ovipositing female *Neuroterus anthracinus* (Curtis) (J. Bowdrey).

**Figure 3a.**
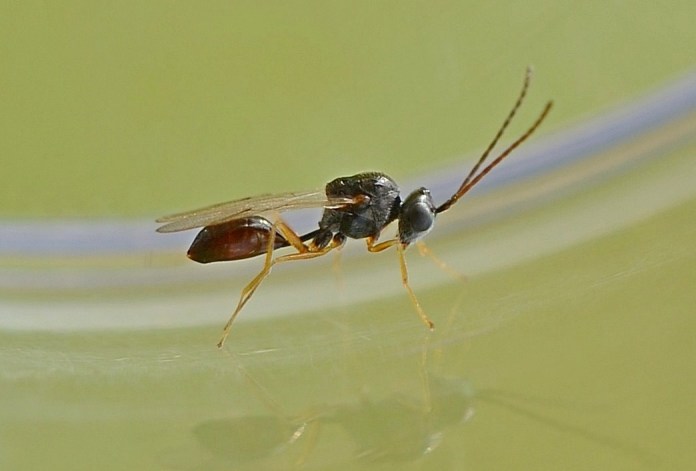
*Anacharis eucharoides* (Dalman) (Anacharitinae), female (courtesy of E. Klimsa)

**Figure 3b.**
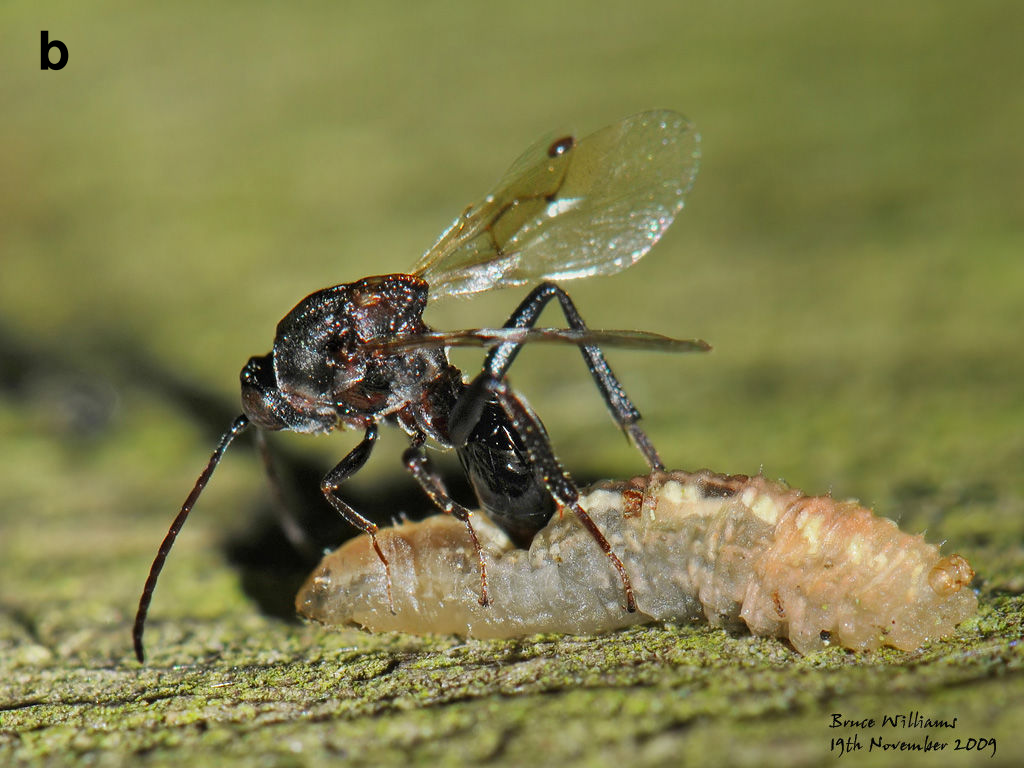
*Callaspidia defonscolombei* Dalman (Aspicerinae) female ovipositing in Syrphidae larva (courtest of B. Williams)

**Figure 3c.**
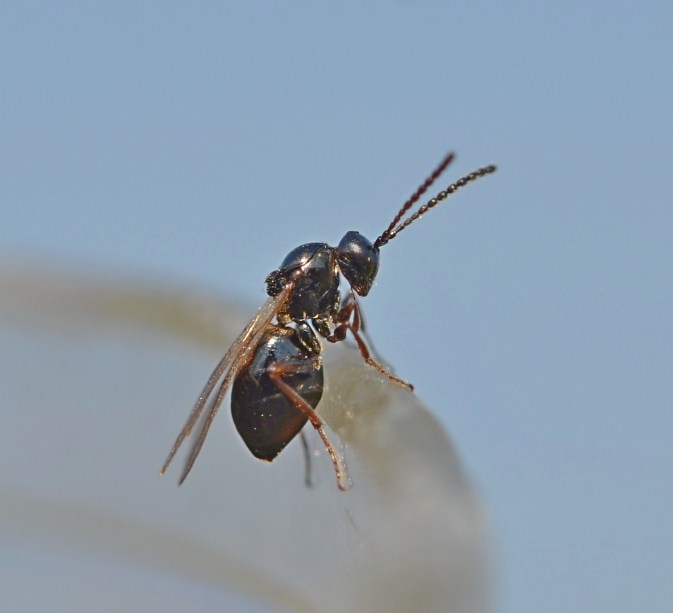
*Trybliographa* sp. (Eucoilinae), female (courtesy of E. Klimsa)

**Figure 3d.**
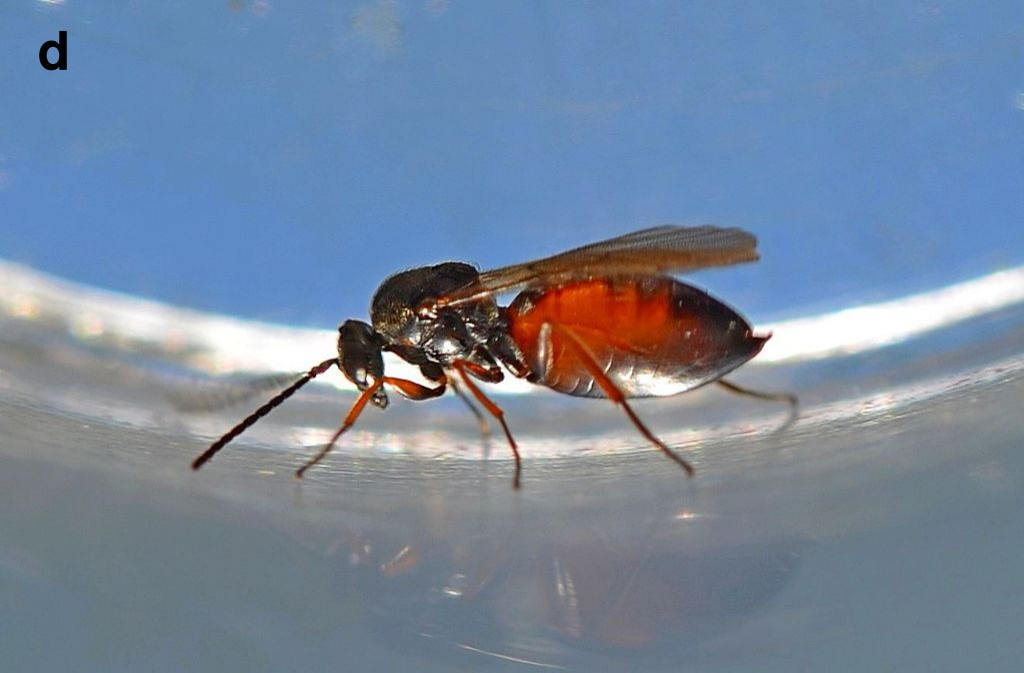
*Amphitectus areolatus* (Hartig) (Figitinae), female (courtesy of E. Klimsa)

**Table 1.** Numbers of conﬁrmed British and Irish Cynipoidea broken down by family and country, with numbers from the 1978 checklist (Quinlan 1978b) for comparison. Totals do not include uncertain identiﬁcations.

**family**	**total valid species 1978**	**total valid species 2017**	**England**	**Scotland**	**Wales**	**Ireland**	**Isle of Man**
** Cynipidae **	78 [inc. 1 introduced]	91 [inc. 2 introduced]	87	49	56	48	13
** Figitidae **	123	127	111	44	30	50	1
** Ibaliidae **	1	2	2	1	0	0	0
	**202**	**220**	**200**	**94**	**86**	**98**	**14**
