# Supplementary material for: Checklist of British and Irish Hymenoptera - Cynipoidea
Source: Biodivers Data J. 2017 Mar 9;(5):e8049. doi: 10.3897/BDJ.5.e8049 (PMC5345061; doi:10.3897/BDJ.5.e8049)
Supplement: Supplementary material 1 — Checklist of the British and Irish Cynipoidea [file bdj-05-e8049-s001.docx]

Superfamily Cynipoidea

The family and subfamily level classification follow Ronquist (1999). Authorship is as follows:

Cynipidae – J.P. Bowdrey & B.M. Spooner

Figitidae – M. Forshage, G.R. Broad & F. Van Veen

Ibaliidae – G.R. Broad

Synonymy for Cynipidae is mainly restricted to the better known names and all those that have appeared in the British literature. For additional synonymy see Melika (2006). It should be born in mind that future molecular studies may change our understanding of some species concepts and their alternating generations. Distribution data for Cynpidae are mainly derived by JPB from published sources, but thanks are due to the following for supplying additional data: Janet Boyd, Records Data Manager, British Plant Gall Society; Adrian Fowles, Countryside Council for Wales; Kate Hawkins, Manx Natural Heritage; David Notton, Natural History Museum, London; Mark Pavett, National Museum of Wales; (all pers. comm.).

Family Cynipidae Latreille, 1802^[[1]](#footnote-1)^

Tribe AULACIDEINI Nieves-Aldrey, 1994^[[2]](#footnote-2)^

AULACIDEA Ashmead, 1897

PSEUDAULAX Ashmead, 1903

follioti Barbotin, 1972 E added by Bowdrey (1994).

hieracii (Linnaeus, 1758, *Cynips*) E S W

*hieracii* (Bouché, 1834, Cynips)

sabaudi Hartig, 1840, Aylax

graminis Cameron, 1875, Aulax

*artemisiae* (Thomson, 1877, *Aulax*)

*crassinervis* (Thomson, 1877, *Aulax*)

*foveigera* (Thomson, 1877, *Aulax*)

nibletti Quinlan & Askew, 1969 S

pilosellae (Kieffer, 1901, Aulax) E

subterminalis Niblett, 1946 E

tragopogonis (Thomson, 1877, Aulax) E

pigeoti (Kieffer, 1898, Aulax)^[[3]](#footnote-3)^

species of Aulacidea excluded from the British and Irish list

[andrei (Kieffer, 1900, Aulax)^[[4]](#footnote-4)^]

ISOCOLUS Förster, 1869

EUBOTHRUS Förster, 1869

fitchi (Kieffer, 1898, Aulax)

jaceae (Schenck, 1863, Aulax) E S

affinis (Schenck, 1863, Aylax)

scabiosae (Giraud, 1859, Diastrophus) E

*areolatus* (Giraud, 1859, *Diastrophus*)

centaureae (Thomson, 1877, Aulax)

rogenhoferi Wachtl, 1880^[[5]](#footnote-5)^

LIPOSTHENES Förster, 1869

LIPOSTHENUS misspelling

glechomae (Linnaeus, 1758, Cynips) E S W M

latreillei (Kieffer, 1898, Aulax)

Tribe Aylacini Ashmead, 1903

AYLAX Hartig, 1840

AULAX Hartig, 1843

minor Hartig, 1840 E I

papaveris (Perris, 1840, Diplolepis) E S W

rhoeadis (Bouché, 1834, Cynips)

Tribe CEROPTRESINI Nieves-Aldrey, Nylander & Ronquist, 2015

CEROPTRES Hartig, 1840

cerri Mayr, 1873 E added by Jennings (2016)

*cerriphilus* Giraud in Houard, 1911

*vitripennis* Giraud in Houard, 1911

clavicornis Hartig, 1840 E S W I

*socialis* Hartig, 1840

arator Hartig, 1841

*melanonerus* Hartig, 1841

Tribe Cynipini Latreille, 1802^[[6]](#footnote-6)^

ANDRICUS Hartig, 1840

APHILOTHRIX Förster, 1869

LIODORA Förster, 1869

MANDERSTJERNIA Radoszkowski, 1866

TRICHOTERAS Ashmead, 1897

PARANDRICUS Kieffer, 1906

ADLERIA Rohwer & Fagan, 1917

EUSCHMITZIA Dettmer, 1925

ONCASPIS Dettmer, 1925

DROS Kinsey,1937

DRUON Kinsey, 1937

FERON Kinsey, 1937

CONOBIOS Kinsey, 1938

amenti Giraud, 1859 -s- E S^[[7]](#footnote-7)^

*callidoma* (Giraud, 1859, *Cynips*) (non Hartig, 1841) -a-

*giraudianu*s Dalla Torre and Kieffer, 1910 -a-

aries (Giraud, 1859, Cynips) -a- E W added by Leach (1999)^[[8]](#footnote-8)^

callidoma (Hartig, 1841, Cynips) -a- E S W I

cirratus Adler, 1881 -s-

giraudi Wachtl, 1882 **-**a**-**

corruptrix (Schlechtendal, 1870, Cynips)^[[9]](#footnote-9)^ -a- E S I

*ambigua* (Trotter, 1899 *Cynips*) **-**a**-**

*corruptrix* f. *elianae* Melika, Csóka & Pujade-Villar, 2000 nomen nudum **-**s-

**?***cryptobius* Wachtl, 1880 **-s-** E added by Bowdrey (2015)^[[10]](#footnote-10)^

curvator Hartig, 1840 -s- E S W I M

*axilaris* Hartig, 1840 -s**-**

collaris (Hartig, 1840, Cynips) -a**-**

*roeselii* (Dahlbom, 1842, *Cynips*) (nomen nudum) **-**s**-**

*dimidiatu*s (Schenck, 1863, *Spathegaster*) **-**s**-**

*fasciatus* Schenck, 1863 –a-

*perfoliatus* Schenck, 1863 -s-

*sulcata* (Förster, 1869, *Liodora*) **-**s**-**

*fasciata* (Schlechtendal, 1870, *Cynips*) **-**a**-**

*tegmentorum* (Schlechtendal, 1870, *Cynips*) **-**a**-**

foecundatrix (Hartig, 1840, Cynips) -a- E S W I M

fecundator misspelling

quercusgemmae (Linnaeus, 1758, Cynips)

gemmarum Lacaze-Duthiers, 1853 **-**a**-**

gemmae (Schenck, 1863, Cynips) -a*-*

pilosus Adler, 1881 -s-

gemmeus (Giraud, 1859, Cynips) -a- E added by Bowdrey (2009)^[[11]](#footnote-11)^

kirschbergi (Wachtl, 1876, Aphilothrix) -a-

*gemmae* Della Torre & Kieffer, 1910 **-**a**-**

**?**gemmicola Kieffer, 1901 E^[[12]](#footnote-12)^

glandulae (Hartig, 1840, Cynips) -a- E S I

xanthopsis Schlechtendal, 1884 -s-

grossulariae Giraud, 1859^[[13]](#footnote-13)^ -s- E added by Walker (2001b)

mayri (Wachtl, 1879, Cynips) -a-

panteli Kieffer, 1896 -a-

*gemellus* Belizin & Maisuradze, 1961 **-**s**-**

inflator Hartig, 1840 -s- E S W I .

globuli (Hartig, 1841, Cynips) -a-

*inflatioides* Belizin and Maisuradze, 1962 -?-

# kollari (Hartig, 1843, Cynips) -a- E S W I M^[[14]](#footnote-14)^

lignicola auctt. misident.

*quercusgemmae* Christ, 1791 preocc. **-**a**-**

circulans Mayr, 1870 -s-

legitimus Wiebes-Rijks, 1980 -a- E

lignicolus (Hartig, 1840, Cynips) -a- E S W I M

var*.* vanheurni van Leeuwen & Dekhuizen-Maasland, 1958 -s-

lucidus (Hartig, 1843, Cynips) -a- E^[[15]](#footnote-15)^

aestivalis Giraud, 1859 -s-

*erinaceus* (Kieffer, 1900, *Adleria*) -a-

malpighii (Adler, 1881, Aphilothrix)^[[16]](#footnote-16)^ -a- E S W I

nudus Adler, 1881 -s-

paradoxus (Radoszkowski, 1866, Manderstjernia)^[[17]](#footnote-17)^ -a- E S W I

albipunctata (Kaltenbach, 1867, *Cynips*) -a-

majalis (Giraud, 1868, Cynips) preocc. -a-

albopunctata (Schlechtendal, 1870, Cynips) -a-

lambertoni Kieffer, 1897 -a-

albopunctatus f. barbotini Folliot, 1964 -s-

quadrilineatus Hartig, 1840 -a- E S W I

ambiguus Schenck , 1863 **-**a**-**

glabriusculus Schenck, 1863 –a-

pedunculi Schenck, 1863 -a-

verrucosus Schenck, 1863 **-**a**-**

marginalis (Schlechtendal, 1870, Andricus)^[[18]](#footnote-18)^ -a-

*4-lineata* (Thomson, 1877, *Cynips*) **-**a-

kiefferi Pigeot, 1900 -s-

*quadrilineatus* f. *kiefferi* Folliot, 1964 **-**s-

quercuscalicis (Burgsdorff, 1783, Cynips) **-a-** E S W I

cerri (Beyerinck, 1896, Andricus) -s-

beyerincki Trotter, 1899 -s-

quercuscorticis (Linnaeus, 1761, Cynips) -a- E S W I M

corticis (Hartig, 1840, *Cynips*) unjustified emendation -a-

brevicornis (Hartig, 1841, Cynips) -s-

gemmatus Adler, 1881 -s-

krajnovici Tavares, 1901 -a-

quercusradicis (Fabricius, 1798, Cynips) -a- E S W I

noduli Hartig, 1840 -s-

trilineatus Hartig, 1840 -s-

parasiticus (Hartig, 1841, Neuroterus) -s-

*radicis* Hartig, 1841 -a-

*rugiscuta* Thomson, 1877 **-**s**-**

quercusramuli (Linnaeus, 1761, Cynips) -s- E S W I

autumnalis (Hartig, 1840, Cynips) -a-

amentorum (Hartig, 1843, Teras) **-**s**-**

*ramuli* Schenck, 1863 unjustified emendation -s**-**

rhyzomae (Hartig, 1843, Cynips) -a- E W^[[19]](#footnote-19)^

*ionescui* Kierych, 1965 -a-

seminationis (Giraud, 1859, Cynips)^[[20]](#footnote-20)^ -a- E S W

inflorescentiae (Schlechtendal, 1870, Cynips) **-**?**-**

sieboldi (Hartig, 1843, Cynips) -a- E W

*corticalis* (Hartig, 1840, *Cynips*) -a-

*quercuscorticis* (Bechstein & Scharfenberg, 1805 *Cynips*) preocc. **-**a**-**

*occidentalis* Folliot, 1964 **-**s**-**

sieboldi f. poissoni Folliot, 1964 -s-^[[21]](#footnote-21)^

singularis Mayr, 1870 **-s-** E added by Jennings (2014)

*singulus* Mayr, 1870 -s-

solitarius (Boyer de Fonscolombe, 1832, Diplolepis) -a- E S W I

ferruginea (Hartig, 1840, Cynips) **-**a**-**

occultus Tschek, 1871 -s-

gallaepyriformis (Olivier, 1791, Diplolepis) -a**-**

*filigranata* (Dettmer, 1925, *Oncaspis*) -s**-**

*villarrubiae* Tavares, 1930 -a-

***?testaceipes*** Hartig, 1840 **-s-** E?^[[22]](#footnote-22)^ Melika (2006)

var. *nodifex* Kieffer, 1900

Species of Andricus excluded from the British and Irish list

[clementinus (Giraud, 1859, Cynips)^[[23]](#footnote-23)^ -a-]

[gallaetinctoriae (Olivier, 1791, Diplolepis)^[[24]](#footnote-24)^ -a-]

*tinctoria* (Hartig 1843, *Cynips*) -a-

[gallaeurniformis (Boyer de Fonscolombe, 1832, Diplolepis)^[[25]](#footnote-25)^ -a-

sufflator Mayr, 1882 -s-]

[quercustozae (Bosc, 1792, Cynips)^[[26]](#footnote-26)^ -a-]

[trotteri Kieffer, 1898^[[27]](#footnote-27)^ -a-]

APHELONYX Mayr, 1881

cerricola (Giraud, 1859, Cynips) -a- E added by Crawley (1997)

BIORHIZA Westwood, 1840

APOPHYLLUS Hartig, 1840

TERAS Hartig, 1840 preocc.

PHILONIPS Walsh, 1864

HETEROBIUS Guérin-Méneville, 1865

DRYOTERAS Förster, 1869

HARTIGIA Rondani, 1871 preocc.

pallida (Olivier, 1791, Diplolepis) -s- E S W I M

aptera (Bosc, 1791, Cynips) preocc. -a-

*gallaealveariformis* (D’Anthoine, 1794, *Diplolepis*) **-**s-

*gallaecerebriformis* (D’Anthoine, 1794, *Diplolepis*) **-**s**-**

quercusterminalis (Fabricius, 1798, Cynips) -s-

terminalis (Hartig, 1840, Teras) unjustified emendation -s-

*sieboldi* (Stadelman, 1892*, Andricus*) (non Hartig, 1843) **-**a-

CALLIRHYTIS Förster, 1869

EUSYMPHAGUS Dettmer, 1930

bella (Dettmer, 1930, Eusymphagus) -s- E W I

erythrocephala (Giraud, 1859, Andricus) -a- E W

?erythrosoma Dettmer, 1933 **-a-**

erythrostoma Dettmer, 1933 **-a-**

hartigi Förster, 1869^[[28]](#footnote-28)^ -s- Pujade-Villar *et al*. (2007)^[[29]](#footnote-29)^

species of Callirhytis excluded from the British and Irish list

[glandium (Giraud, 1859, Andricus)^[[30]](#footnote-30)^ -a-

Cynips Linnaeus, 1758

DIPLOLEPIS misapplied

DRYOPHANTA Förster, 1869

agama Hartig, 1840 -a- E S W I

f.*mailleti* Folliot, 1964 -s-

disticha Hartig, 1840 -a- E S W I

f. indistincta Niblett, 1948 -s-

divisa Hartig, 1840 -a- E S W I

verrucosus (Schlechtendal, 1870, Spathegaster) -s-

longiventris Hartig, 1840 -a- E S W I

similis (Adler, 1881, Spathegaster) -s-

f. substituta Kinsey, 1930 -s-

quercusfolii Linnaeus, 1758^[[31]](#footnote-31)^ -a- E S W I

*floriiquercus* Gleditsch, 1774 **-**a**-**

scutellaris (Olivier, 1791, Diplolepis) -a-

*gallaecerasiformis* D’Anthoine, 1794 **-**a**-**

*gallaeunedoniformis* (D’Anthoine, 1794, *Diplolepis*) **-**a**-**

flosculi (Giraud, 1868, Spathegaster) -s-

giraudi (Tschek, 1869, Spathegaster) -s**-**

taschenbergi (Schlechtendal, 1870, Spathegaster) -s-

Species of Cynips excluded from the British and Irish list

[quercus (Fourcroy, 1785, Diplolepis)^[[32]](#footnote-32)^ -a-

pubescentis (Mayr, 1881, Dryophanta) -s-]

DRYOCOSMUS Giraud, 1859

# kuriphilus Yasumatsu, 1951 E^[[33]](#footnote-33)^ added by Malumphy (2015); EPPO (2015)

Species of *Dryocosmus* excluded from the British and Irish list

[cerriphilus Giraud, 1859^[[34]](#footnote-34)^ -a- ]

NEUROTERUS Hartig, 1840

SPATHEGASTER Hartig, 1840

AMERISTUS Förster, 1869

DOLICHOSTROPHUS Ashmead, 1887

NEOSPATHEGASTER Kinsey, 1923

DIPLOBIUS Kinsey, 1923

NEONEUROTERUS Monzen, 1954

REPENTINIA Belizin & Maisuradze, 1961

albipes (Schenck, 1863, Spathegaster)^[[35]](#footnote-35)^ -s- E S W I M

laeviusculus Schenck, 1863 -a-

pezizaeformis Schlechtendal, 1870 **-**a**-**

codinae Tavares, 1928 -s-

anthracinus (Curtis, 1838, Cynips)^[[36]](#footnote-36)^ -a- E S W I

flavipes (Boyer de Fonscolombe, 1832, Diplolepis) nom. dub. -a-

ostrea (Hartig, 1840, Cynips) -a-

furunculus Beyerinck, 1882 -s-

numismalis (Geoffroy in Fourcroy 1785, Cynips) -a- E S W I

numismatis (Olivier, 1790, Cynips) **-**a**-**

defectus Hartig, 1840 -a-

reaumuri Hartig, 1840 -a-

quercustiarae (Curtis, 1843, Cynips)

nigricornis Schenck, 1863 -a-

vesicatrix (Schlechtendal, 1870, Cynips) -s-

*vesicator* Hieronymus, 1890 -s-

*brunneus* Dettmer 1925 **-**s**-**

politus Hartig, 1840 -s- E S I

petioliventris (Hartig, 1840, Spathegaster) -s-

*bipunctatus* Hartig, 1841 **-**s**-**

*nitens* Hartig, 1841 **-**a**-**

*rubeculus* Hartig, 1841 **-**a**-**

aprilinus (Giraud, 1859, Spathegaster) -s-

*burgundus* (Schlechtendal, 1870, *Andricus*) preocc. **-**a**-**

schlechtendali Mayr, 1870 -a-

quercusbaccarum (Linnaeus, 1758, Cynips) -s- E S W I M

*quercuspedunculi* (Linnaeus, 1758, *Cynips*) **-**s**-**

*baccarumquercus* (Fourcroy, 1785, *Cynips*) **-**s**-**

*pedunculiquercus* (Fourcroy, 1785, *Cynips*) **-**s**-**

lenticularis (Olivier, 1791, Diplolepis) -a-

longipennis (Fabricius, 1793, Cynips) -a-

*gallaelenticulae* (D’Anthoine, 1794, *Diplolepis*) -a-

*gallaepisiformis* (D’Anthoine, 1794, *Diplolepis*) -s-

malpighii Hartig, 1840 -a-

interruptrix (Hartig, 1840, Cynips) -s-

interruptor (Hartig, 1841, Spathegaster) **-**s**-**

*baccarum* (Blanchard, 1849, *Cynips*) **-**s-

*pedunculi* (Duméril, 1860*, Diplolepis*) **-**s**-**

*attenuatus* Schenck, 1863 **-**a-

*striatus* Schenck, 1863 **-**a**-**

*pseudodisticha* (Küstenmacher, 1894, *Dryophanta*) **-**s**-**

tricolor (Hartig, 1841, Spathegaster) -s- E S W I

fumipennis Hartig, 1841 -a-

varius (Schenck, 1863, Spathegaster) -a-

Species of Neuroterus excluded from the British and Irish list

[punctatus (Bignell, 1892, Spathegaster)^[[37]](#footnote-37)^]

PLAGIOTROCHUS Mayr, 1881

FIORIA Kieffer, 1903

FIORIELLA Kiefer, 1903

# australis (Mayr, 1882, Dryocosmus) -a- E W

added by Robbins (2007)^[[38]](#footnote-38)^

coriaceus (Mayr, 1882, Andricus) -a- W added by Robbins (2007)^[[39]](#footnote-39)^

*pseudococcus* (Kieffer, 1902, *Andricus*) **-**a**-**

quercusilicis (Fabricius, 1798, Cynips) -s- E^[[40]](#footnote-40)^

added by Hancy & Hancy (2004)

*cocciferae* (Lichtenstein, 1877, *Andricus*) -s-

*ilicis* (Lichtenstein, 1877, *Andricus*) -s-

*emeryi* Mayr, 1882 -s-

fusifex Mayr 1882 -s-

PSEUDONEUROTERUS Kinsey, 1923

saliens (Kollar, 1857, Cynips) -a- E added by Redfern (2006)

saltans (Giraud, 1859, Neuroterus) -a-

glandiformis (Giraud, 1859, Spathegaster) -s-

TRIGONASPIS Hartig, 1840

XANTHOTERAS Ashmead, 1897

BELIZINELLA Kovatev, 1965

USSURASPIS Kovalev, 1965

megaptera (Panzer, 1801, Cynips) -s- E S W I

crustalis Hartig, 1840 -s-

renum (Hartig, 1840, Cynips) -a-

Species of Trigonaspis excluded from the British and Irish list

[synaspis (Hartig, 1841, Apophyllus)^[[41]](#footnote-41)^ -a-

megapteropsis Wriese, 1900 -s-]

Tribe diastrophini Nieves-Aldrey, Nylander & Ronquist, 2015

DIASTROPHUS Hartig, 1840

*GONASPIS* Ashmead, 1897

rubi (Bouché, 1834, Cynips) E W I

aphidivorus Cameron, 1889

hartigi (Marshall, 1867, Andricus)

species of Diastrophus excluded from the British and Irish list

[mayri Reinhard, 1876^[[42]](#footnote-42)^]

PERICLISTUS Förster, 1869

brandtii (Ratzeburg, 1831, Cynips) E S W

caninae (Hartig, 1840, *Aylax*) E S I

*germanus* (Schenck, 1863, *Aulax*)

*rosarum* Dettmer, 1925

**?**spinosissimae Dettmer, 1924^[[43]](#footnote-43)^ E S W I

XESTOPHANES Förster, 1869

brevitarsis (Thomson, 1877, Aulax) E S W I

tormentillae Schlechtendal, 1880

potentillae (Retzius, 1783, Cynips) E W I M

brevicornis (Curtis, 1838, Cynips) preocc.

splendens (Hartig, 1840, Aylax)

*laevigata* Schenck, 1863

abbreviatus (Thomson, 1877, Aulax)

*foveicollis* Thomson, 1877

Tribe PHANACIDINI Nieves-Aldrey, Nylander & Ronquist, 2015

PHANACIS Förster, 1860

GILLETTEA Ashmead, 1897

caulicola (Hedicke, 1939, *Aylax*) E W

centaureae (Förster, 1860, Cynips) E S

*punctipleuris* (Thomson, 1877, *Aulax*)

*karadagica* Diakontschuk, 1980

*parvulus* Diakontschuk, 1980

hypochoeridis (Kieffer, 1887, Aulax) E W I M

*seriol*ae Stefani, 1903

**?**taraxaci (Ashmead, 1897, Gillettea) E S^[[44]](#footnote-44)^

TIMASPIS Mayr, 1881^[[45]](#footnote-45)^

**?*l***ampsanae (Perris, 1873, Aulax) E^[[46]](#footnote-46)^

lusitanica Tavares, 1904 E added by Jennings (2005)

species of Timaspis excluded from the British and Irish list

[sonchi (De Stefani, 1900, Aulax)^[[47]](#footnote-47)^]

Tribe DIPLOLEPIDINI Latreille, 1802

Rhoditini Hartig, 1840

DIPLOLEPIS Geoffroy, 1762

RHODITES Hartig, 1840

HOLOLEXIS Förster, 1869

TRIBALIA Walsh, 1864

LYTORHODITES Kieffer, 1902

NIPPORHODITES Sakugami, 1949

eglanteriae (Hartig, 1840, Rhodites) E^[[48]](#footnote-48)^

*rufipes* (Förster, 1869, *Hololexis*)

mayri (Schlechtendal, 1877, Rhodites) E

*orthospinae* (Beijerinck, 1883, *Rhodites*)

nervosa (Curtis, 1838, Cynips) E W

centifoliae (Hartig, 1840, Rhodites) Pujade-Villar & Plantard (2002)

rosarum (Giraud, 1859, Rhodites)

*andrei* (Kieffer, 1904, *Rhodites*)

*kiefferi* (Loiselle, 1912, *Rhodites*)

dispar (Niblett, 1943, Rhodites)

rosae (Linnaeus, 1758, Cynips) E S W I M

bedeguaris Fourcroy, 1785

spinosissimae (Giraud, 1859, Rhodites) E S W I M

rosae-spinosissimae (Inchbald, 1861, Cynips)

Tribe SYNERGINI Ashmead, 1896^[[49]](#footnote-49)^

SAPHONECRUS Dalla Torre & Kieffer, 1910

connatus (Hartig, 1840, Synergus) E S I

erythroneurus (Hartig, 1840, Synergus)

SYNERGUS Hartig, 1840

SAPHOLYTUS Förster, 1869

apicalis Hartig, 1841 E S

clandestinus Eady, 1952 E W I

***consobrinus*** Giraud, 1911 E added by Jennings (2017)

crassicornis (Curtis, 1838, Cynips) E W I

evanescens Mayr, 1872

*fidelis* Tavares, 1920

*carinulatus* Dettmer, 1924

facialis Hartig, 1840^[[50]](#footnote-50)^ E S W I

*gallaepomiformis* misident.

incrassatus Hartig, 1840 E S W I

*bipunctatus* Hartig, 1841

*crassicornis* Hartig, 1843 preocc.

pallicornis Hartig, 1841 E W I

*pallidicornis* Dalla Torre, 1893

pallidipennis Mayr, 1872 E W

pallipes Hartig, 1840 E W I

flavicornis Hartig, 1840

nervosus Hartig, 1840

nigripes Hartig, 1840

albipes Hartig, 1841

erythrocerus Hartig, 1841

*variolosus* Hartig, 1841

*varius* Hartig, 1841

*xanthocerus* Hartig, 1841

tscheki Mayr, 1872

tristis Mayr, 1873

*hartigi* Giraud, 1911

*fulvipes*, Dettmer,1924

*mutabilis*, Dettmer, 1924

radiatus Mayr, 1872 E

reinhardi Mayr, 1872 E S W I

ruficornis Hartig, 1840 E I

thaumacerus (Dalman, 1823, Cynips) E

klugii Hartig,1840

carinatus Hartig, 1841

*testaceus* (Hartig, 1841, *Xystus*)

*thaumatocerus* Dalla Torre, 1893 (unjustified emendation)

*inflatus* Giraud, 1911

*vesiculosus* Giraud, 1911

*inflatus* Dettmer, 1924 preocc.

tibialis Hartig, 1840

erythrostomus Hartig, 1841

immarginatus Hartig, 1841

rotundiventris Mayr, 1872

umbraculus (Olivier, 1791, Diplolepis) E S W I

*gallaeumbraculatae* (D’Anthoine, 1794, *Diplolepis*)

rufipes (Boyer de Fonscolombe, 1832, Diplolepis)

orientalis Hartig, 1841

melanopus Hartig, 1843

socialis Hartig, 1843

*punctatus* Dettmer, 1924 preocc.

***variabilis*** Mayr, 1872 E added by Chinery & Williams in Melika (2006)

*cerridis* Giraud, 1911

*conformis* Giraud, 1911

*cerricolus* Vassileva-Samnalieva, 1986

species of Synergus excluded from the British and Irish list

[hayneanus (Ratzeberg, 1833, Cynips)^[[51]](#footnote-51)^]

Family Figitidae Hartig, 1840^[[52]](#footnote-52)^

Subfamily Anacharitinae, Thomson, 1862

MEGAPELMINAE Förster, 1869

ACANTHAEGILIPINAE Kovalev, 1979 Ronquist (1999)

PROANACHARITINAE Kovalev, 1979 Ronquist (1999)

Aegilips Haliday in Walker, 1835

atricornis Fergusson, 1985 E I added by Fergusson (1985)

nitidula (Dalman, 1823, Cynips) E I M

fumipennis (Westwood, 1833, Anacharis)

rufipes (Westwood, 1833, Anacharis)

dalmani Reinhard, 1860

rugicollis Reinhard, 1860

ruficornis Cameron, 1883

striolata Cameron, 1883

bicolorata Cameron, 1887

romseyensis Fergusson, 1985 E added by Fergusson (1985)

vena Fergusson, 1985 E S added by Fergusson (1985)

Anacharis Dalman, 1823^[[53]](#footnote-53)^

MEGAPELMUS Hartig, 1840

SYNAPSIS Förster, 1869 preocc.

PROSYNAPSIS Dalle Torre & Kieffer, 1910

eucharoides (Dalman, 1818, Cynips) E I

tinctus Walker, 1835

typica Walker, 1835

petiolata (Zetterstedt, 1838, Cynips)

spheciformis (Hartig, 1840, Megapelmus)

eucharioides misspelling

immunis Walker, 1835 E I

ensifer Walker, 1835

rufiventris (Hartig, 1841, Megapelmus)

staegeri Dahlbom, 1842

aquisgranensis (Förster, 1869, Synapsis)

Xyalaspis Hartig, 1843^[[54]](#footnote-54)^

CONASPICERA Hedicke, 1914

armata (Giraud, 1860, Aegilips) E S I

abietina (Giraud, 1860, Aegilips)

scotica (Cameron, 1883, Aegilips)

petiolata Kieffer, 1901 E S I

subulifera misident.

Subfamily Aspicerinae Dalla Torre & Kieffer, 1910

Onychiinae Thomson, 1862

Aspicera Dahlbom, 1842

Onychia Haliday in Curtis, 1829 preocc.

Bellona Giraud, 1860

scutellata (Villers, 1789, Tenthredo) I^[[55]](#footnote-55)^

ediogaster (Rossi, 1790, Evania)

bicolor (Boyer de Fonscolombe, 1832, Figites)

aculeator (Boyer de Fonscolombe, 1832, Figites)

ruficollis Kieffer, 1907 (Aspicera scutellata var.)

Callaspidia Dahlbom, 1842^[[56]](#footnote-56)^

defonscolombei Dahlbom, 1842 E I

westwoodi Dahlbom, 1842

nigripes (Cameron, 1879, Onychia)

dufouri Giraud, 1860

fonscolombei Dahlbom, 1856 unjustified emendation

provancheri Ashmead, 1887

striolata (Cameron, 1888, Onychia)

areolata (Kieffer, 1901, Onychia)

rubricus Dettmer, 1924

vitripennis (Kieffer, 1901, Onychia dufouri var.)

minima (Kieffer, 1901, Onychia fonscolombei var.)

Melanips haliday in Walker, 1835^[[57]](#footnote-57)^

SCYTODES Hartig, 1840 preocc.

AMBLYNOTUS Hartig, 1843

Anolytus Förster, 1869

alienus Giraud, 1860 E I

opacus misident.

dalmanni (Dahlbom, 1842, Figites) nom. nud.

longitarsus (Reinhard, 1860, Amblynotus)

microcerus (Kieffer, 1903, Amblynotus) E I

opacus (Hartig, 1840, Scytodes) E I

femoralis Cameron, 1883

sylvanus Giraud, 1860 E S W I

rufipes Förster, 1869

biusta (Cameron, 1879, Omalaspis)

Omalaspis Giraud, 1860

TAVARESIA Kieffer, 1901

LAMBERTONIA Kieffer, 1901

carinata (Kieffer, 1901, Tavaresia) W

Subfamily Charipinae Dalla Torre & Kieffer, 1910^[[58]](#footnote-58)^

ALLOTRIINAE Thomson, 1862 unavailable

ALLOXYSTINAE Hellén, 1931

Dilytini kierych, 1979

LYTOXYSTINAE Kovalev, 1994 Ronquist (1999)

Alloxysta Förster, 1869^[[59]](#footnote-59)^

ALLOTRIA Westwood, 1833 preocc.

XYSTUS Hartig, 1840 preocc.

PEZOPHYCTA Förster, 1869

nephycta Förster, 1869

ADELIXYSTA Kierych, 1988

*CARVERCHARIPS* Kovalev, 1994

abdera Fergusson, 1986 E added by Fergusson (1986)

apteroidea Hellén, 1963 E det. Van Veen, added here^[[60]](#footnote-60)^

**arcuata** (Kieffer, 1902, Allotria)^[[61]](#footnote-61)^ S W

minuta misident.

*ligustri* Evenhuis, 1976

**basimacula** (Cameron, 1886, Allotria)^[[62]](#footnote-62)^ S

caledonica (Cameron, 1886, Allotria)

perplexa (Cameron, 1889, Allotria)

brachyptera (Hartig, 1840, Xystus) E S W I

brevis (Thomson, 1862, Allotria) E

minuta misident.

castanea (Hartig, 1841, Xystus)^[[63]](#footnote-63)^ E W

melanogaster misident.

maculicollis (Cameron, 1886, Allotria)

*ruficollis* (Cameron, 1883, Allotria)^[[64]](#footnote-64)^

ruficeps (Cameron, 1883, Allotria) preocc.

megaptera (Cameron, 1889, Allotria)

dubia Kieffer, 1902 (Alloxysta erythrothorax var.)

rubriceps (Kieffer, 1902, Allotria)

*semiclausa* Kieffer, 1904

pruni (Hedicke, 1928, Charips)

circumscripta (Hartig, 1841, Xystus)^[[65]](#footnote-65)^ E

citripes (Thomson, 1862, Allotria) E W

britannica Kieffer, 1902 (Alloxysta citripes var.)

albipes (Kieffer, 1904, Allotria)

*brevicella* Belizin, 1966

consobrina (Zetterstedt, 1838, *Cynips*)^[[66]](#footnote-66)^ E

*fuscicornis* (Hartig, 1841, Xystus) Ferrer-Suay *et al.* (2013*b*)

ancylocera (Cameron, 1886, Allotria)

brassicae (Ashmead, 1887, Allotria)

infuscata (Kieffer, 1902, Allotria)

*aphidae* (Froggatt, 1904, *Hypodiranchis*)

grioti (De Santis, 1937, Charips)

**crassa** (Cameron, 1889, Allotria)^[[67]](#footnote-67)^ S

cursor (Hartig, 1840, Xystus) E^[[68]](#footnote-68)^

castanea (Kieffer, 1904, Pezophycta) preocc.

**erythrothorax** (Hartig, 1840, Xystus)^[[69]](#footnote-69)^ E S

trapezoidea misident.

defecta (Hartig, 1841, Xystus)

nigriventris (Thomson, 1862, Allotria)

halterata (Thomson, 1862, Allotria)^[[70]](#footnote-70)^ E

leunisii (Hartig, 1841, Xystus) E W added by Van Veen et al. (2003)

macrophadnus (Hartig, 1841, Xystus) E S W

testacea misident.^[[71]](#footnote-71)^

aptera misident.

brachyptera misident.

fuscipes misident.

nigriventris misident.

macrophadna misspelling

filicornis (Cameron, 1889, Allotria)

scutellata Kieffer, 1902

rubromaculata Kieffer, 1902 (Alloxysta nigriventris var.)

marshalliana (Kieffer, 1900, Nephycta)^[[72]](#footnote-72)^ S

**mullensis** (Cameron, 1883, Allotria)^[[73]](#footnote-73)^ S

nigrita (Thomson, 1862, Allotria) W added by Baker (2013)

obscurata (Hartig, 1840, Xystus) E det. Van Veen, added here

homotoma Kieffer, 1904 (Alloxysta ullrichi var.)

pallidicornis (Curtis, 1838, Cynips)

minuta (Zetterstedt, 1838, Cynips)

forticornis (Giraud, 1860, Allotria)

basalis (Thomson, 1862, Allotria)

anthracina Andrews, 1978

pedestris (Curtis, 1838, Cynips) E W I

**piceomaculata** (Cameron, 1883, Allotria)^[[74]](#footnote-74)^ S W

pilipennis (Hartig, 1840, *Xystus*) E added here, det. J. Pujade-Villar (pers. comm.)

*flavicornis* (Hartig, 1840, *Xystus*) Ferrer-Suay *et al.* (2014*a*)

pleuralis (Cameron, 1879, Allotria) E S I

unicolor (Kieffer, 1902, Alloxysta pusilla var.)

gautieri Kieffer, 1922

***pusilla*** (Kieffer, 1902, *Allotria*) E added by Sanders & Van Veen (2010)

ramulifera (Thomson, 1862, Allotria) E added by Müller et al. (1999)

minuta (Hartig, 1840, Xystus)

discreta (Förster, 1869, Nephycta)

parvicellula (Kieffer, 1904, Allotria)

semiaperta Fergusson, 1986 E I added by Fergusson (1986)

tscheki (Giraud, 1860, Allotria) E^[[75]](#footnote-75)^

victrix (Westwood, 1833, Allotria) E S W

*fulviceps* (Curtis, 1838, Cynips) Pujade-Villar *et al.* (2011)

ruficeps (Zetterstedt, 1838, Cynips)

erythrocephalus (Hartig, 1840, Xystus)

tritici (Fitch, 1861, Allotria)

macrocera (Thomson, 1877, Allotria)

amygdali (Buckton, 1879, Cynips) nom. nud.

atriceps (Buckton, 1879, Cynips)

curvicornis (Cameron, 1883, Allotria)

lateralis (Kieffer, 1902, Allotria luteicornis var.)

luteiceps (Kieffer, 1902, Allotria victrix var.)

luteicornis (Kieffer, 1902, Allotria)

areolata (Kieffer, 1909, Charips)

grandicornis (Kieffer, 1904, Allotria)

*io* (Girault, 1932 *Sarorthrus*)

species of *Alloxysta* excluded from the British and Irish list^[[76]](#footnote-76)^

[flavicornis (Hartig, 1841, Xystus)]

[ullrichi (Giraud, 1860, Allotria)]

ullerichi misspelling

species of uncertain status

[***ignorata*** (Kieffer, 1900, *Dilyta*) nom. dub.^[[77]](#footnote-77)^]

Apocharips Fergusson, 1986

trapezoidea (Hartig, 1841, Xystus) E W

xanthocephala (Thomson, 1862, Allotria)

Dilyta Förster, 1869

CHARIPS Haliday in Marshall, 1870

GLYPTOXYSTA Thomson, 1877

DYLITA misspelling

subclavata Förster, 1869 E S W

microcera (Haliday, 1870, Charips)

heterocera (Thomson, 1877, Glyptoxysta)

*talitzkii* (Belizin, 1966, *Glyptoxysta*) Paretas-Martínez *et al.* (2011)

Phaenoglyphis Förster, 1869

Hemicrisis Förster, 1869^[[78]](#footnote-78)^

AULOXYSTA Thomson, 1877

BOTHRIOXYSTA Kieffer, 1902

CHARIPSELLA Bréthes, 1913

dolichocera (Cameron, 1889, Allotria) E I

***longicornis*** (Hartig, 1840, *Xystus*) W^[[79]](#footnote-79)^

ruficornis (Förster, 1869, Hemicrisis)^^[[80]](#footnote-80)^^ E W

salicis (Cameron, 1883, Allotria) E W

forticornis Cameron, 1888

villosa (Hartig, 1841, Xystus) E W

piciceps (Thomson, 1862, Allotria)

collina (Cameron, 1889, Allotria)

ambrosiae (Ashmead, 1898, Allotria)

carpentieri (Kieffer, 1902, Allotria)

foveigera (Kieffer, 1902, Allotria)

curvata (Kieffer, 1902, Allotria)

recticornis (Kieffer, 1902, Allotria)

subaptera (Kieffer, 1904, Alloxysta)

campyla (Kieffer, 1904, Alloxysta)

necans (Kieffer, 1909, Glyptoxysta)

numidica (Kieffer, 1909, Bothrioxysta)

*bifoveata* (Girault, 1931, *Glyptoxysta*)

*islandica* (Hellén, 1931, *Alloxysta*)

flavipes (Ionescu, 1963 Charips)

xanthocroa Förster, 1869 E

rufa (Thomson, 1877, *Allotria*)

obfuscata Kieffer, 1901

Subfamily EUCOILINAE Thomson, 1862^[[81]](#footnote-81)^

Tribe Diglyphosematini Belizin, 1961

Diglyphosema Förster, 1869

conjungens Kieffer, 1904 E W I

Disorygma Förster, 1869

ECTOLYTA Förster, 1869

ERISPHAGIA Förster, 1869

curtum (Giraud, 1860, Eucoila) E BMNH, det. Forshage, added here

depile (Giraud, 1860, Eucoila) E I

incrassata (Thomson, 1862, Cothonaspis)

divulgata Förster, 1869

Microstilba Förster, 1869

striolata Kieffer, 1901 S^[[82]](#footnote-82)^

heterogena (Giraud, 1860, Eucoila) auctt., sensu Cameron

Tribe EUCOILINI Thomson, 1862

Eucoila Westwood, 1833

eUCOELA Agassiz, 1846

LYTOSEMA Kieffer, 1901

PSILODORA Förster, 1869

crassinerva Westwood, 1833 E S I

boyenii (Hartig, 1840, Cothonaspis)

intermedia (Kieffer, 1901, Psilodora)

maculata (Hartig, 1840, Cothonaspis) E S

guerini Dahlbom, 1842

brevialata Belizin, 1973

leptopilina Förster, 1869^[[83]](#footnote-83)^

clavipes (Hartig, 1841, Cothonaspis) BMNH, det. Forshage, added here

fimbriata (Kieffer, 1901, Eucoela)^[[84]](#footnote-84)^ E

xanthoneura nec (Förster, 1869, Episoda) sensu Quinlan (1978*b*)

longipes (Cameron, 1883, Erisphagia) preocc.

xanthopum (Kieffer, 1904, Psilosema)

filicorne (Kieffer, 1904, Psilosema)

longicorne (Kieffer, 1907, Psilosema)

dolichocera (Hellén, 1960, Episoda)

heterotoma (Thomson, 1862, Eucoila)^[[85]](#footnote-85)^ E I

?musti (Rondani, 1875, Xystus)

monilicornis (Kieffer, 1904, Ganaspis)

subnuda (Kieffer, 1904, Ganaspis)

philippinensis (Kieffer, 1916, Erisphagia)

bochei (Weld, 1944, Pseudeucoila)

longipes (Hartig, 1841, Cothonaspis) E BMNH, det. Forshage, added here

Trybliographa Förster, 1869^[[86]](#footnote-86)^

Episoda Förster, 1869 Nordlander (1980)

IDIOMORPHA Förster, 1869

HYPOLETHRIA Förster, 1869

PSICHACRA Förster, 1869

ADIERIS Förster, 1869

PIEZOBRIA Förster, 1869

PILINOTHRIX Förster, 1869

ANECTOCLIS Förster, 1869

COTHONASPIS auctt. nec Hartig, 1840

DIMICROSTROPHIS Ashmead, 1886

DUSMETIOLA Tavares, 1924

EUCOILA auctt. nec Westwood, 1835

PSEUDEUCOILA Ashmead, 1903

agaricola (Thomson, 1862, Eucoila) E BMNH, det. Forshage, added here

albipennis (Thomson, 1861, Eucoila) E

spaniandra Kerrich & Quinlan, 1960

atra (Hartig, 1840, Cothonaspis) E S I

nigricornis Cameron, 1883

cubitalis (Hartig, 1841, Cothonaspis) E I

diaphana (Hartig, 1841, Cothonaspis) E S W I

erythrocera (Thomson, 1877, Eucoila)^[[87]](#footnote-87)^ E BMNH, det. Forshage, added here

fovealis (Thomson, 1862, Eucoila) E BMNH, det. Forshage, added here

glottiana (Cameron, 1883, Psichacra) E S

proxima (Cameron, 1889, Eucoila)

agaricorum (Kieffer, 1902, Eucoela)

gracilicornis (Cameron, 1888, Eucoila) E S W I

longicornis (Hartig, 1840, Cothonaspis) E I

gracilis (Dahlbom, 1846, Eucoila)

subspinosa (Kieffer, 1904, Cothonaspis)

mandibularis (Zetterstedt, 1838, Figites) E S

similis (Cameron, 1883, Psichacra)

testaceipes Cameron, 1883^[[88]](#footnote-88)^

nigripes (Giraud, 1860, Eucoila) E BMNH, det. Forshage, added here

rapae (Westwood, 1835, Eucoila) E S W I

coronata (Hartig, 1841, Cothonaspis)

insignis (Giraud, 1860, Eucoila)

octotoma (Thomson, 1862, Eucoila)

scutellaris Förster, 1869

scutellaris nec (Latreille, 1805, Figites) sensu Hartig, 1840

melanocera (Förster, 1869, Idiomorpha)

crassicornis Cameron, 1889

fortinervis (Cameron, 1889, Eucoila)

ventralis (Kieffer, 1901, Eucoela)

ruficornis (Kieffer, 1902, Eucoela)

erythrocera nec (Thomson, 1862, Eucoela) sensu Cameron, 1890

britannica (Kieffer, 1905, Eucoela)

rufula (Förster, 1855, Eucoila) E I

dalei (Cameron, 1879, Psichacra)

scotica (Cameron, 1889, Eucoila)^[[89]](#footnote-89)^ E S

strandi (Hedicke, 1914, Cothonaspis) E BMNH, det. Forshage, added here

subnebulosa (Giraud, 1860, Eucoila) E BMNH, det. Forshage, added here

trichopsila (Hartig, 1841, Cothonaspis) E

brachytricha (Kieffer, 1901, Eucoela)

claripennis (Thomson, 1862, Eucoila)

Tribe GANASPINI Belizin, 1961

Chrestosema Förster, 1869

RECENTIA Belizin, 1961

erythropum Förster, 1869 E BMNH, det. Forshage, added here

[antennale Kieffer, 1904^[[90]](#footnote-90)^ E I]

DIDYCTIUM Riley, 1879

HEPTAMEROCERA Ashmead, 1896

nigriclava (Kieffer, 1904, Cothanaspis) E W BMNH, det. Forshage, added here

GANASPIS Förster, 1869

mundata Förster, 1869 E BMNH, det. Forshage, added here

tenuicornis Kieffer, 1904

seticornis (Hellén, 1960, Episoda) E S BMNH, det. Forshage, added here

ciliaria (Belizin, 1968, Odonteucoila)

Glauraspidia Thomson, 1861

AGLAOTOMA Förster, 1869

APHISTOPHYZA Förster, 1869

DIRANCHIS Förster, 1869

CRYPTEUCOELA Kieffer, 1904

AGLAOTOMIDEA Rohwer & Fagan, 1917

microptera (Hartig, 1840, Cothonaspis) E W I

codrina (Hartig, 1841, Cothonaspis)

subtilis (Dahlbom, 1842, Eucoela)

carpentieri Kieffer, 1901

giraudi (Kieffer, 1902, Aglaotoma)

foersteri (Kieffer, 1904, Aglaotoma)

giraudi (Kieffer, 1904, Crypteucoela) preocc.

elegans Ionescu, 1963

Hexacola Förster, 1869

HEXAPLASTA Förster, 1869

hexatoma (Hartig, 1841, Cothonaspis) E

picicrus (Giraud, 1860, Eucoila)

fuscipes (Mayer, 1923, Cothonaspis)

Mirandicola Belizin, 1968

Pseudopsichacra Quinlan, 1975

sericea (Thomson, 1877, Glauraspidia) E I

bispinosa (Kieffer, 1901, Eucoela)

sauteri (Hedicke, 1913, Psichacra)

Tribe KLEIDOTOMINI Hellén, 1960

Cothonaspis Hartig, 1840

PSILOSEMA Kieffer, 1901

gracilis Hartig, 1841 E

giraudi (Dalla Torre & Kieffer, 1910, Erisphagia)

longula Nordlander, 1976 E

pentatoma Hartig, 1841 E S

Eutrias Förster, 1869

tritoma (Thomson, 1877, Eucoila) E

Kleidotoma Westwood, 1833^[[91]](#footnote-91)^

APHYOPTERA Förster, 1869

APHILOPTERA Förster, 1869

AGROSCOPA Förster, 1869

HEPTAMERIS Förster, 1869

NEDINOPTERA Förster, 1869

RHYNCHACIS Förster, 1869

PENTACRITA Förster, 1869

TETRAHOPTRA Förster, 1869

TETRATOMA Cameron, 1890

ARHOPTRA Kieffer, 1901

PENTARHOPTRA Kieffer, 1901

SCHIZOSEMA Kieffer, 1901

KLEIDOTOMIDEA Rohwer & Fagan, 1917

PENTAKLEIDOTA Weld, 1951

affinis Cameron, 1889 S

caledonica Cameron, 1888 E S I

ciliaris (Zetterstedt, 1838, Figites)^[[92]](#footnote-92)^ E S

melanopoda Cameron, 1888

dolichocera Thomson, 1877 E W I

carpentieri (Kieffer, 1904, Cleidotoma)

elegans Cameron, 1889 E S

filicornis Cameron, 1889 E

gracilicornis Cameron, 1889 E

?gryphus Thomson, 1861

halophila Thomson, 1861 E

hexatoma Thomson, 1862 E S

longicornis Cameron, 1889 E S

longipennis Cameron, 1889 E S I

marshalli Cameron, 1889 E I

antecella Belizin, 1964

nigra (Hartig, 1840, Cothonaspis) E S I

crassiclava Cameron, 1888

nigripes Cameron, 1888

brevicornis (Kieffer, 1904, Rhynchasis)

tetramora (Kieffer, 1904, Rhynchasis)

pentatoma Thomson, 1861 E S I

albipennis Cameron, 1886 nec Thomson, 1861

picipes Cameron, 1886 S I

psiloides Westwood, 1833 E W

pygmea (Dahlbom, 1842, Eucoila) E I

striata Cameron, 1886 E S

striaticollis Cameron, 1880 E S

subaptera (Walker, 1834, Figites) E W I

helgolandica (Förster, 1869, Agroscopa)

thomsoni Forshage, sp. nov., replacement name E S I

tetratoma Thomson, 1861 preocc.^[[93]](#footnote-93)^

tomentosa (Giraud, 1860, Eucoila) E

anisomera (Förster, 1869, Aphiloptera)

erythropa Thomson, 1877

truncata Cameron, 1889 E S

species of Kleidotoma excluded from the British and Irish list

[myrmecophila Kieffer, 1908^[[94]](#footnote-94)^]

Tribe TRICHOPLASTINI Kovalev, 1989

Rhoptromeris Förster, 1869^[[95]](#footnote-95)^

MIOMOERA Förster, 1869

HEXAMEROCERA Kieffer, 1901

STRIATELLIA Belizin, 1966

heptoma (Hartig, 1840, Cothonaspis) E S W I

biscapus (Hartig, 1840, Cothonaspis)

eucera (Hartig, 1841, Cothonaspis)

nodosa (Giraud, 1860, Eucoila)

parvula (Thomson, 1862, Eucoila)

aberrans (Förster, 1869, Miomoera)

aequalis (Kieffer, 1901, Eucoela)

widhalmi Kurdjumov, 1912

villosa (Hartig, 1840, Cothonaspis) E W I BMNH, det. Forshage, added here

Species of Rhoptromeris excluded from the British and Irish list

[nigriventris Nordlander, 1978^[[96]](#footnote-96)^]

trichoplasta Benoit, 1956

sp. indet. BMNH, det. Forshage, added here^[[97]](#footnote-97)^

Subfamily Figitinae Hartig, 1840^[[98]](#footnote-98)^

AmphitHectus Hartig, 1840^[[99]](#footnote-99)^

areolatus (Hartig, 1840, Sarothrus) E

dahlbomii Hartig, 1840

piceus (Dahlbom, 1842, Figites) nom. nud.

fumipennis (Giraud, 1860, Melanips)

Figites Latreille, 1802

PSILOGASTER Hartig, 1840

Pycnotrichia Förster, 1869

OMALOSPOIDES Hedicke, 1913

SEITNERIA Tavares, 1928

anthomyiarum Bouché, 1834 E I

consobrinus Giraud, 1860 E I

scutellaris misident.

dentiscuta Hellén, 1937 Fergusson (1986)

ictus Fergusson, 1986 E I added by Fergusson (1986)

scutellaris (Rossi, 1794, Cynips) E

ruficornis (Rossi, 1794, Cynips)

abbreviator (Herrich-Schäffer in Panzer, 1801, Ophion)

tibialis (Hartig, 1840, Psilogaster)

letzneri (Hedicke, 1913, Omalaspoides)

Species of Figites excluded from the British and Irish list^[[100]](#footnote-100)^

[laevigatus Dahlbom, 1842]

[reinhardi Kieffer, 1901]

[urticarum Dahlbom, 1842]

Lonchidia Thomson, 1862

clavicornis Thomson, 1862 E S I

maculipennis (Dahlbom, 1842, Figites) E I

Sarothrus Hartig, 1840

tibialis (Zetterstedt, 1838, Cynips) E W I

canaliculatus Hartig, 1840

silesiacus (Hedicke, 1913, Omalaspoides)

Xyalophora Kieffer, 1901

clavata (Giraud, 1860, Figites) W I

Zygosis Förster, 1869

DICERAEA Förster, 1869

THYREOCERA Ashmead, 1887

urticeti (Dahlbom, 1842, Figites) E I

heteropterus (Hartig, 1843, Psilogaster)

flavicornis Hellén, 1937 Fergusson (1986)

Family Ibaliidae Thomson, 1862

Ibalia Latreille, 1802

SAGARIS Panzer, 1806

leucospoides (Hockenwarth, 1785, Ichneumon) E

cultellator (Fabricius, 1793, Ichneumon)

ensiger Norton, 1862

suprunenkoi Jacobson, 1899

gigantea Yoshimoto, 1970

rufipes Cresson, 1879 E S

drewseni Borries, 1891

shirmeri Dalla Torre & Kieffer, 1910

**References**

Adler, H. and Stratton, C.R. 1894. *Alternating generations. A biological study of oak galls and gall flies.* Oxford: Clarendon Press.

Askew, R. R. 1959. A note on *Callirhytis glandium* (Giraud) (Hym., Cynipidae). *Entomologist’s Monthly Magazine* **95**: 79.

Bagnall, R. S. 1917. *Aylax taraxaci* (Ashm.) a cynipid (Hymenoptera) new to the British fauna and notes on other gall wasps. *Entomologist’s Monthly Magazine* **53**: 200-210.

Bagnall, R. S. 1931. *Aulacidea andrei* (Kieff.), a cynipid (Hymenoptera) new to the British fauna. *Entomologist’s Monthly Magazine* **67**: 243.

Bagnall, R. S. 1932. On the Scottish species of gall-wasps that affect the Compositae. *Scottish Naturalist* **193**: 21-23.

Bagnall, R. S. and Burkill, H. J. 1935. A cynipid *Timaspis sonchi* Stefani as British. *The Entomologist* **68**: 10.

Bagnall, R. S. and Harrison, J. W. H. 1918. On some Cynipid oak-galls new to the British Fauna. *Entomologist’s Monthly Magazine* **54**: 177-182.

Bagnall, R. S. and Harrison, J. W. H. 1919. Talks about plant galls VI. The wasp galls of the British oak. *Vasculum* **5**: 127-134.

Bagnall, R. S. and Harrison, J. W. H. 1930. Preliminary records of two new British gall-wasps (Cynipidae) affecting the common goat’s beard (*Tragopogon pratensis*). *Entomologist’s Monthly Magazine* **66**: 225-226.

Bagnall, R. S. and Harrison, J. W. H. 1934. *Timaspis lampsanae* Karsch, a genus and species of cynipid gall causer new to the British Isles. *Entomologist’s Monthly Magazine* **70**: 62.

Baker, E. A. 2013. Aphid parasitoids in Wales. *British Journal of Entomology and Natural History* **26**: 219-239.

Bellido, D., Melika, G. & Pujade-Villar, J. 2005. Taxonomic status of *Andricus corruptrix*, *A. amblycerus* and *A. ambiguus* (Hymenoptera, Cynipidae). *Zeitschr. Entomologie* 26(4): 29 – 44.

Biggs, D. T. 2011. Additional records of plant galls from the Isle of Wight – 2010. *Proceedings of the Isle Wight Natural History and Archaeological Society* **25**: 172-173.

Bignell, G. C. 1892. A new species of Cynipidae. *Entomologist’s Monthly Magazine* **3**: 176-177.

Bowdrey, J. P. 1994. A preliminary note on *Aulacidea follioti* Barbotin, 1972 (Hymenoptera, Cynipidae), a species new to Britain. *Cecidology* **9**: 54.

Bowdrey, J. P. 1999. The Essex species of gall-inducing Cynipids (Hymenoptera: Cynipidae: Aulacidini and Rhoditini) on host plants other than *Quercus*. *Essex Naturalist (N.S.)* **16**: 110-124.

Bowdrey, J. P. 2009. *Andricus gemmeus* (Giraud, 1859) a gall wasp (Hymenoptera: Cynipidae) new to the British Isles. *Cecidology* **24**: 34-38.

Bowdrey, J. P. 2015. A new British gall on *Quercus cerris*, possibly induced by *Andricus cryptobius* Wachtl, 1880 (Hymenoptera: Cynipidae). *Cecidology* **30**: 77-78.

Buffington, M. L., Nylander, J. A. A. and Heraty, J. M. 2007. The phylogeny and evolution of Figitidae (Hymenoptera: Cynipoidea). *Cladistics* **23**: 403-431.

Burkill, H. J. 1933. Plant gall records for 1932. *London Naturalist* **1932**: 117-119.

Cameron, P. 1893. *A monograph of the British phytophagous Hymenoptera.* . London: Ray Society.

Connold, E. T. 1909. *Plant galls of Great Britain*. London: Adlard.

Crawley, M. J. 1997. *Aphelonyx cerricola* Giraud (Hym., Cynipidae), an alien gall-former new to Britain. *Entomologist’s Monthly Magazine* **133**: 61.

Dalla Torre, C. G. de and Kieffer, J. J. 1910. *Cynipidae. Das Tierreich, 24*. Berlin: Friedlander & Sohn.

Eady, R. D. 1952. A revision of section 1 (Mayr, 1872) of the genus *Synergus* (Hym. Cynipidae) in Britain, with a species new to science. *Transactions of the Society for British Entomology* **11**: 141-152.

Eady, R. D. and Quinlan, J. 1963. Hymenoptera, Cynipoidea. *Handbooks for the Identification of British Insects* **VIII (1a)**: 1-81.

Eady, R. D. and Quinlan, J. 1967. On the occurrence of *Andricus quercustozae* Bosc in Britain (Hym., Cynipidae). *Entomologist's Gazette* **18**: 10-12.

Entwistle, P. F. and Hails, R. S. 1997. An undescribed form, *borealis*, of the agamic phase of the smooth spangle gall, *Neuroterus albipes* (Schenck) (Hymenoptera: Cynipidae), in the Highland Region of Scotland with an analysis of its distribution on oak leaves. *Cecidology* **12**: 44-61.

EPPO 2015. First report of *Dryocosmus kuriphilus* in the United Kingdom. *EPPO Reporting Service* **6**: 2015/2108.

Evenhuis, H. H. 1982. A study of Hartig's *Xystus* species with type designations and new synonyms (Hymenoptera: Cynipidae Alloxystinae and Charipinae). *Spixiana* **5**: 19-29.

Evenhuis, H. H. 1985. Studies on Alloxystidae (Hymenoptera, Cynipoidea) 8. *Cynips minuta* Zetterstedt and *Xystus minutus* Hartig. *Entomologische Berichten* **45**: 16-20.

Fergusson, N. D. M. 1985. British species of the parasitic cynipid-wasp genus *Aegilips* (Hymenoptera: Cynipoidea, Anacharitinae). *Journal of Natural History* **19**: 811-818.

Fergusson, N. D. M. 1986. Charipidae, Ibaliidae & Figitidae (Hymenoptera: Cynipoidea). *Handbooks for the Identification of British Insects* **8 Pt.1c**: 1-55.

Ferrer-Suay, M., Paretas-Martínez, J., Selfa, J. and Pujade-Villar, J. 2012a. Taxonomic and synonymic world catalogue of the Charipinae and notes about this subfamily (Hymenoptera: Cynipoidea: Figitidae). *Zootaxa* **3376**: 1-92.

Ferrer-Suay, M., Selfa, J. and Pujade-Villar, J. 2012b. Taxonomic revision of the *Alloxysta brevis* group (Hymenoptera, Cynipoidea, Figitidae, Charipinae). *Boletín de la Sociedad Entomológica Aragonesa* **51**: 237-249.

Ferrer-Suay, M., Selfa, J. and Pujade-Villar, J. 2012c. Revision of V.I. Belizin’s type material of Alloxysta (Hymenoptera: Figitidae: Charipinae) deposited in the Zoological Institute of the Russian Academy of Sciences. *Zoosystematica Rossica* **21**: 279‑290.

Ferrer-Suay, M., Selfa, J. and Pujade-Villar, J. 2012d. Revision of Ionescu type material related with *Alloxysta* genus (Hym., Figitidae: Charipinae) deposited in the Muzeul de Istoria Naturala "Grigore Antipa", Bucharest, Romania. *Travaux du Museum d'Histoire Naturelle "Grigore Antipa"* **55**: 277‑284.

Ferrer-Suay, M., Selfa, J., Notton, D. G. and Pujade-Villar, J. 2013a. Revision of the types of species of *Alloxysta* described by Cameron and Fergusson (Hymenoptera: Figitidae: Charipinae) and deposited in the Natural History Museum (London), including a key to the fauna of Great Britain. *European Journal of Taxonomy* **53**: 1-27.

Ferrer-Suay, M., Selfa, J. and Pujade-Villar, J. 2013b. The *Alloxysta*(Hymenoptera: Figitidae: Charipinae) type material in the United States National Museum of Natural History and the Canadian National Collection of Insects. *Canadian Entomologist* **145**: 603‑625.

Ferrer-Suay, M., Selfa, J. and Pujade-Villar, J. 2013c. Revision of the Thomson and Zetterstedt collections of the genus *Alloxysta* Förster (hymenoptera: figitidae) deposited in the Lund Museum of Zoology (Sweden). *Entomologisk Tidskrift* **134**: 77‑102.

Ferrer-Suay, M., Selfa, J. and Pujade-Villar, J. 2013d. Revision of *Alloxysta* from the Curtis collection (Hymenoptera: Figitidae: Charipinae) deposited in Museum Victoria (Australia). *Memoirs of Museum Victoria* **70**: 11‑16.

Ferrer-Suay, M., Selfa, J. and Pujade-Villar, J. 2014a. Review of the Hartig type collection of *Alloxysta* (Hymenoptera: Figitidae: Charipinae) and other *Alloxysta*material deposited in the Zoologische Staatssammlung Museum (Munich). *Fragmenta Faunistica* **57**: 75‑116.

Ferrer-Suay, M., Selfa, J., Seco, M.V. and Pujade-Villar, J. 2014b. Revision of Hellén types of *Alloxysta*Förster (Hymenoptera: Figitidae, Charipinae). *Entomologica Fennica* **25**: 86‑101.

Ferrer-Suay, M., Selfa, J. and Pujade-Villar, J. 2015. New contribution to the knowledge of the genus *Alloxysta* (Hymenoptera: Cynipoidea: Figitidae): revision of some type material. *Annalen des Naturhistorischen Museums in Wien*, Serie B **117**: 23‑36.

Fitch, E. A. 1874. British oak-galls. *Entomologist’s Monthly Magazine* **11**: 109-110.

Fitton, M. G., Graham, M. W. R. de V., Boucek, Z. R. J., Fergusson, N. D. M., Huddleston, T., Quinlan, J. and Richards, O. W. 1978. Kloet and Hincks. A check list of British insects. Part 4: Hymenoptera. *Handbooks for the Identification of British Insects* **11**: ix + 159 pp.

Folliot, R. 1964. Contribution a l’étude de la biologie de cynipides gallicoles (Hymenoptera, Cynipoidea). *Annales des Sciences Naturelles, Zoologie* **ser. 6, 12**: 407-564.

Folliot, R., Ros-Farré, P., Bellido, J. and Pujade-Villar, J. 2004. Alternation of generations in *Andricus corruptrix* (Schlechtendal): comments on and descriptions of a new sexual form (Hymenoptera: Cynipidae). *Contributions to Zoology* **73**: 1-10.

Fordham, W. J. 1917. *Aylax taraxaci* Ashm. in Derbyshire. *Entomologist’s Monthly Magazine* **53**: 237.

Forshage, M. and Nordlander, G. 2008. Identification key to European genera of Eucoilinae (Hymenoptera, Cynipoidea, Figitidae). *Insect Systematics & Evolution* **39**: 341-359.

Forshage, M., Nordlander, G. and Buffington, M.L. 2013. Eucoilinae of North America: A revised catalog of genera and described species. *Proceedings of the Entomological Society of Washington* **115**: 225‑255.

Hancy, R. and Hancy, B. 2004. First British record of the cynipid *Plagiotrochus quercusilicis*. *Cecidology* **19**: 98.

Harrison, J. W. H. 1930. New and rare British galls, with some remarks on other species. *Journal of Botany* **68**: 39-44.

Jennings, M. T. 2005. *Timaspis lusitanica* Tavares, 1904 (Hym., Cynipidae) and an associated parasitoid *Eurytoma punctatella* Zerova, 1978 (Hym., Eurytomidae), both new to Britain. *Entomologist's Monthly Magazine* **141**: 160.

Jennings, M. T. 2014. *Andricus singularis* Mayr, 1870 (Hymenoptera: Cynipidae) new to the British Isles. *Entomologist's Monthly Magazine* **150**: 102.

Jennings, M. T. 2016. *Ceroptres cerri* Mayr (Hymenoptera, Cynipidae) new to Britain, with Kent records of *Ceroptres clavivornis* Hartig. *British Journal of Entomology and Natural History* **29**: 155.

Jennings, M. T. 2017. Andricus grossulariae (Giraud) and Synergus consobrinus Giraud - reared specimens from Kent, England. *Mendeley Data* v1: http://dx.doi.org/10.17632/kc75k37fjn.1

Jonsell, M., Nordlander, G. and Jonsson, M. 1999. Colonization patterns of insects breeding in wood-decaying fungi. *Journal of Insect Conservation* **3**: 145-161.

Leach, C. K. 1999. The ‘ramshorn’ gall of *Andricus aries* on *Quercus robur* – a cynipid gall to be added to the British list? . *Cecidology* **14**: 18-21.

Leeuwen, W. M. and Dekhuijzen-Maasland, J. M. 1958. The bigamic generations of *Andricus corruptrix* Schlechtendal and *Andricus lignicolus* Hartig (Hymenoptera, Cynipidae) Part II. *Tijdschrift voor Entomologie* **101**: 101-111.

Liljeblad, J. and Ronquist, F. 1998. A phylogenetic analysis of higher-level gall wasp relationships (Hymenoptera: Cynipidae). *Systematic Entomology* **23**: 229-252.

Malumphy, C. 2015. First findings of Oriental Chestnut Gall Wasp *Dryocosmus kuriphilus* Yasumatsu (Hymenoptera: Cynipidae) in the United Kingdom. *Cecidology* **30**: 45-47.

Mata-Casanova, M., Selfa, J. and Pujade-Villar, J. 2015. Revision of the genus Xyalaspis Hartig, 1843 (Hymenoptera: Figitidae: Anacharitinae) in the Western Palaearctic. *Zoosystema* **37**: 31-43.

Melika, G. 2006. Gall wasps of the Ukraine Cynipidae. *Vestnik zoologii* **Supplement 21**. 2 volumes.

Melika, G. and Abrahamson, W. G. 2002. Review of the world genera of oak cynipid wasps (Hymenoptera: Cynipidae: Cynipini). In: Melika, G., Thuróczy, C., editors. *Parasitic wasps: Evolution, Systematics, Biodiversity and Biological Control*. Budapest: Agroinform. p. 150-190.

Melika, G., Csóka, G. and Pujade-Villar, J. 2000. Check-list of oak gall wasps of Hungary, with some taxonomic notes (Hymenoptera: Cynipidae, Cynipinae, Cynipini). *Annales Historico-Naturales Musei Nationalis Hungarici* **92**: 265-296.

Menke, A. S. and Evenhuis, H. H. 1991. North-American Charipidae - key to genera, nomenclature, species checklists, and a new species of *Dilyta* Förster. *Proceedings of the Entomological Society of Washington* **93**: 136-158.

Morley, C. 1931. A synopsis of the British Hymenopterous family Cynipidae. *Entomologist* **64**: 206-210.

Mosley, S. 1892. Additional Yorkshire galls. *Naturalist (London)* **17**: 337-339.

Müller, C. B., Adriaanse, I. C. T., Belshaw, R. and Godfray, H. C. J. 1999. The structure of an aphid-parasitoid community. *Journal of Animal Ecology* **68**: 346-370.

Niblett, M., Ross, J. and Burkill, H. J. 1932. Gall-causing Cynipidae in Britain. Part II. *Entomologist* **65**: 274-275.

Nieves-Aldrey, J.-L. 1994. Revision of west European genera of the tribe Aylacini Ashmead (Hymenoptera, Cynipidae. *Journal of Hymenoptera Research* **3**: 175-206.

Nieves-Aldrey, J.-L. 2001. *Hymenoptera Cynipidae*. Madrid: Museo Nacional de Ciencias Superior de Investigaciones Cientificas.

Nordlander, G. 1978. Revision of the genus *Rhoptromeris* Förster, 1869 with reference to north-western European species. Studies on Eucoilidae (Hym.: Cynipoidea) II. *Entomologica Scandinavica* **9**: 47-62.

Nordlander, G. 1980. Revision of the genus *Leptopilina* Foerster, 1869, with notes on the status of some other genera (Hymenoptera, Cynipoidea: Eucoilidae). *Entomologica Scandinavica* **11**: 428-452.

Nordlander, G. 1981. A review of the genus *Trybliographa* Förster, 1869 (Hymenoptera, Cynipoidea: Eucoilidae). *Entomologica Scandinavica* **12**: 381-402.

Noyes, J. S., Ronquist, F. and Forshage, M. 2011. Hymenoptera: Cynipidae. *Fauna Europea* **v.1.1**: <http://www.faunaeur.org>.

O'Connor, J. P. 2004. A checklist of the Irish Eucoilinae (Hymenoptera: Figitidae) including fourteen species new to Ireland. *Irish Naturalists' Journal* **27**: 382-386.

O'Connor, J. P. and Nash, R. 1998. A review of the Irish Charipidae (Hymenoptera) including nine species new to Ireland. *Irish Naturalists' Journal* **25 (1997)**: 410-412.

O'Connor, J. P., Fergusson, N. D. M. and Nash, R. 2003. A checklist of the Irish Figitidae excluding Charipinae & Eucoilinae (Hymenoptera). *British Journal of Entomology and Natural History* **16**: 229-232.

O'Connor, J. P., Nash, R. and Broad, G. R. 2009. *An annotated checklist of the Irish Hymenoptera*. Dublin: The Irish Biogeographical Society.

Paretas-Martínez, J., Arnedo, M. A., Melika, G., Selfa, J., Seco-Fernández, M. V., Fülöp, D. and Pujade-Villar, J. 2007. Phylogeny of the parasitic wasp subfamily Charipinae (Hymenoptera, Cynipoidea, Figitidae). *Zoologica Scripta* **36**: 153-172.

Paretas-Martínez, J., Ferrer-Suay, M., Kovalev, O., Melika, G., Selfa, J. and Pujade-Villar, J. 2011. Revision of the species of *Dilyta* Förster (Hymenoptera: Figitidae: Charipinae) present in the Holarctic, with description of four new species from the eastern Palaearctic. , 780, 29 38. *Zootaxa* **2780**: 29-38.

Pujade-Villar, J. 2005. On two valid cynipid species: *Saphonecrus gallaepomiformis*(Boyer de Fonscolombe, 1832) n. comb. and *Synergus facialis*Hartig, 1840 (Hym., Cynipidae: Synergini). Butlletí de la Institució Catalana d'Història Natural **72** (2004): 110‑112.

Pujade-Villar, J. and Melika, G. 2000. Notes on *Andricus malpighii* (Adler) valid name to remplace [sic] *Andricus nudus* (Adler) (Hymenoptera: Cynipidae). *Folia Entomologica Hungarica* **61**: 161-162.

Pujade-Villar, J. and Paretas-Martínez, J. 2006. *Phaenoglyphis* "versus" *Hemicrisis*, and the description of a new sculptured species of Charipinae (Hymenoptera: Figitidae). *European Journal of Entomology* **103**: 477-481.

Pujade-Villar, J. and Plantard, O. 2002. About the validity of *Diplolepis fructum* (Rűbsaamen) and some new synonyms in *Diplolepis nervosa* (Curtis). *In*: Pujade-Villar, J. & Ros-Farrė, P. 2001 Review of the uncertain *Neuroterus* species described by Hartig (Hymenoptera: Cynipidae). *Entomofauna* **22**: 405-412.

Pujade-Villar, J., Ros-Farré, P. and Angel Arnado, M. 1998. Phylogenetic position of *Neuroterus anthracinus* (Curtis, 1838) comb.nov. (Hymenoptera: Cynipidae). *Butlleti de la Institucio Catalana d'Historia Natural* **66**: 111-114.

Pujade-Villar, J., Melika, G., Ros-Farré, P., Acs, Z. and Csoka, G. 2003. Cynipid inquiline wasps of Hungary, with taxonomic notes on the western Palaearctic fauna (Hymenoptera: Cynipidae: Cynipinae: Synergini). *Folia Entomologica Hungarica* **64**: 121-170.

Pujade-Villar, J., Barbotin, F., Folliot, R. and Melika, G. 2007. Are *Callirhytis erythrostoma* (Dettmer, 1933) and *C. erythrosoma* (Dettmer, 1933) synonyms of *Callirhytis erythrocepha (*Giraud, 1859) or different species? (Hymenoptera: Cynipidae: Cynipini). *Butlleti de la Institucio Catalana d'Historia Natural* **73**: 61-70.

Pujade-Villar, J., Ferrer-Suay, M., Selfa, J. and Alonso-Zarazaga, M. A. 2011. What is *Alloxysta fulviceps* (Curtis, 1838) (Hymenoptera: Cynipoidea: Figitidae: Charipinae)? *Memoirs of Museum Victoria* **68**: 67-70.

Quinlan, J. 1974. The British Cynipoidea (Hymenoptera) described by P. Cameron. *Bulletin of the British Museum (Natural History), Entomology* **31**: 1-21.

Quinlan, J. 1978a. Cynipoidea. In: Fitton, M. G., Graham, M. W. R. de V., Bouček, Z. R. J., Fergusson, N. D. M., Huddleston, T., Quinlan, J., Richards, O. W., editors. *Kloet and Hincks. A check list of British insects. Part 4: Hymenoptera*: Handbooks for the Identification of British Insects 11(4), ix + 159 pp. p. 63-67.

Quinlan, J. 1978b. Hymenoptera: Cynipoidea: Eucoilidae. *Handbooks for the Identification of British Insects* **Vol. VIII, Part 1 (b)**: 1-58.

Redfern, M. 2006. A new oak gall wasp in Britain. *Cecidology* **21**: 46-48.

Robbins, J. 2007. *Plagiotrochus australis* (Mayr) and *P. coriaceus* (Mayr) (Hymenoptera: Cynipidae) new to Britain. *Cecidology* **22**: 19-20.

Rolfe, R. A. 1881. Notes on the oak galls in the quercetum of the Royal Botanic Gardens, Kew. *Entomologist* **14**: 56-58.

Ronquist, F. 1999. Phylogeny, classification and evolution of the Cynipoidea. *Zoologica Scripta* **28**: 139-164.

Ronquist, F., Nieves-Aldrey, J. L., Buffington, M. L., Liu, Z., Liljeblad, J. and Nylander, J. A. A. 2015. Phylogeny, evolution and classification of gall wasps: the plot thickens. *PLoS ONE* **105**: e0123301. doi: 10.1371/journal.pone.0123301

Ros-Farré, P. and Pujade-Villar, J. 2009. Revision of the genus *Callaspidia* Dahlbom, 1842 (Hym.: Figitidae: Aspicerinae). *Zootaxa* **2105**: 1-31.

Ros-Farré, P. and Pujade-Villar, J. 2013. Revision of the genus *Aspicera* Dahlbom, 1842 (Hym.: Figitidae: Aspicerinae). *Zootaxa* **3606**: 1-110.

Sanders, D. and Van Veen, F. J. F. 2010. The impact of an ant–aphid mutualism on the functional composition of the secondary parasitoid community. *Ecological Entomology* **35**: 704-710.

Spooner, B. M. and Bowdrey, J. P. 2000. Checklist of the British galls and gall-causing organisms 4. Hymenoptera, Part I Cynipinae: preliminary list. *Cecidology* **15**: 41-74.

Stone, G. N. and Sunnocks, P. J. 1992. The hedgehog gall *Andricus lucidus* (Hartig, 1843) confirmed in Britain. *Cecidology* **7**: 30-35.

Stone, G. N., Atkinson, R. J., Rokas, A., Nieves-Aldrey, J.-L., Melika, G., Ács, Z. Csóka, G., Hayward, A. Bailey, R., Buckee, C. and McVean, G. A. T. 2008. Evidence for widespread cryptic sexual generations in apparently purely asexual *Andricus gallwasps*. *Molecular Ecology* **17**: 652-665.

Swanton, E. W. 1912. *British plant galls. A classified textbook of cecidology*. London: Methuen.

Traill, J. W. H. 1873. Oakgalls at Ballater, in June, including *Andricus amenti*, Giraud, new to Britain. *Entomologist’s Monthly Magazine* **10**: 85.

Van Veen, F. J. F., Belshaw, R. and Godfray, H. C. J. 2003. The value of the ITS2 region for the identification of species boundaries between *Alloxysta* hyperparasitoids (Hymenoptera: Charipidae) of aphids. *European Journal of Entomology* **100**: 449-453.

Walker, P. 2001a. *Andricus aries* does have a sexual generation on Turkey oak. *Cecidology* **16**: 94-95.

Walker, P. 2001b. The developing community on the introduced oak, *Quercus cerris*: a catkin gall-forming wasp *Andricus grossulariae* Giraud (Hym. Cynipidae) new to Britain. *Entomologist’s Monthly Magazine* **137**: 145-147.

Walker, P. 2002. Two new records for cynipid oak galls (Cynipidae: Hymenoptera) in Britain. *Cecidology* **17**: 64-67.

1. Liljeblad & Ronquist (1998), Ronquist (1999) and Ronquist *et al.* (2015) employed a series of monophyletic tribes, in the absence of strong evidence for relationships above this level. Synonymy for Cynipidae includes all names that have appeared in the British literature but does not necessarily include all Palaearctic names proposed as varieties or forms.  For complete synonymy please see Melika (2006)*,* also Nieves-Aldrey (2001). It should be borne in mind that future molecular studies may change our understanding of some species concepts and their alternating generations. [↑](#footnote-ref-1)
2. The availability of the name Aulacideini has been questioned and is currently being looked into. This will possibly only be solved by the ICZN. [↑](#footnote-ref-2)
3. *A. pigeoti*, added by Bagnall & Harrison (1930) on the basis of galls only (root collar of *Tragopogon porrifolius*), was synonymised by Eady & Quinlan (1963) with *tragopogonis* but erroneously so with *hieracii* in Fauna Europaea (Nieves-Aldrey, pers. comm.). [↑](#footnote-ref-3)
4. Added by Bagnall (1931) from the gall. Discussed by Bowdrey (1999), who concluded that this record was in error. [↑](#footnote-ref-4)
5. Galls bracts of *Centaurea scabiosa*, but not now considered to be a distinct species (Nieves-Aldrey, 1994). [↑](#footnote-ref-5)
6. Generic classification of oak-associated Cynipini follows Melika & Abrahamson (2002). As sexual and agamic generations have often been described under different names, those based on agamic generation are denoted by the suffix '-a-', those based on the sexual generation by '-s-'. [↑](#footnote-ref-6)
7. Sexual generation catkin galls first recorded by Traill (1873). The asexual generation, described as *giraudianus* Dalla Torre & Kieffer, 1910, has not been recorded in Britain. [↑](#footnote-ref-7)
8. First recorded from the agamic generation, the sexual generation was reported by Walker (2001a). [↑](#footnote-ref-8)
9. *Cynips ambigua* was placed by Bellido *et al*. (2005) in synonymy with *corruptrix* but previously regarded as a distinct species (Melika *et al.*, 2000). Agamic generation reported by Harrison (1930) from Co. Durham & Forfarshire. The sexual generation was described as *ambiguus* forma *elianae* Folliot & Pujade-Villar, 2000. Form *larshemi* van Leeuwen & Dekhuijzen-Maasland, 1958, was described as the sexual generation of *corruptrix* but is now believed to be *improprius* Bellido & Pujade-Villar, 2004, fide Folliot *et al*. (2004), who also produced the true sexual generation of *corruptrix* experimentally. [↑](#footnote-ref-9)
10. Added by Bowdrey (2015); an adult female was identified as this species by G.Melika with the proviso that it might be the sexual generation of a morphologically undescribed species known from DNA sequencing. [↑](#footnote-ref-10)
11. Previously reported by Ormerod (see Cameron, 1893), but reportedly as a leaf gall, so the record is suspect. [↑](#footnote-ref-11)
12. Gall recorded by Bagnall & Harrison (1919) and adult male possibly of this species by Eady & Quinlan (1963). Only the sexual generation is known, inducing bud galls of *Quercus robur*, collected in France (Dalla Torre & Kieffer, 1910). Bagnall & Harrison (1918) mentioned this species from Great Britain but this record requires confirmation (Eady & Quinlan, 1963). A species of uncertain status, type probably lost. [↑](#footnote-ref-12)
13. Rearing experiments by Walker (2002) confirmed *grossulariae* to represent the sexual generation of a lifecycle also involving the asexual generation galls known as *mayri*/ *panteli*, a pairing also indicated by DNA sequence data (Stone *et al*., 2008). [↑](#footnote-ref-13)
14. Nineteenth Century introduction (Adler and Stratton, 1894; Niblett *et al.*, 1932). [↑](#footnote-ref-14)
15. Added by Mosley (1892) from Yorkshire and by Cameron (1893) from Loch Lomond (sexual form). Not recorded subsequently until reinstated as a British species, from London specimens, by Stone & Sunnocks (1992). [↑](#footnote-ref-15)
16. Nomenclature follows Pujade-Villar & Melika (2000). [↑](#footnote-ref-16)
17. Taxonomy follows Melika *et al*. (2000). [↑](#footnote-ref-17)
18. Synonymy of *marginalis* and *quadrilineatus* established by Folliot (1964). [↑](#footnote-ref-18)
19. Gall recorded by Bagnall & Harrison (1918) and Burkill (1933). Modern UK status uncertain. Galls of the sexual generation uncofirrmed in Britain. See also under *testaceipes*. [↑](#footnote-ref-19)
20. No sexual generation known. [↑](#footnote-ref-20)
21. Sexual generation, produced experimentally. [↑](#footnote-ref-21)
22. Maintained with a query; possibly the unconfirmed sexual generation of *A. rhyzomae* (q.v.). If specifically distinct, British records need confirmation. See Melika (2006). [↑](#footnote-ref-22)
23. Added by Cameron (1893) from the gall only. An agamic female in the Morley collection at Ipswich Museum is *quercusradicis*. No recent records. [↑](#footnote-ref-23)
24. Niblett *et al*. (1932) (following Houard) state that this species was accidentally introduced in the 19^th^ Century on *Quercus aegilops*, and never established. [↑](#footnote-ref-24)
25. First recorded from old sexual generation galls by Bagnall & Harrison (1918). Sexual generation also included by Eady and Quinlan (1963), doubtfully British, no confirmed records. [↑](#footnote-ref-25)
26. Added by Eady & Quinlan (1967) on the basis of a single gall found on a road at Kew. No further records. [↑](#footnote-ref-26)
27. Added by Bagnall & Harrison (1918) from the gall only. No further records and rejected as British by Eady & Quinlan (1963). [↑](#footnote-ref-27)
28. Sexual generation (Pujade-Villar *et al*., 2007); synonymy by Stone (in prep.**).**  [↑](#footnote-ref-28)
29. Callirhytis hartigi Förster, 1869 established as the sexual generation by Pujade-Villar et al. (2007)*,* who produce evidence that erythrosoma may be a separate species. [↑](#footnote-ref-29)
30. Recorded by Rolfe (1881) and Askew (1959) but in error fide Eady & Quinlan (1963). [↑](#footnote-ref-30)
31. Galls of the sexual generation were recorded from Durham and Northumberland as *flosculi* by Bagnall & Harrison (1918); there appear to be no further records. [↑](#footnote-ref-31)
32. Both generations reported by Bagnall & Harrison (1918, 1919) from several localities in Northumberland and Tyne & Wear, perhaps in error due to confusion with the similar *quercusfolii*. There are no subsequent records and the species requires confirmation as British. [↑](#footnote-ref-32)
33. This highly invasive, East Asian species has spread across Europe and has recently been reported from Kent and Hertfordshire**.** As the UK has Protected Zone Status for this global pest, apparently successful attempts were made to eradicate *D.kuriphilus* at the two outbreak sites and it should not be formally added to the British list at this stage. [↑](#footnote-ref-33)
34. Galls recorded once by Fitch (1874); no further UK records. [↑](#footnote-ref-34)
35. Form ‘*borealis*’ described by Entwistle & Hails (1997). [↑](#footnote-ref-35)
36. Transferred from *Andricus* by Pujade-Villar *et al*. (1998). [↑](#footnote-ref-36)
37. Described by Bignell (1892), no subsequent records. [↑](#footnote-ref-37)
38. Added based on old agamic gall only, subsequently found at Imperial Wharf and Royal Botanic Gardens Kew, both London (D.G. Notton, pers comm.). Galls introduced *Quercus ilex* and *Q. coccifera*. [↑](#footnote-ref-38)
39. Added based on old agamic gall only. [↑](#footnote-ref-39)
40. Added from sexual generation gall, adults reared (Hancy & Hancy, 2004). Recent records from Devon, Cornwall and Hampshire (Isle of Wight). Catkin galls reported by Biggs (2011) from Isle of Wight as forma *fusifex*, which has no taxonomic status. [↑](#footnote-ref-40)
41. Recorded by Bagnall & Harrison (1918) from the agamic gall; no further records. [↑](#footnote-ref-41)
42. The status of *mayri* as a British species appears to rest solely on three published references to the species: Cameron (1893), subsequently quoted in Connold (1909) and repeated by Swanton (1912). Nowhere does Cameron claim to have evidence of this species’ occurrence in Britain, merely comparing its gall to other British species of *Diastrophus* and *Xestophanes*. As there appear to be no genuine UK records it should therefore be excluded. [↑](#footnote-ref-42)
43. Close to *caninae*, taxonomic status needs revision (note in Fauna Europaea). [↑](#footnote-ref-43)
44. Galls recorded from Co. Durham (Bagnall, 1917), Derbyshire (Fordham, 1917), Northumberland (Bagnall, 1918) and Scotland (Bagnall, 1932). No recent records. [↑](#footnote-ref-44)
45. Synonymised with *Phanacis* (Eady & Quinlan, 1963); re-established by Nieves-Aldrey (1994) but Melika (2006) again treated *Timaspis* as a synonym of *Phanacis*. [↑](#footnote-ref-45)
46. Galls recorded in Norfolk and Derbyshire by Bagnall & Harrison (1934). [↑](#footnote-ref-46)
47. Galls recorded from Norfolk and Surrey by Bagnall & Burkill (1935) but no recent records. Possibly confused in the literature with *Aulacidea follioti* which also galls *Sonchus asper* (Nieves-Aldrey, 2001). [↑](#footnote-ref-47)
48. Galls not distinguishable from those of the smooth form of *nervosa*; records from Scotland, Wales and the Isle of Man are based on galls only. [↑](#footnote-ref-48)
49. Taxonomy follows Pujade-Villar *et al*. (2003). [↑](#footnote-ref-49)
50. This species has hitherto been known as S. gallaepomiformis in Britain but it was shown in Pujade-Villar (2005) that this name refers to another species (of *Saphonecrus*) and the widespread species should be called facialis. Melika (2006) proposed that *gallaepomiformis* (Boyer de Fonscolombe, 1832, *Diplolepis*) should be retained as the valid name but did not make an application to ICZN to overturn the type designation of *gallaepomiformis*. [↑](#footnote-ref-50)
51. Added by Morley (1931); specimen in his collection at Ipswich Museum redetermined as *umbraculus* (Eady, 1952). [↑](#footnote-ref-51)
52. Some Irish records from O’Connor *et al*. (2003). [↑](#footnote-ref-52)
53. Synonymy follows Fergusson (1986). [↑](#footnote-ref-53)
54. Western Palaearctic species were revised by Mata-Casanova *et al.* (2015). [↑](#footnote-ref-54)
55. After the taxonomic changes in the revision by Ros-Farré & Pujade-Villar (2013) it is not entirely certain which nominal species is present in the British material. The authors cite *scutellata* as British, but with a reference to Fergusson (1986), thus not based on reexamination of specimens. [↑](#footnote-ref-55)
56. Nomenclature follows Ros-Farré & Pujade-Villar (2009). [↑](#footnote-ref-56)
57. Transferred from Figitinae to Aspicerinae by Buffington *et al.* (2007). Synonymy follows Fergusson (1986). [↑](#footnote-ref-57)
58. Except where noted, nomenclature follows Menke & Evenhuis (1991) and Ferrer-Suay et al. (2012a,b,c,d, 2013a,b,c,d, 2014a,b, 2015). Tribal subdivisions have been abandoned following Paretas-Martinez *et al*. (2007), who found that Alloxystini was paraphyletic with respect to Charipini. Some Welsh occurrence records from Baker (2013). [↑](#footnote-ref-58)
59. Quinlan (1978*a*) listed 36 species for Britain and Ireland, which Fergusson (1986) reduced to 11, mainly through synonymy. However, Fergusson did not work with much reared material and Evenhuis (1985) and Müller *et al*. (1999) have adopted much narrower species limits than Fergusson, an approach borne out by FVV’s work on the biology and taxonomy of the genus (see Van Veen *et al*., 2003). [↑](#footnote-ref-59)
60. The species identified as *brachyptera* by Müller *et al*. (1999) is actually *apteroidea*, although the true *brachyptera* has also been found in Silwood Park. [↑](#footnote-ref-60)
61. Raised from synonymy with *brevis* by Ferrer-Suay *et al.* (2012*b*). [↑](#footnote-ref-61)
62. Raised from synonymy with *macrophadnus* by Ferrer-Suay *et al.* (2013*a*). [↑](#footnote-ref-62)
63. Synonymised with *fulviceps* by Fergusson (1986) but treated as a valid species by Ferrer-Suay *et al.* (2012*a*) following the advice of FVV. [↑](#footnote-ref-63)
64. *Allotria ruficollis* is listed as a synonym of *fulviceps* in Fauna Europaea, following alternate synonymisations under *erythrothorax* (Quinlan, 1974) and *castanea* (Evenhuis, 1982). In Müller *et al*. (1999) this was recorded as ‘*Alloxysta* f1’. [↑](#footnote-ref-64)
65. Synonymised under *victrix* by Fergusson (1986) but this has not been supported by other workers (Menke & Evenhuis, 1991; Van Veen *et al*., 2003). [↑](#footnote-ref-65)
66. Synonymised under *victrix* by Fergusson (1986) but raised from synonymy by Menke & Evenhuis (1991), a result supported by Van Veen *et al*. (2003). [↑](#footnote-ref-66)
67. Raised from synonymy with *macrophadnus* by Ferrer-Suay *et al.* (2013*a*). [↑](#footnote-ref-67)
68. Considered a *nomen dubium* in Ferrer-Suay *et al.* (2014*a*) but Evenhuis (1982) studied the type and subsequently identified British specimens as belonging to this taxon, so we see no problem with considering it a valid British species. It was listed as *castanea* in Quinlan (1978*a*) but not mentioned by Fergusson (1986) and then recorded again by Müller *et al*. (1999). [↑](#footnote-ref-68)
69. Removed from synonymy with *fulviceps* by Pujade-Villar *et al.* (2011). [↑](#footnote-ref-69)
70. Synonymised under *pedestris* by Fergusson (1986) but considered here to be a valid species (Ferrer-Suay *et al.*, 2012*a*). [↑](#footnote-ref-70)
71. Recorded as a misidentification of *macrophadna* by Fergusson (1986) but erroneously listed as a valid species, occurring in Britain, in Fauna Europaea, but with the comment that the species might be synonymous with *pleuralis*. [↑](#footnote-ref-71)
72. Synonymised under *macrophadna* by Fergusson (1986) but considered here to be a valid species (Ferrer-Suay *et al.*, 2012*a*, following pers. comm. by FVV). [↑](#footnote-ref-72)
73. Raised from synonymy with *brevis* by Ferrer-Suay *et al.* (2012*b*). [↑](#footnote-ref-73)
74. Raised from synonymy with *macrophadnus* by Ferrer-Suay *et al.* (2013*a*). [↑](#footnote-ref-74)
75. Excluded from the British and Irish list by Fergusson (1988) but reinstated by Van Veen *et al*. (2003). [↑](#footnote-ref-75)
76. These species were listed by Quinlan (1978*a*) but not recognised as British or Irish by Fergusson (1986) and not found by FVV. [↑](#footnote-ref-76)
77. Listed by Fergusson (1986) as a synonym of *macrophadnus*, the type material of *ignorata* has not been located and it has not been possible to interpret the name (Ferrer-Suay *et al.*, 2012*a*). [↑](#footnote-ref-77)
78. Fergusson (1986) established *Hemicrisis* (including only *ruficornis*) as a synonym of *Phaenoglyphis*, it was then raised from synonymy by Ronquist (1999) only to be synonymised again with *Phaenoglyphis* by Pujade-Villar & Paretas-Martínez (2006). [↑](#footnote-ref-78)
79. Listed by Quinlan (1978*a*) as a species of *Alloxysta*, not mentioned by Fergusson (1986), recorded as British by Baker (2013) based on specimens identified by M. Ferrer-Suay. [↑](#footnote-ref-79)
80. *Phaenoglyphis pubicollis* (Thomson, 1877, *Allotria*) was resurrected as a valid species by Pujade-Villar & Paretas-Martinez (2006). The authors did not examine material from a wide range but state that *pubicollis* ‘is only represented by type material’, implying that the real *ruficornis* is the widespread one. [↑](#footnote-ref-80)
81. Eucoilinae has been a subfamily of Figitidae rather than a family of its own since Ronquist (1999). Tribal classification follows Forshage & Nordlander (2008). Synomymy and distribution data taken from Quinlan (1978*b*), Nordlander (1978, 1980, 1981) and Forshage & Nordlander (2008), supplemented by Fauna Europaea and MF’s identifications of material in BMNH. Some Irish records from O’Connor (2004). [↑](#footnote-ref-81)
82. Supposed English specimens in BMNH are all *Disorygma curtum* (det. MF). [↑](#footnote-ref-82)
83. Synonymy from Nordlander (1980). [↑](#footnote-ref-83)
84. Treated as *Episoda xanthoneura* by Quinlan (1978*b*). [↑](#footnote-ref-84)
85. Treated as *Ganaspis subnuda* by Quinlan (1978*b*). [↑](#footnote-ref-85)
86. Synonymy mostly from Nordlander (1981). Several new species of *Trybliographa* are present in Britain and several new synonymies will result from MF’s unpublished revision. Some changes have been made in anticipation of this publication as they affect names described from British specimens. [↑](#footnote-ref-86)
87. Generic combination not yet formally published. [↑](#footnote-ref-87)
88. Unpublished synonymy. [↑](#footnote-ref-88)
89. Recently removed from synonymy with *trichopsila* in Forshage *et al.* (2013). [↑](#footnote-ref-89)
90. This name will be placed in synonymy. [↑](#footnote-ref-90)
91. Subgeneric classification was finally abandoned and all subgeneric names considered mere synonyms in Forshage & Nordlander (2008). [↑](#footnote-ref-91)
92. Usually regarded as a species of *Trybliographa* but published as a new combination in *Kleidotoma* by Jonsell *et al*. (1999) in an ecological paper and listed as such in Fauna Europaea. The *ciliaris* sensu auctt. nec Zetterstedt, refers to various small *Trybliographa* spp. [↑](#footnote-ref-92)
93. Name preoccupied by *Kleidotoma tetratoma* (Hartig, 1841, *Cothonaspis*). [↑](#footnote-ref-93)
94. Recorded from Britain in Fauna Europaea but in error. [↑](#footnote-ref-94)
95. Synonymy taken from Nordlander (1978). [↑](#footnote-ref-95)
96. *Rhoptromeris nigriventris* was listed by Quinlan (1978*b*) but this was most likely a mistake. Nordlander (1978) mentions that some Swedish paratypes were deposited in BMNH. There are no British or Irish specimens identified as *nigriventris* in the collection. [↑](#footnote-ref-96)
97. Only a single male specimen has been found amongst British material; it is not identifiable to species level with our current state of knowledge, and is listed here as a record of the genus from Britain. [↑](#footnote-ref-97)
98. *Melanips* has been transferred to Aspicerinae by Buffington *et al.* (2007). [↑](#footnote-ref-98)
99. Removed from synonymy with *Sarothrus* by Ronquist (1999). [↑](#footnote-ref-99)
100. The following species were included in Quinlan (1978b) but not by Fergusson (1986). [↑](#footnote-ref-100)
